# Ago2 Immunoprecipitation Identifies Predicted MicroRNAs in Human Embryonic Stem Cells and Neural Precursors

**DOI:** 10.1371/journal.pone.0007192

**Published:** 2009-09-28

**Authors:** Loyal A. Goff, Jonathan Davila, Mavis R. Swerdel, Jennifer C. Moore, Rick I. Cohen, Hao Wu, Yi E. Sun, Ronald P. Hart

**Affiliations:** 1 Rutgers Stem Cell Research Center and the W.M. Keck Center for Collaborative Neuroscience, Rutgers University, Piscataway, New Jersey, United States of America; 2 Computer Science and Artificial Intelligence Laboratory and The Broad Institute, Massachusetts Institute of Technology, Cambridge, Massachusetts, United States of America; 3 Departments of Molecular & Medical Pharmacology and Psychiatry & Behavioral Sciences, MRRC at UCLA Neuropsychiatric Institute, Los Angeles, California, United States of America; Institut Curie, France

## Abstract

**Background:**

MicroRNAs are required for maintenance of pluripotency as well as differentiation, but since more microRNAs have been computationally predicted in genome than have been found, there are likely to be undiscovered microRNAs expressed early in stem cell differentiation.

**Methodology/Principal Findings:**

SOLiD ultra-deep sequencing identified >10^7^ unique small RNAs from human embryonic stem cells (hESC) and neural-restricted precursors that were fit to a model of microRNA biogenesis to computationally predict 818 new microRNA genes. These predicted genomic loci are associated with chromatin patterns of modified histones that are predictive of regulated gene expression. 146 of the predicted microRNAs were enriched in Ago2-containing complexes along with 609 known microRNAs, demonstrating association with a functional RISC complex. This Ago2 IP-selected subset was consistently expressed in four independent hESC lines and exhibited complex patterns of regulation over development similar to previously-known microRNAs, including pluripotency-specific expression in both hESC and iPS cells. More than 30% of the Ago2 IP-enriched predicted microRNAs are new members of existing families since they share seed sequences with known microRNAs.

**Conclusions/Significance:**

Extending the classic definition of microRNAs, this large number of new microRNA genes, the majority of which are less conserved than their canonical counterparts, likely represent evolutionarily recent regulators of early differentiation. The enrichment in Ago2 containing complexes, the presence of chromatin marks indicative of regulated gene expression, and differential expression over development all support the identification of 146 new microRNAs active during early hESC differentiation.

## Introduction

Specific populations of microRNAs are uniquely expressed in mouse embryonic stem (ES) cells [1], mouse or human embryonic carcinoma cells [2], and human ES cells [3], [4]. The presence of selected microRNAs in stem cells and their disappearance during differentiation suggest roles in suppressing pluripotency and/or restricting cell differentiation. Indeed, knockout mice lacking Argonaute2 (Ago2), the catalytic component of the RNA-induced silencing (RISC) complex, exhibit severe defects in neural development, including the failure to close neural tube [5]. Genomic silencing of DGCR8, an RNA-binding protein essential for processing microRNAs, completely prevents mouse ES cells from differentiating into embryoid bodies, suggesting that microRNAs are required to inhibit ES self-renewal [6]. In one example, mir-302 is induced by Oct4 (POU5F1) and Sox2 in hESC, in turn suppressing cyclin D1 and thereby (along with other target mRNAs), increasing the fraction of cells in S phase and stimulating cell cycle [7]. Alternatively, mir-145, induced upon differentiation, represses pluripotency by inhibiting production of Oct4, Sox2, and KLF4 [8]. From these studies we conclude that microRNAs are important early in stem cell differentiation and are likely to be required for differentiation mechanisms [reviewed by: 9].

While about 700 human microRNAs have been identified, the total number of microRNA genes has been predicted to be from 1,000 per genome [10], [11], [12] to as many as 10,000 [13]. Perhaps some microRNAs are exclusively expressed in specific tissues or differentiation stages, functioning transiently during development. We propose that most previously identified microRNAs are expressed universally or in stable, adult tissues. Therefore, early stages of development are more likely to express microRNAs that are present only transiently or in specific cell stages. One study proved that this can be an effective strategy by using deep sequencing to screen small RNA sequences in hESC, finding 13 new microRNAs [14]. Perhaps existing discovery methods, including earlier deep sequencing approaches, were not sufficiently sensitive to detect low concentrations of these microRNAs. Since we wish to study the function of microRNAs during early neural precursor development, we chose to apply the highly sensitive and accurate method of Sequencing by Oligo Ligation and Detection (SOLiD) to cultures of hESC and their neural precursor derivatives. We found not only that quantifying microRNAs by counting sequences is a sensitive method for evaluating microRNA expression, but that new transcripts could be identified that mapped to genomic loci predicted to encode previously unknown microRNAs. However, sequencing alone cannot distinguish microRNAs from other families of small, non-coding RNA (ncRNA). Since microRNAs must be associated with a RISC complex to have function, we identified candidate microRNAs by identifying them in immunoprecipitated Ago2-containing complexes.

Known populations of microRNAs are strongly conserved across species, but should we expect newly-identified microRNAs to be similarly conserved? Most studies that have searched for microRNAs have attempted to extend this conservation when identifying putative genes among populations of small ncRNA. One of the accepted criteria for microRNA identification is phylogenetic conservation across species [15]. This criterion would automatically exclude any potentially species-specific microRNAs and skew evolutionary studies to identify a high percentage of conserved microRNAs by definition. However, many microRNAs are not conserved and are lineage- or species-specific [12], [16], [17], [18], [19], [20]. The expansion and evolution of microRNAs have been linked to both body plan innovation and vertebrate morphological complexity throughout evolution [21], [22], [23], suggesting that some newly-described microRNAs may lack conservation with distant species. Furthermore, there is evidence that microRNA innovation is an ongoing process [21], [24]. MicroRNAs have been identified as key developmental regulators, modulating cell specificity and tissue identity. Based on this fundamental developmental function, we speculate that species-specific microRNAs function in a manner that would contribute to the uniqueness of a species. For example, Cao *et al.* [20] describe a hES cell-specific population of microRNAs that evolved in primates only some 5 million years ago. These microRNAs are differentially expressed during cell differentiation and probably exert a posttranscriptional regulatory role specific to primate stem cell self-renewal and differentiation. Therefore, we predict that new microRNAs expressed early in human development would not likely be conserved across most species.

By incorporating SOLiD deep sequencing of large numbers (>100 million) of small RNAs from hESC and by adapting these results to an algorithm designed to match small RNA reads with genome sites consistent with the established mechanism of microRNA processing [25], we identified over 800 predicted microRNAs. To validate these predictions we assayed Ago2-containing complexes and found 146 predicted microRNAs as enriched by immunoprecipitation. Finally, we compared small RNA SOLiD sequencing results from multiple, independently-derived hESC lines to ensure expression across biologically variable samples. While a similar approach has been tried previously with lower numbers of small RNA sequences [14], ours is the first study to combine ultra-deep sequencing, model-based precursor prediction, and validation in Ago2-containing complexes. Initial analysis of new microRNAs suggests that they are expressed in a more biologically-specific manner and are less conserved than previously-identified microRNAs.

## Methods

### hESC Cultures

HSF1 and HSF6 cultures were prepared and differentiated at UCLA as previously described [26]. H1 and RG7 cultures were grown at Rutgers using the feeder free methodology described by Ludwig et al. [27]. H1 and H9 were obtained from the National Stem Cell Bank (WiCell). The derivation and characterization of the novel hESC line RG7 will be described elsewhere (Moore et al., in preparation). Summarizing those results, H9 (46XX) and RG7 (46XY) were found to have normal karyotypes by G-banding and array comparative genomic hybridization (aCGH; Agilent 244K chips) but H1 (46XY) exhibited genome duplications on chromosomes 12 and 17, as assessed by preliminary aCGH. Alterations in H1 genome have been seen previously over extended passaging [28]. All lines had demonstrable pluripotency by gene expression studies, immunofluorescence, and cell sorting assays (Moore et al., in preparation). Since H1 was used initially in our lab, initial deep sequencing datasets came from this cell line. As we learned about the genomic duplications on H1, we switched to RG7 to retain the XY genotype while providing a more normal genome.

Cells were passaged with 1 U/mL dispase every 7 days at a splitting density of 1∶6 to 1∶10, so that the resulting cultures are 80–90% confluent by the 7^th^ day of passage. In order to obtain Neural Stem Cells (NSC), a protocol based on previously published, adherent neural differentiation protocols has been developed [29], [30]. On day 0, undifferentiated cells were pre-conditioned with Neural Induction Medium (NIM), which consists of a 1∶1 ratio of DMEM/F12 and NeuralBasal media (Gibco, Life Technologies, Inc.) containing 1× B27 (without retinoic acid; Gibco) and 1× N2 supplement (Gibco) for 2 days. After 48 hours the pre-conditioned cells were passaged using 0.5 U/mL papain (Sigma-Aldrich) and transferred to dishes coated with ¼ the recommended concentration of Matrigel and grown for 2 more days in NIM. On day 5, the medium was changed to Neural Precursor Media (NPM). NPM is a 1∶1 ratio of DMEM/F12 and NeuralBasal media that contains 0.5× B27 and N2, as well as 20 ng/mL bFGF (Invitrogen, Life Technologies, Inc.). When cells grown in NPM reached 90–100% confluent, they were passaged 1∶2 on Matrigel diluted ¼ and the medium was refreshed every other day. On day 7–10, differentiation into neuronally restricted precursors (NRP) was induced by withdrawing bFGF and adding 10 ng/mL BDNF, as described in Nat et al. [29]. All culturing of RG7 was performed without federal funding in a laboratory built and operated with New Jersey state funding. Procedures were approved by the Rutgers institutional embryonic stem cell research oversight (ESCRO) committee. Total cellular RNA was prepared using Trizol and assessed by Bioanalyzer (Agilent, Santa Clara, CA) and Nanodrop (ND-1000, Thermo Fisher, Waltham, MA).

### iPS cells

Cultures of iPS(Foreskin)-1 (Clone 1) were obtained from the National Stem Cell Bank (WiCell) and cultured as described above for hESC. Morphologically, the cells were enlarged and flattened compared with hESC cultures and a larger proportion of the cells did not grow in defined colonies. However, the cultures readily differentiated into NSC as defined by immunocytochemistry (not shown).

### Immunofluorescence

Cells from each of the 3 differentiation stages (ESC, NSC, and NRP) were plated on coverslips and cultured as described. After fixation with 4% paraformaldehyde, the cells were permeabilized and blocked. The cells were incubated with primary antibody diluted in blocking buffer for at least 1 hour and AlexaFluor conjugated secondary antibodies (Molecular Probes) were used to visualize the antibodies by fluorescence microscopy. Antibodies, sources, and final concentrations used were: Oct4 (Millipore MAB4401, 0.5 µl/ml), SSEA4 (Millipore MAB4304, 1.0 µl/ml), SSEA1 (Millipore MAB4301, 1.0 µl/ml), Sox2 (Millipore AB5603, 1.0 µg/ml), Nestin (Millipore MAB5326, 0.5 µg/ml), and TuJ1 (Aves TUJ-S, 1.0 µg/ml).

### Microarrays

RNA samples, 500 ng each, prepared by Trizol extraction and alcohol precipitation, were labeled and hybridized with Illumina Human-6 beadchips at the Burnham Institute genome center. RNA integrity was assessed by Bioanalyzer (Agilent) and concentrations determined by Nanodrop spectrophotometry. Results were interpreted using GeneSpring v7.4 (Agilent) and/or R/Bioconductor (http://www.bioconductor.org). Data are available through GEO (GSE15206).

### SOLiD Sequencing

Total cellular RNA samples (100 ng) were processed into sequencing libraries using the Small RNA Expression Kit (SREK, Applied Biosystems). Briefly, RNA was ligated overnight with the “A” adapters from the kit, reverse transcribed, RNAse H-treated, and PCR-amplified before size selection on polyacrylamide gels to contain 18–30 nt of inserted sequences. After quantification by qPCR, libraries were amplified onto beads using emulsion PCR, deposited on slides, and sequenced using the SOLiD v 2 sequencing system (Applied Biosystems) at the Waksman Genomic Laboratory, Rutgers University. Results were obtained as “good” and “best” beads (as judged by the SOLiD sequence analysis software) as colorspace FASTA files (csfasta). In one experiment (identified as experiment 2 in Table S1), ten different samples were distinguished by adding unique “barcode”-labeled amplification primers (provided as part of the SOLiD SREK kit). All ten libraries were mixed and sequenced on a single slide. After the usual sequencing reactions, a second set of reactions decoded the barcode, matching a bead sequence with the identity of the sample. Primary H1 small RNA sequencing data from experiment 1 are available from the NIH short reads archive (accession SRA008181.1).

### Data Analysis

Individual colorspace sequences were loaded into a MySQL database from which distinct colorspace sequences were extracted. These were aligned with human genome (build 18) in colorspace using SHRiMP v1.1.0 software (http://compbio.cs.toronto.edu/shrimp/) with default parameters set except the maximum length (25 nt) and the maximum number of matches (limited to 10). Alignments were loaded into a MySQL database. To increase signal above noise, we conservatively selected only those alignments corresponding to beads sampled a minimum of 10 times in any of the libraries. Overlapping alignments were condensed to distinct genomic intervals and read counts were aggregated for each interval. Summarized, individual intervals were extracted for each culture condition (H1 ESC or H1 NSC, see Results) for each chromosome. Colorspace alignment data were then reshaped (see Methods S1) and used as input for a probabilistic model for microRNAs identification from deep sequencing reads. Briefly, miRDeep [25] extracted potential precursors and attempted to fold these into hairpin sequences. The potential precursors were then aligned with individual reads and positions along the potential precursor were integrated with sequence counts with hairpin folding potential to identify microRNA-like loci. Predicted loci matching known microRNAs, other RNA genes, or repeated chromosomal regions were removed using Genome Browser software (http://genome.ucsc.edu). Except where noted, BLAST was used to match known and predicted microRNAs to obtain counted expression levels [31] as described in the Methods S1.

### qPCR

Predicted microRNAs were assayed in small RNA libraries by SYBR green qPCR (SYBR Green Master Mix, Applied Biosystems) using one microRNA-specific primer and one primer specific for the library (5′-CTC CTG TAC GGC CAA GGC G-3′). Known human microRNAs were assayed using the Human microRNA TaqMan low density array (Applied Biosystems).

### Immunoprecipitation

Lysed cell extracts were immunoprecipitated with Anti-human Ago2 antibody [32] (Ascenion GmbH, Helmholtz Zentrum, München) or rabbit IgG using protein G Sepharose beads. Each cell extract was spiked with a known quantity of synthetic microRNA (NCode Control RNA, Invitrogen) to provide an endogenous control to correct for differences in library preparation or sequencing depth. After elution from beads, RNA was prepared using Trizol reagent and used to construct SOLiD libraries. A detailed protocol is provided in the Methods S1.

## Results

**Figure 1:**
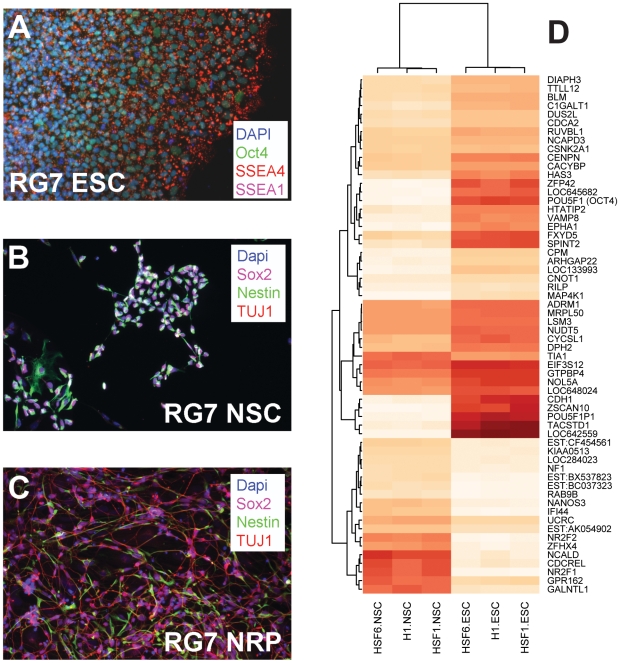
Characterization of hESC cultures. For each hESC line used, culture stage was assessed by immunocytochemistry and culture morphology. As an example, the RG7 hESC line exhibited markers consistent with pluripotency (SSEA4) but not differentiation (SSEA1) in the ESC condition (panel A), expressed nestin but not β-III-tubulin (TuJ1) in the neural stem cell (NSC) stage (panel B), and expressed β-III-tubulin with lesser levels of nestin in the neural-restricted precursor (NRP) stage (panel C). In addition, the ESC cultures grew as colonies, the NSC grew as isolated groups of cells, and the NRP produced processes reminiscent of neurons. To confirm this interpretation, mRNA microarrays were run on single samples of H1 ESC and NSC cultures to compare with HSF1 and HSF6 culture samples as previously defined [26]. Hierarchical clustering of all detected genes shows a clear hierarchical clustering of samples by culture stage. Here, this grouping is highlighted using genes distinguishing ESC from NSC (Student's t-test by stage, 5% false discovery rate [FDR]; panel D).

To search for evidence of additional microRNAs in H1 human embryonic stem cell cultures (hESC), we prepared RNA samples for deep sequencing using the SOLiD system for massively-parallel sequencing by ligation [33], [34]. The differentiation status of hESC cultures was assessed by culture morphology and by immunohistochemistry for standard markers (Figure 1A–C). ESC cultures (Figure 1A) were positive for SSEA4 and Oct4 but not SSEA1, consistent with pluripotency. NSC cultures (Figure 1B, grown in neural induction medium including bFGF) expressed nestin but lacked β-III tubulin (TUJ1), and NRP cultures (Figure 1C, grown in neuronal differentiation medium lacking bFGF but including BDNF) exhibited long processes and mostly expressed β-III tubulin and not nestin. To support this interpretation, Illumina human-6 microarrays were run on single biological replicates of three cell lines (H1, HSF1, and HSF6) under two conditions (ESC, NSC). HSF1 and HSF6 were previously studied for their divergent NSC differentiation patterns [26]. Results are consistent with a pluripotent status for ESC and a neural precursor status for NSC. Focusing on 60 genes that were significantly different between ESC and NSC conditions (t-test, 5% false discovery rate [FDR], ≥1.5-fold different), individual cell lines clustered appropriately by their culture condition (Figure 1D). Within the full set of results (available from NIH GEO accession GSE15206), pluripotent-specific genes such as POU5F1 (OCT4), NANOG, LIN28, DNMT3B (ICF), GABRB3 (MGC9051), and GDF3 were all reproducibly expressed at higher levels in ESC than NSC cultures, agreeing with previous expression studies in hESC lines [35]. NSC cultures expressed higher levels of many mRNAs previously identified as specific for human NSC, including PAX6, FOXG1B, TH, NKX2-2, CHAT, TPH2, HDAC6, IL11RA, COL2A1 [26], [36] (including genes not found to be significantly different due to cell line to cell line variance). The highest relative NSC expression markers included NR2F1, NR2F2, SEPT5, ZFHX4, and GPR162. We conclude that the cultures sampled as ESC and NSC exhibit the appropriate phenotypes.

Small RNA sequences were determined using SOLiD on libraries prepared from ESC or NSC staged H1 hESC cultures, producing an average of 55 million 25-nucleotide sequences for each of the two conditions (Table S1, Experiment 1). To search for previously known microRNAs within these results, we ran a simple text-matching query to count the number of sequences perfectly matching each of the human mature microRNAs found in miRBase v11 [37]. Of the 733 available known mature microRNA and “star” sequences, 598 microRNAs were identified among our results.

The resulting counts of small RNA sequences detected ideally reflect expression levels of individual microRNAs. This technique of digital gene expression (DGE) has been found to be a sensitive method for detecting microRNAs but one with some sequence bias based on library preparation methods [38]. Comparing various DGE protocols, ratios among counts of sequences were found to be consistent, meaning that differences between multiple samples assayed by the same method should be accurate [38]. A total of four SOLiD assays were run on multiple hESC cell lines and stages (Table S1). A second slide made use of the multiplexing capability of the SOLiD system to sequence multiple samples at about 7.5 million sequences each by using primers having different barcodes. A third slide sequenced three stages of the novel hESC line RG7, including a “terminally differentiated” (NRP) culture, with an average of 46 million sequences per sample. Is counting of SOLiD-detected sequences an accurate method for measuring microRNA concentrations? We assayed two developmental stages (ESC and NSC) of one cell line (RG7) using both SOLiD and TaqMan microRNA qPCR (File S1), which is generally accepted as an accurate method for measuring relative RNA concentrations. We found good correlation between methods when comparing fold-change from ESC to NSC (r = 0.730, Pearson). We conclude that counting SOLiD sequences exhibits similar accuracy as qPCR over a broad range of concentrations. Therefore, this method is acceptable for judging relative quantities of sequences as potential mature or star strands within a precursor structure.

We performed hierarchical clustering to characterize cell lines and culture stages using counts of all detected known microRNAs. Selecting a list of 82 microRNAs as being significantly different between ESC and NSC cultures across all cell lines (t-test, 5% false discovery rate [FDR], ≥1.5-fold), results were displayed as a heat map, retaining the dendrogram of relationships among samples calculated from all expressed microRNAs, to demonstrate that cultures were distinctly different by culture stage more than by cell line (Figure S1). Within each culture condition group, the RG7 NRP sample was most dissimilar compared with NSC and NPC cultures, but the H1 embryoid body (EB) culture was more like H1 ESC than other ESC cultures, consistent with a predominant cell line difference. The H1 cell line has been found to contain several genomic deletions (see Methods) and therefore may represent a true biological outlier compared with the other lines. This identifies known microRNAs that are differentially expressed between the NSC/NPC precursor stage and the terminally differentiated NRP stage.

H1 hESC small RNA sequencing results from Experiment 1 were used to predict genomic microRNA precursors with miRDeep [25]. This algorithm was designed to screen deep sequencing reads that have been aligned to genome for model properties consistent with Drosha/Dicer substrate processing. SOLiD sequencing produces reads in “colorspace,” where adjacent pairs of nucleotides are detected using a color-coded fluorescent signal indicating a 2-nt sequence selected by ligation [33], [34]. The color-coded reads are interpreted as specifying the “interval” between two adjacent bases. In alignments with genomic sequences, two successive colors must be interpreted consistently since they share a common nucleotide that is interrogated twice. Inconsistent color patterns cannot be aligned to reference genome and thus are discarded, improving the quality of the aligned colorspace sequences. We aligned reads to human genome in colorspace using SHRiMP, which rapidly identifies potential homologies and extends them with a Smith Waterman algorithm [39]. The Smith-Waterman method is a well-established dynamic programming algorithm for finding the optimal local alignment between two sequences using a substitution matrix and a gap-scoring system. Approximately 110 million reads (Table S1, experiment 1) produced 591 million alignments. Alignments were distributed across all chromosomes (Figure S2).

**Figure 2:**
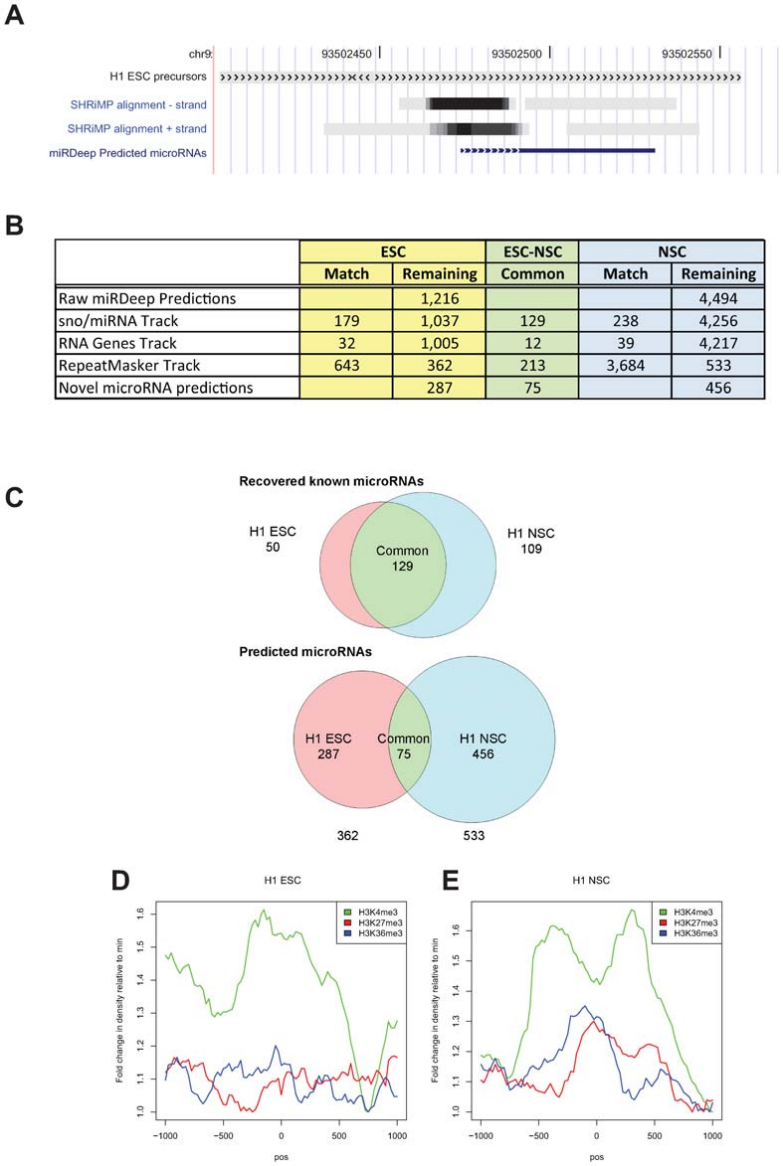
Computationally-predicted microRNAs. A. Example SHRiMP alignment of small RNA sequences, shown here as grayscale “wiggle” tracks on the genome browser, were used to predict a potential microRNA structure. One track is shown for each chromosomal strand. For the miRDeep-predicted microRNA track, the dashed line represents the predicted mature microRNA sequence (aligning with the most frequent small RNA sequence from the wiggle tracks) and the solid line represents the remaining predicted hairpin precursor. B. Table showing numbers of predicted microRNAs produced by miRDeep based on small RNAs derived from ESC or NSC and those subtracted by overlap with the sno/miRNA track, the RNA genes track, or the RepeatMasker track of the genome browser. The remaining predictions shown at bottom constitute an aggregated group of 818 predicted microRNAs. C. Venn diagram showing distinct known (top) and predicted (bottom) microRNAs derived by miRDeep from ESC or NSC cultures as well as the numbers in common between the two conditions. Fewer predicted microRNAs were identified as common to ESC and NSC than known microRNAs. D. ChIP-Seq read densities derived from H9 ESC cultures [41] flanking the aggregated start positions for H1 ESC predicted microRNA precursors. See legend for color specific to each modified histone mark. E. ChIP-seq read densities derived from H9 ESC cultures flanking the aggregated start positions for H1 NSC predicted microRNA precursors.

To focus on high-confidence alignments, we conservatively selected those having 10 or more overlapping SOLiD small RNA reads aligned with a single genomic location. This was done in two separate datasets—one derived from H1 ESC and the other from H1 NSC samples, reasoning that expression and alignment patterns of one condition might obscure those from the other condition. We do not believe that the sequences selected were due to PCR over-amplification because most sequences aligning in overlapping positions varied in their terminal nucleotide position (not shown). A large number of reads were excluded by selecting 10 or more reads per genomic location—31,773,857 reads were excluded for ESC and 37,779,985 for NSC. Selected alignments, condensed by location, were used to extract surrounding putative precursor sequences from genome. These precursors were folded with RNAFold [40] to identify characteristic hairpin structures. Individual small RNA reads, now translated from colorspace to DNA sequence, were re-aligned with putative precursor segments using BLAST [31]. Results of all analyses were input to miRDeep [25]. Using a cut-off log-odds score of 1.0 (that is, results that are 10-fold more likely than random sequence to match the form of a predicted microRNA precursor, according to the miRDeep algorithm [25]), we obtained 1,216 raw candidates from ESC and 4,494 candidates from NSC (Figure 2). A table of all consolidated sequences comprising all the putative precursors along with genomic alignment positions is found in File S2. These predictions covered all chromosomes with NSC predictions exhibiting higher frequencies on chromosomes 1 and X (Figure S2). One example alignment and predicted precursor position is shown in Figure 2A. The grayscale wiggle track of SHRiMP-aligned colorspace sequences identifies two adjacent regions—an interval with more aligned sequences (darker) near an interval with less aligned sequences (lighter), potentially representing mature and star sequences derived from a microRNA precursor. Indeed, after RNAFold and miRDeep computation, a single predicted microRNA and precursor (bottom) is selected.

As an internal control for the prediction process, we chose not to remove sequence reads matching known microRNAs during the prediction phase. Among the miRDeep-derived candidates there are 179 and 238 known microRNAs or snoRNAs from ESC and NSC, respectively. These were removed from predictions using the Tables function of the UCSC genome browser (http://genome.ucsc.edu). Similarly, prediction sites matching the RNA Genes track of the browser were removed, eliminating fragments of rRNA, tRNA, snoRNA, or other classes of small RNA. Finally, prediction sites matching the RepeatMasker track of the genome browser were removed so that we could focus on non-repeated genomic loci. After filtering, a total of 818 predicted microRNA genes were identified from the two conditions, ESC and NSC. Of these, 75 predictions appeared in both conditions, leaving 287 predictions specific for ESC and 456 specific for NSC (Figure 2B). Comparing miRDeep log-odds score distributions for recovered known microRNAs and predicted microRNAs, there is a large degree of overlap but the predicted microRNA population includes a lower range of scores (Figure S3). Perhaps some of these 818 predicted microRNAs could be other classes of small ncRNA.

As expected, a larger proportion of these 818 predicted microRNA genes were identified from data unique to a single developmental stage than was true for known microRNAs (Figure 2C, Files S2 and S4). This agrees with the hypothesis that previously identified microRNAs are more commonly expressed in multiple tissues or stages of development. Newly-identified microRNAs are likely to be more specific to tissue or developmental stage.

If these 818 predicted microRNA genes are expressed and/or regulated during early stem cell differentiation, their genomic positions should be phased with respect to chromatin marks, particularly those characteristic of developmental regulation. Using publicly-available ChIP-Seq data from the ESC stage of the H9 cell line [41], we overlapped the start site and direction of all predicted precursors from each class (ESC or NSC; Figure 2D–E). For this summary analysis we did not examine potential clusters of microRNAs, presence of microRNA-encoding loci in introns, or the possibility of distant transcription start sites, all of which could confound a more detailed interpretation. However, the precursors predicted from ESC had a phased peak of H3K4me3 with local maximum near ∼100 bp upstream of the 5′ end of the precursor and local minima ∼500 bp upstream and ∼800 bp downstream. No peaks were observed for H3K27me3 or H3K36me3. This pattern is similar to a plot summarizing ChIP-Seq marks for 93 known microRNAs exclusively expressed in hESC (not shown). The ChIP alignment pattern was quite distinct for NSC-derived predictions in ESC cultures. The H3K4me3 track had local maxima at ∼500 bp upstream and ∼400 bp downstream of the precursor 5′ end with a dip in the track near the 5′ end. To a lesser degree, H3K27me3 and H3K36me3 tracks appear to peak upstream of the precursor. Since H3K4me3 is normally considered to be indicative of active expression, the presence of marks overlapping predicted transcriptional precursors for the ESC predictions in ESC cells is consistent with gene expression. However, there is no clear indication of transcription by the H3K36me3 marks for this class of predictions. On the other hand, increased numbers of marks for H3K27me3 for the NSC predictions in ESC might indicate repression of at least a subset of the predicted precursors. Since H3K36me3 is also found surrounding predicted precursor sites, another subset may be transcribed or it may represent stalled transcription. Overall, a clear presence of epigenetic marks aligning with predicted precursor locations on genome is consistent with regulated expression during early differentiation.

As a validation strategy for potential biological function of these predicted microRNAs, we searched for small RNAs that could be associated with RISC by immunoprecipitation (IP) with anti-Ago2. Ago2 is one member of the Argonaute family found in RISC complexes [42]. Available evidence suggests that binding among Ago family members is not sequence-specific [43], [44] and so Ago2 binding was expected to serve as a proxy for binding with all Ago proteins. We prepared small RNAs from immunoprecipitated cytoplasmic extracts of hESC or NSC cultures from the RG7 line. Preliminary experiments used qPCR and primers specific for a subset of the predicted microRNAs to test Ago2 IP samples. Out of 87 predicted microRNAs tested, 46 were found to be enriched by Ago2 IP over IgG IP (File S3). Based on this pilot experiment we expanded our search using deep sequencing of the IP samples. Small RNAs were sequenced and those predicted microRNAs that were enriched at least 4-fold over IgG controls were selected, yielding 146 new microRNAs (Files S2 and S5). A similar analysis of known microRNAs found 609 that were at least 4-fold enriched by Ago2 IP (File S2, also see Methods S1). Interestingly, while the majority of Ago2 IP-selected microRNAs were expressed linearly with their concentration within the Ago2 IP sample, several outliers were observed (Figure S4). From this observation we conclude that Ago2 IP does not always merely reflect cellular concentration and therefore it may be more indicative of microRNA function than measurements of microRNA concentrations in the cell.

**Figure 3:**
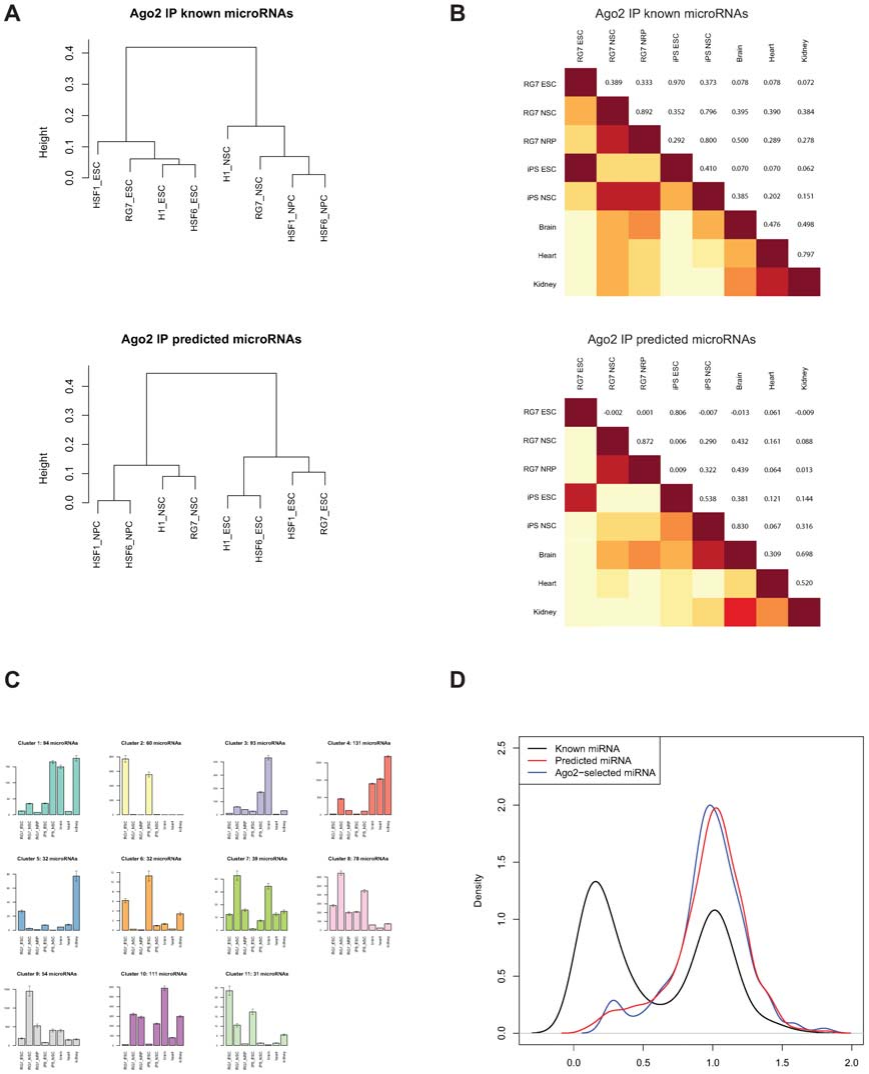
Ago2 IP-selected microRNAs. A. Hierarchical clustering dendrogram showing Pearson correlation between ESC and NSC stages of four hESC lines: H1, HSF1, HSF6, and RG7. Expression was determined by counting occurrences based on the list of 609 known or 146 predicted microRNAs found to be Ago2 IP enriched. Counts were normalized to the total number of sequences per sample. For both known microRNAs (top) and predicted microRNAs (bottom), the primary distinction between samples is based on cell stage. This demonstrates that each group of microRNAs is uniformly expressed in a group of biologically distinct cell lines. B. Pearson correlation tables of microRNA expression across development. Using the list of 609 known microRNAs (top) or 146 predicted microRNAs (bottom) selected from the Ago2 IP enriched lists, expression levels were determined by counting sequences and correcting for total numbers of sequences per sample. Known microRNA expression levels (top) distinguished all three adult tissues from a pluripotent group (RG7 ESC and iPS cells obtained from WiCell) and also from a neural lineage group (RG7 NSC, RG7 NRP, and iPS-derived NSC). However, predicted microRNAs included the adult brain sample in a cluster containing iPS NSC and, to a lesser extent, RG7 NSC and NRP. C. The same dataset was K-means clustered to isolate expression patterns. Based on minimizing the mean sum of squares fit (Figure S6), k = 11 clusters were selected. Shown here are the cluster means±SEM, color-coded to match the complete plotting of all 755 clustered known and new microRNAs (Figure S7). D. Conservation of predicted microRNAs determined using the novel SiPhy metric ω; substitution rate relative to an ancestral repeat-based neutral evolution model (grey dashed line) [45]. Known microRNAs (black) exhibit two distinct peaks with the larger one (left) having relatively high conservation across 29 mammalian species. The 818 miRDeep-predicted microRNAs (red), in contrast, overlapped with the less conserved fraction of the known microRNAs. A small portion of the 146 Ago2 IP selected microRNAs (blue) peaked as a conserved-sequence group but the majority aligned with the non-conserved microRNAs.

If Ago2 IP reliably selected microRNAs among other varieties of small RNA sequences, we expect that these sequences would be reproducibly expressed across biological replicates—in the case of human stem cells, in several independently-isolated cell lines. We quantified sequencing results from small RNA libraries (not immunoprecipitated) from samples of either ESC or NSC staged cultures of H1, HSF1, HSF6, or RG7. Results were adjusted for sequencing depth by converting to counts per million sequences detected (cpm). All 755 Ago2 IP-selected RNAs (146 predicted and 609 known) were detected (File S2). Additionally, the computationally-predicted microRNAs would be expected to be expressed in the stage from which it was originally identified (i.e., ESC or NSC). For the 44 microRNAs predicted based on expression in ESC, 41 were present in at least 3 of the 4 ESC cell lines and only 1 was detected in only a single line. Similarly, from the 116 microRNAs predicted based on NSC expression, 115 were detected in at least 3 of the 4 NSC lines and one was found in only two lines. The measured expression levels clearly distinguish cultures by stage. This was independently observed for all 755 RNAs, only the 609 Ago2 IP-enriched known microRNAs and the 146 Ago2 IP-enriched predicted RNAs (Figure 3A). Examining the distribution of expression levels for known vs. predicted microRNAs in each culture stage, we find that in ESC the predicted microRNAs include lower concentrations than the known populations but the distributions mostly overlap in NSC (Figure S5). This matches our prediction that newly-described microRNAs would be found at lower concentrations in conditions searched previously such as ESC, but that transient developmental stages such as NSC would be a rich source of new microRNAs at any concentration. We conclude that the 146 computationally-predicted microRNAs that are Ago2 IP-enriched are consistently expressed in a biologically-diverse group of samples.

To test for regulation of predicted microRNAs over development, we analyzed expression levels for 755 microRNA (609 known and 146 predicted) that were selected by the Ago2 IP enrichment strategy. RNA was prepared from RG7 hESC cultures at three stages (ESC, NSC, and NRP), induced pluripotent cells obtained from WiCell cultured at two stages (ESC, NSC), and three adult human tissues (brain, heart and kidney). Sequencing libraries were constructed and sequenced (Table S1). Counted observations were normalized by calculating the counts per million beads detected (File S2). In general, microRNA expression levels correlated samples according to their developmental stage (Figure 3B). That is, ESC samples from RG7 hESC were similar to an iPS culture obtained from WiCell. RG7 NSC samples were more like NRP and iPS NSC cultures than other tissues. Similar results were obtained by comparing iPS cultures with other hESC lines (not shown).Three adult tissues were least correlated. This pattern was similar for the Ago2 IP-enriched known microRNAs and for the Ago2 IP-enriched predicted microRNAs, except that the predicted microRNAs grouped brain and iPS NSC samples together. We observed that these iPS cells were more likely to spontaneously differentiate based on culture morphology and that these cells were more likely to produce neural markers upon differentiation (not shown), consistent with the increased correlation of iPS NSC cultures with adult brain with predicted (correlation coefficient of 0.830) but not known microRNAs (0.385, Figure 3B). This is likely due to a more selective expression of newly-identified microRNAs in different tissues or developmental stages.

Results were also K-means clustered to group similar expression patterns for all 755 Ago2 IP-selected microRNAs, using the Pearson correlation of log values as the metric. From the optimal fit of 11 clusters (Figure S6), we observe distinct expression patterns for developmental stages or tissues (Figure 3C). Figure S7 includes all individual expression patterns for all 755 microRNAs analyzed, color-coded by cluster membership. Two clusters (2 and 6) included microRNAs primarily expressed in pluripotent cultures and these clusters included mir-302 family members as expected. Other clusters are primarily specific for adult tissues or for neural precursor cultures. Each cluster included both known and predicted microRNAs. Therefore, the 146 predicted microRNAs selected by Ago2 IP are found to be regulated over differentiation as predicted, but in patterns similar to those of known microRNAs.

Since microRNAs were originally identified by strong evolutionary homology, we surmised that newly-described microRNAs observed in hESC could be less conserved, consistent with a more recent evolutionary appearance, as has been predicted [24]. We employed a novel metric, SiPhy [45], to assess the substitution rate of each new microRNA across a genomic alignment of 29 mammalian species (*manuscript in preparation*). The substitution rates (ω) were determined relative to the substitution rates of ancestral repeats, a model for neutral evolution (Figure 3D). As expected, miRDeep predicted microRNAs (Figure 3D, red) as a class demonstrate higher substitution rates than previously classified ‘known’ microRNAs (Figure 3D, black), suggesting that there is less evolutionary constraint on these sequences. A similar distribution of ω is observed for the subset of new microRNA sequences that were confirmed through direct association with Ago2 (Figure 3D, blue). The new microRNA sequences described here demonstrate a reduced substitution rate from a neutral model (Figure 3D, grey), but are significantly less conserved than the population of known microRNAs. Since previous microRNA annotation efforts have required a high degree of conservation [15], it is clear that the majority of these new sequences would not have met this stringent criterion, despite their physical association with the RISC complex.

## Discussion

By applying ultra-deep sequencing techniques to samples obtained from hESC cultures, we predict up to 818 new microRNA-encoding genes. Chromatin marks associated with these genomic loci are consistent with regulated gene expression. Validation was accomplished by immunoprecipitation of Ago2-containing RISC complexes. These RNAs are drastically regulated over differentiation. Evolutionary conservation among these predicted microRNAs is weaker than for previously identified microRNAs, but we propose that this is appropriate for recently-evolved microRNAs that may be involved in species-specific processes. The fact that 32.9% of the Ago2-selected, newly-described microRNAs shared identical seed sequences with known microRNAs (File S2) supports this conjecture. We believe we have identified a large number of new microRNAs that are expressed in a regulated fashion over development, are associated with RISC complex, and therefore likely function during early stem cell differentiation.

The large number of candidates was likely due to several factors. The use of colorspace coding of nucleic acid sequences allowed us to discard reads that were likely due to technical error during deep sequencing, enhancing the confidence in the remaining sequences. Aligning these sequences to genome with SHRiMP using colorspace retained the advantages of this scheme, ideally enforcing the overlapped interrogation of each nucleotide by two adjacent color codes. We used miRDeep [25] to model microRNA gene structure by comparing both positions and counts of accumulated genome alignments with features characteristic of precursors capable of cleavage by the Microprocessor system. Using the probabilistic scoring provided by miRDeep, we found that a large proportion of our predictions overlapped with scores for known microRNAs (Figure S3). Comparing individual predicted microRNA genes with wiggle tracks of SHRiMP alignments, the general pattern of mature and star strand RNAs coincides with miRDeep-selected results (e.g., Figure 2A). Certainly our conservative selection of only the alignments overlapping at least 10 individual sequence reads limited the production of additional predicted genes. However, we believe that the computational interpretation of deep sequencing reads within a probabilistic model of a Drosha/Dicer substrate generally provided reasonable candidates for further investigation.

Deep sequencing of small RNAs has drawn criticism as some claim that the observed small RNAs are merely degradation fragments of longer RNAs. We uniquely address this critique by selecting for small RNAs that contain the characteristic 5′-phosphate and 3′-hydroxyl groups of RNase III (Dicer) processing through the library construction process. In separate studies, we built SOLiD libraries from RNA samples digested with alkaline phosphatase to destroy 5′-monophosphates and then with Tobacco Acid Phosphatase to convert capped termini to monophosphates. Assessing sample microRNAs using qPCR (not shown) we found that these libraries were enriched for U4 snRNP and depleted for microRNAs, supporting our interpretation that the small RNA libraries used here are not capped. Additionally, in a preliminary alignment to genome, overlapping reads were aggregated together into contiguous intervals. The mean size of these intervals was 21 nt in length for both the H1 ESC and NSC datasets (consistent with the mean size of small RNAs known to be associated with the RNAi machinery) with a mean coverage of ∼150- and ∼200-fold respectively. Furthermore, the largest interval we were able to construct from the overlapping alignments was <170 bases. Approximately 40% of these small RNAs were found to be intragenic to a known transcript (RefSeq and Ensembl), however the majority of these align to intronic regions, with little evidence of exonic, protein-coding sequences aligning to oursmall RNAs. These results, taken together and in conjunction with our other data, suggest a functional role for these sequenced small RNAs within the cell.

A previous study [14] used high-throughput pyrosequencing of concatamers built from small RNAs to detect more than 4×10^5^ sequences—about 240-fold less than the number of sequences we observed. From this starting point, alignments representing mature and “star” sequences were computed into hairpins, similar to steps within the miRDeep algorithm. By these methods, Bar and colleagues identified 14 novel and 53 candidate microRNAs, using expression detection by a second method as validation. Five novel microRNAs were selected for validation. Only one of those, mir-1912, matches a predicted microRNA gene found in our list (chrX:113792287-113792343:+) and this RNA was found to be enriched by Ago2 IP (File S2). We did not test the other, non-overlapping predictions from Bar for Ago2 IP. We conclude that our deeper sequencing allowed the detection of many more predicted microRNA genes. Alternatively, the more sophisticated model used by miRDeep may have increased the likely candidates from existing data.

File S2 lists the locations and sequences for the 146 predicted microRNAs that were found to be enriched by Ago2 IP. A supplemental file (File S5) also provides a standard BED file for visualizing these predicted microRNAs in the genome browser (http://genome.ucsc.edu). Among this group of RNAs, 50 (34%) are found within RefSeq mRNA loci on genome (the enclosing RefSeq names are included in File S2). While many previously-known microRNAs are found clustered in genome, we find that the closest distance between adjacent microRNAs within this new list of 146 is 21 kb, but four of the new microRNAs appear to cluster with previously-known microRNAs (neighbors listed in File S2). While the precise sequence of the mature microRNA that is listed was produced based on probabilistic methods, we note that the calculated seed sequences (positions 2–8) have a 32.9% identity with known microRNAs (p<0.0001, chi-square, see File S2 for identities of shared seeds). For example, predicted microRNA chr16:62121639-62121678:- was based on RNA sequences found in ESC and its expression clusters with mir-302a, with which it shares a seed sequence (cluster 2, see Figure S7). This may represent the evolution of a new family member with slightly different regulatory control based on having different transcriptional regulatory sequences, with different precursor processing based on variation in hairpin sequences, or with target discrimination based on sequence differences outside the seed area. The significantly high proportion of new microRNAs matching seed sequences from previously known microRNAs suggests the presence of new family members, perhaps by a seed-conservative evolutionary mechanism. Certainly for this subset, we predict that cellular mRNA targets must exist since they would be predicted to overlap those of existing microRNAs.

Expression analysis of counted sequencing results reveals a broad range of patterns, as assessed by K-means clustering (Figure 3C and Figure S7). Some clusters are reminiscent of a CNS differentiation pathway, such as clusters 3 and 10. Cluster 3 includes the archetypical nervous system microRNAs mir-9 and mir-124, as well as 12 new microRNAs. We expect that many of these new microRNAs will function in neural differentiation. Two clusters, numbers 2 and 6, contained predominantly pluripotent conditions (hESC and iPS). Interestingly, several individual members of these clusters distinguished embryonic from this example of induced pluripotent cultures. For example, three new microRNAs (chr11:15312537−15312579:+:ESC, chr6:75211867−75211929:+:ESC, and chr8:27799484−27799541:−:ESC, all members of cluster 2) were primarily found in iPS cultures, suggesting that these microRNAs may be induced during reprogramming or perhaps represent a ‘microRNA memory’ of the original cellular phenotype. None of the known microRNA members of this cluster was so specific for iPS cells. In contrast, chr9:123671954−123672027:−:ESC was primarily expressed in hESC instead of iPS, as were mir-371-5p, mir-372, and mir-520a. These differences are consistent with recent studies that suggest cellular reprogramming may be incomplete, and that differences between hESC and iPS cells can be identified at the transcriptional level [46]. Clearly, the combination of expression patterns could be used to distinguish iPS from hESC cultures, but the differences in microRNA expression levels may also contribute to the subtle phenotypic differences observed between iPS and hESC.

Surprisingly, we find that microRNA expression levels are not always predictive of association with Ago2 (Figure S4). This disagrees with a study performed with FLAG-tagged Ago2 in HEK293T cells where no sequence selection was found in loading RISC [47]. We found, for example, that the ES-specific microRNA mir-302 family is over-represented in Ago2 IP samples compared with other microRNAs (Figure S4C). Similarly, the differentiation-specific microRNA mir-145 is over-represented in Ago2 IP samples (Figure S4D). Most new microRNAs are under-represented in both conditions. Perhaps there is a bias in binding Ago2 over other Ago family proteins and this explains the discrepancy? Investigation of the preference of Ago proteins for specific microRNAs found no evidence for such selectivity [43], [44]. The possibility remains that some preference does exist and if we immunoprecipitated all Ago proteins (Ago1-Ago4) we should find an aggregate enrichment proportional to microRNA concentrations in the cell. Alternatively, an unknown cellular mechanism might regulate the association of microRNAs with RISC components so that identifying only those microRNAs found bound with Ago predicts their function. A third possibility is that since our detection methods are so “deep,” we are measuring the Ago-selection of microRNAs at lower concentrations and these rare microRNAs are regulated differently from the constitutive microRNAs that were assayed earlier. A fourth possibility is that the stabilities of microRNAs in RISC are based on finding mRNA targets, so that the most actively targeting microRNAs would be over-represented and those that are present without a currently-expressed target may be under-represented. No matter what the explanation, we conclude that Ago2 binding is a reasonable predictor of function but that lack of binding does not exclude RISC loading by microRNA binding with other Ago family members.

These predicted microRNA genes match most of the generally-accepted definitions for microRNAs [15]. They were identified within a size-fractionated library of cDNAs (here made by PCR instead of cloning) and they are predicted to match a model of fold-back precursor structure and microRNAs biogenesis, as judged within the mirDeep algorithm using RNAfold [25], [40]. At this stage the predicted microRNAs have not been demonstrated to exhibit increased precursor accumulation following Dicer knockdown—this would be technically difficult for a large number of precursors in hESC cultures. While it is possible to obtain transient Dicer knockdown using siRNAs (not shown), selection of stably transfected hESC lines often leads to differentiation, altering microRNA expression patterns. Finally, the requirement for phylogenetic conservation cannot be demonstrated for the majority of these microRNAs since we hypothesize that they are recently evolved (Figure 3D). However, within the established criteria, one mode of acceptance is strong evidence of expression and the establishment of a hairpin structure on the precursor. These two criteria were tested here. Sequencing detected both mature and star strands of a computed hairpin. Furthermore, we conservatively filtered the predictions for expression in multiple cell lines in multiple differentiation stages. We also tested predictions for co-immunoprecipitation with Ago2, an indication of the presence in a RISC ribonucleoprotein complex. The inclusion of this final validation step, we believe, provides valuable proof that at least these predicted microRNAs found associated with Ago2 should be acceptable as microRNAs.

Association with Ago2 alone is not sufficient to define a microRNA. For example, recent studies found Ago2 bound with dsRNAs associated with promoter elements [48], Dicer cleavage products of snoRNAs [49], or mitochondrial tRNA [50]. However, our screening strategy specifically identified Drosha-like substrate structures in genomic DNA and subtracted other classes of known small RNAs including tRNAs and snoRNAs, supporting our conclusion that we have identified new microRNAs. One way to substantiate this claim is to demonstrate cleavage of predicted precursors by Microprocessor complex by inhibiting the RNA binding protein DGCR8, as has been done in mouse ES cells [6]. We attempted this by transient transfection of DGCR8-specific siRNAs into hESC cultures. While this technique was successful at reducing DGCR8 mRNA, by 3 days after transfection it did not alter levels of DGCR8 protein, as judged by Westerns or of known microRNAs, as judged by qPCR (not shown). Creating stably transfected lines from hESC is difficult since the cells tend to differentiate upon long-term selection. We will continue to search for appropriate methods to demonstrate that the predicted precursors are processed by microRNA pathways.

The discovery of new microRNAs in hESC and their differentiating cellular products provides the basis for a complex regulatory network. Regulated expression of microRNAs upon differentiation satisfies the hypothesized role of microRNAs in canalization, or stabilization of nascent cell types in the face of stochastic regulatory drift. An elegant hypothesis was recently published considering the evolutionary pressures on microRNAs and how they may function to stabilize species identities or even cell types from one another [51]. These authors conjecture that microRNAs have dual functions: gene expression tuning and expression buffering, and that both of these contribute to stabilizing homeostasis. Furthermore, the action of microRNAs on translation may be modest—few examples show more than 50% change in target protein levels [52]. Wu and colleagues consider that microRNAs, as agents of stabilization, may only be phenotypically functional when homeostasis is perturbed [51]. In the context of stem cell differentiation, this may be just the case—that the perturbation of homeostasis from pluripotency to differentiation programs is exactly the situation when the function of microRNAs is most important. The expanded number of microRNAs found in this study and associated with RISC provides a broad collection of molecules poised to reinforce the dynamic stability of cell type-specific phenotypes during early hESC development.

## Supporting Information

Methods S1 (0.26 MB DOC)

Table S1 Summary of SOLiD experiments. For each of the experiments used in this study, the table shows the samples run, the number of usable sequences (“good and best” classification by SOLiD software) and the number of sequences that were unique. As an example, for slide 1 there were a total of 83,644,650 and 80,542,053 beads read for ESC and NSC samples, respectively. The number of usable beads was, then, 55.2% and 79.2% of the total beads detected. The percentage of usable sequences was largely reflective of the density that beads were loaded onto the slides with higher densities leading to a greater number of total beads but a lower percentage of usable sequences.
(0.04 MB DOC)

Figure S1 Digital gene expression analysis of known microRNAs in SOLiD sequencing datasets. For this analysis perfect string matching was used to identify and count known human microRNAs (miRBase v. 11). Resulting counts were normalized by total sequences for each sample, deriving the “cpm” or counts per million sequences. A hierarchical cluster was drawn of microRNAs explaining the difference between ESC and neural precursors (Student's t-test, 5% FDR, ≥1.5-fold). The dendrogram showing association between samples, however, was calculated from all microRNAs using correlation as the metric.
(1.18 MB EPS)

Figure S2 Distributions of alignments and predictions by chromosome. In the top panel, all 591 million alignments are plotted by chromosome using the number of alignments per million bases (MB). The middle panel shows the total number of miRDeep predictions, for each differentiation stage, by chromosome. At bottom are the predictions after filtering out known microRNAs, RNA genes, and repeat sequences (See Fig. 2B).
(0.99 MB EPS)

Figure S3 Log-odds scores produced by miRDeep for known and predicted microRNAs. Novel microRNAs predicted by miRDeep (solid lines) tended to have lower scores than known microRNAs (dashed lines) but a large fraction overlapped.
(0.80 MB EPS)

Figure S4 Relationship between sequence counts observed in unfractionated or Ago2 IP-selected samples. For panels A and B, normalized log counts of sequences found to be Ago2 IP-enriched were calculated and displayed for measures of direct expression (RG7 ESC) vs. Ago2 IP samples (Ago2 IP RG7 ESC). Known microRNAs are depicted as black squares and predicted microRNAs are depicted as blue rectangles. Panel A is from ESC and panel B is from NSC. For each case, linear regression was calculated based on known microRNAs and used to predict Ago2 IP counts. For ESC, the r^2^ is 0.697 and for NSC the r^2^ is 0.109 (p<0.001 for each case). The top 20 outliers, as determined by the greatest residuals, are shown in panels C (ESC) and D (NSC). Most predicted microRNAs are under-represented by these calculations but several known microRNAs are among the top lists of over- or under-represented sequences, demonstrating differences in comparing expression and Ago2 binding.
(1.56 MB EPS)

Figure S5 Distributions of expression levels for known and predicted microRNAs, split by developmental stage. Mean expression levels from four cell lines (H1, HSF1, HSF6, and RG7) at two stages (ESC, NSC) were calculated. The blue line shows the distribution of 609 known microRNAs and the black line shows the 146 predicted microRNAs selected by Ago2 IP. Results show that the novel microRNAs in ESC exhibit a lower range of expression levels, as predicted. Furthermore, the range of novel microRNA expression in NSC was similar to that of known microRNAs, agreeing with the hypothesis that unknown microRNAs could be found in transient stages of differentiation.
(1.16 MB EPS)

Figure S6 K-means best fit plot for expression analysis shown in Figures 3 and S7. By judging the best fit as the minimum mean sum of squares at k = 11, we selected 11 clusters for the dataset.
(0.84 MB EPS)

Figure S7 Individual expression plots for all 755 known and predicted microRNAs. Colors of plots match the cluster means plotted in Fig. 3C to identify cluster numbers. Expression levels are calculated as cpm, or counts per million sequences.
(1.31 MB PDF)

File S1 Excel file containing TaqMan microRNA Array results for RG7 hESC stages. A single sample of RG7 ESC, NSC, or NPC culture RNA (the same samples used for the Illumina Beadchip microarray assay) were assessed by qPCR for known microRNAs using the Applied Biosystems TaqMan Human microRNA array cards (A and B, part numbers 4398965 and 4398966), following the manufacturer's recommended protocol. For each probe, the “−dC_t_” or negative delta C_t_ (cycle threshold) is shown, subtracting the C_t_ value for U6 snRNA endogenous control (not shown). To calculate quantities relative to ESC, the negative delta-delta C_t_ (−ddC_t_) was calculated by subtracting the dCt for ESC, and then the relative quantity (RQ, labeled here as 2̂−ddCt) was calculated by making this value the exponent of power 2.
(0.27 MB XLS)

File S2 Excel file containing all microRNA predictions and expression levels. Contents of worksheets: 1. H1 predicted microRNAs: the list of 818 predicted microRNAs filtered as described in Figure 2B. This sheet matches the BED file in Supplemental File 2. 2. Ago2 IP novel: the 146 predicted microRNAs that were found to be enriched following Ago2 IP compared with IgG IP. The mean enrichment is shown for each stage (ESC and NSC). Also listed are the mature sequence of the predicted microRNA, the calculated seed (positions 2–8), a hyperlink to the TargetScan web site to search for targets, and the known human microRNAs matching the seed sequence. 3. Ago2 IP known: This sheet lists the 609 known microRNAs found to be enriched by Ago2 IP over IgG IP (see mean enrichment values). 4. hESC lines: The calculated counts per million expression levels in four human stem cell lines at two differentiation stages, used in creating Figure 3A. 5. Clustered expression: The calculated counts per million expression levels for all 609 known and 146 predicted microRNAs in three stages of hESC (ESC, NSC, and NRP), two iPS cultures (ESC and NSC, obtained from WiCell), and three adult tissues (brain, heart, kidney). Also listed are the cluster numbers matching Figures 3C, S6, and S7).
(0.55 MB XLS)

File S3 qPCR assay of Ago2 immunoprecipitated samples. Predicted microRNAs were assayed in small RNA SREK libraries by SYBR green qPCR (SYBR Green Master Mix, Applied Biosystems) using one microRNA-specific primer and one primer specific for the library (5′-CTC CTG TAC GGC CAA GGC G-3′). Custom microRNA probes were designed by synthesizing DNA oligos identical to the probed RNAs. Results show the C_t_ value (mean of two technical replicates) for each RNA. The “delC_t_” was calculated by subtracting the detected C_t_ for the spiked-in NCode control small RNA (see text). If qPCR product was not observed for a probe (blank entry in C_t_ column), 40 cycles was substituted to calculate the minimum observable quantity of target. The “deldelC_t_” subtracts the delC_t_ for IgG background from the Ago2 delC_t_, and the resulting value is used to calculate relative quantity (RQ) by raising 2 to the power of the deldelC_t_. In this case, RQ refers to the relative enrichment of the target RNA by Ago2 IP over background IgG IP.
(0.05 MB XLS)

File S4 BED file for UCSC genome browser listing human genome (hg18) coordinates of 818 predicted microRNAs identified by miRDeep based on SOLiD sequenced small RNAs aligned with SHRiMP.
(0.02 MB ZIP)

File S5 BED file for UCSC genome browser listing genomic coordinates of 146 predicted microRNAs found to be enriched by Ago2 IP.
(0.00 MB ZIP)
