# Supplementary material for: Ago2 Immunoprecipitation Identifies Predicted MicroRNAs in Human Embryonic Stem Cells and Neural Precursors
Source: PLoS One. 2009 Sep 28;4(9):e7192. doi: 10.1371/journal.pone.0007192 (PMC2745660; doi:10.1371/journal.pone.0007192)
Supplement: Methods S1 — (0.26 MB DOC) [file pone.0007192.s001.doc]

**Supplemental Methods S1**

Reshaping SHRiMP output for miRDeep analysis


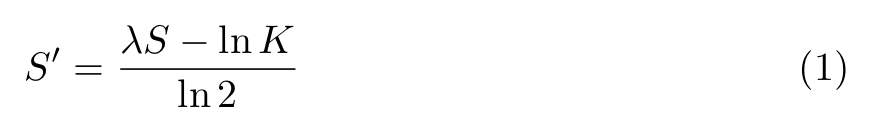

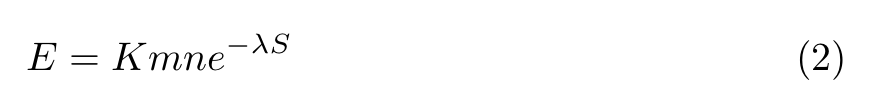
The raw output from the SHRiMP output was reshaped using a custom python pipeline. The miRDeep pipeline is designed to work on raw BLAST outputs to generate an intermediate, tab-delimited ‘blastparse’ format. The SHRiMP algorithm, used here to align our raw read sequences to the genome in colorspace, does not directly provide the required bitscore and e-value fields so these were calculated for each alignment and additional information was aggregated to mirror the miRDeep ‘blastparse’ format. Briefly, raw local alignment scores were converted to bitscores using equation (1). The natural scales for the search space, K and λ, were pre-computed for version hg18 of the human genome. E-values for each alignment to genome were computed using equation (2). Alignments were segregated by chromosome and strand and were used as input for the miRDeep algorithm respectively. Initially, putative precursor sequences were obtained by sliding 100nt windows across each SHRiMP alignment and associated flanking sequences. Each putative precursor was folded using RNAfold (Gruber, et al., 2008). Original colorspace reads were then translated to DNA space and aligned to the putative precursors via BLAST. Putative precursors, fold structures, and read alignments to precursors for each chromosome were then input into miRDeep and fit to a probabilistic model for microRNA structure. A log-odds miRDeep score cutoff of 1 was used as a preliminary filter to yield 1,216 predictions in ESC, and 4,494 in NSC.

Ago2 Immunoprecipitation

Anti-human Ago2 (11A9, shipped as tissue culture supernatant; Ascenion GmbH, Helmholtz Zentrum, München) was tested for antigen specificity by Western blotting, which produced a single band of approximately 100 kDa (not shown). To prepare antibody-coated beads, 60 μl of a protein-G Sepharose bead slurry (ZYMED) was incubated with either 750 μl of anti-human Ago2 or 5 μg of rat IgG (Millipore, PP68) for 2 hrs at 4°C. To prepare immunoprecipitates, approximately 15x10^6^ cells were lysed in lysis buffer (20 mM Tris-HCl pH 7.5, 150 mM NaCl, 0.5% Nonidet P-40, 2 mM EDTA, 0.5 mM DTT, 1 mM NaF, 1X Halt protease inhibitor [Pierce], 10 U/ml RNAse Out [Invitrogen]) for 10 min on ice. Lysates were cleared by centrifugation (16,000 x g) for 10 min at 4°C. Cleared lysates were then incubated with the coated Sepharose beads for 90 mins at 4°C on a rocker. Beads were washed twice with lysis buffer for 10 min at 4°C and once more with 1x PBS. Bound material was eluted from beads with 50 μl of 0.1 M glycine (pH 2.3) for 15 min at room temperature. Eluted fractions were neutralized immediately with an equal volume of 1 M Tris-HCl (pH 8) and then treated with 20 U of proteinase K for 10 min at 65°C. Prior to RNA extraction, samples were spiked with 25 pmol of a synthetic microRNA (NCode Control RNA, Invitrogen, 5’- UGUGUCCGGAGCAAUGACAG’-3’) to provide an endogenous control to correct for differences in library preparation or sequencing depth. RNA was prepared using Trizol reagent and used to construct SOLiD libraries with the SREK kit.

Selection of Ago2 IP-enriched sequences

RNA samples derived from immunoprecipitates were spiked with a known quantity of synthetic alien microRNA (NCode control microRNA, Invitrogen). Counts of sequences were normalized to the total number of sequences in the sample and then to the alien microRNA counts. Fold-enrichment was calculated for Ago2 IP compared with IgG control. To select a cut-off value we attempted to use a p-value based on a binomial distribution. Briefly, normalized counts were adjusted to reflect numbers of observations, and then a linear regression was used to calculate “expected” values. A p-value was selected by fitting the normalized counts to the cumulative density function of the binomial distribution where *k* = normalized IgG counts and *n* = *k* + normalized Ago2 counts, with a probability of p=0.5. At p<0.05, 361 known microRNAs were selected among 848 detected possibilities. A substantial group of known microRNAs was found to be near this p-value cut-off, and these could be included by expanding our filtering to a simple 4-fold enrichment cut-off, so we chose to extend our filtering to produce a more complete list.

Bioinformatics methods

Partitioning multivariate data with the k-means algorithm was performed using the Kmeans() function from the amap library in R. In each case, the distance metric is described. The optimal number of clusters was determined by finding the minimum mean sum of squares as an aggregate measure of fitting the data to the k-means.

BLAST matching of microRNA sequences to sequence records was performed using the command-line blastall program, setting the expect value to 1 (to be stringent when matching the short lengths of microRNA sequences) and using the blastn nucleotide program. The purpose in using BLAST was to allow minimal mismatch in counting sequences. This process was parallelized on a ROCKS cluster using a custom Python script to submit jobs to the SGE scheduler. Results were collected and aggregated using another Python script combined with the pivot tables function in Excel.
