# Supplementary material for: Ago2 Immunoprecipitation Identifies Predicted MicroRNAs in Human Embryonic Stem Cells and Neural Precursors
Source: PLoS One. 2009 Sep 28;4(9):e7192. doi: 10.1371/journal.pone.0007192 (PMC2745660; doi:10.1371/journal.pone.0007192)
Supplement: Table S1 — Summary of SOLiD experiments. For each of the experiments used in this study, the table shows the samples run, the number of usable sequences (“good and best” classification by SOLiD software) and the number of sequences that were unique. As an example, for slide 1 there were a total of 83,644,650 and 80,542,053 beads read for ESC and NSC samples, respectively. The number of usable beads was, then, 55.2% and 79.2% of the total beads detected. The percentage of usable sequences was largely reflective of the density that beads were loaded onto the slides with higher densities leading to a greater number of total beads but a lower percentage of usable sequences. (0.04 MB DOC) [file pone.0007192.s002.doc]

Table S1

| **Expt** | **Cell Line** | **Stage** | **Usable Sequences** | **Unique** |
| --- | --- | --- | --- | --- |
| 1 | H1 | ESC | 46,206,912 | 31,914,055 |
|  | H1 | NSC | 63,776,546 | 38,005,054 |
| 2 | H1 | EB | 3,113,116 | 1,396,200 |
|  | H1 | ESC | 4,792,431 | 1,662,169 |
|  | H1 | NSC | 11,656,630 | 4,815,116 |
|  | HSF1 | ESC | 14,267,249 | 4,466,092 |
|  | HSF1 | NPC | 8,686,859 | 6,817,385 |
|  | HSF6 | ESC | 5,174,676 | 1,675,208 |
|  | HSF6 | NPC | 5,398,018 | 3,325,665 |
| 3 | RG7 | NP | 58,295,712 | 38,831,416 |
|  | RG7 | NSC | 36,668,964 | 18,664,544 |
|  | RG7 | ESC | 43,415,595 | 26,446,630 |
|  | Adult | Heart | 35,633,467 | 18,026,698 |
| 4 | Adult | Brain | 25,311,346 | 10,497,242 |
|  | Adult | Kidney | 16,325,661 | 6,653,318 |
|  | RG7 | ESC IgG | 27,989,174 | 15,532,080 |
|  | RG7 | ESC Ago2 | 24,911,304 | 16,428,771 |
|  | RG7 | NSC IgG | 22,438,907 | 14,842,992 |
|  | RG7 | NSC Ago2 | 20,496,616 | 11,692,024 |
|  | iPS | ESC | 17,151,040 | 9,864,271 |
|  | iPS | NSC | 20,000,830 | 9,694,512 |
