# Supplementary material for: Ago2 Immunoprecipitation Identifies Predicted MicroRNAs in Human Embryonic Stem Cells and Neural Precursors
Source: PLoS One. 2009 Sep 28;4(9):e7192. doi: 10.1371/journal.pone.0007192 (PMC2745660; doi:10.1371/journal.pone.0007192)
Supplement: Figure S7 — Individual expression plots for all 755 known and predicted microRNAs. Colors of plots match the cluster means plotted in Fig. 3C to identify cluster numbers. Expression levels are calculated as cpm, or counts per million sequences. (1.31 MB PDF) [file pone.0007192.s009.pdf]

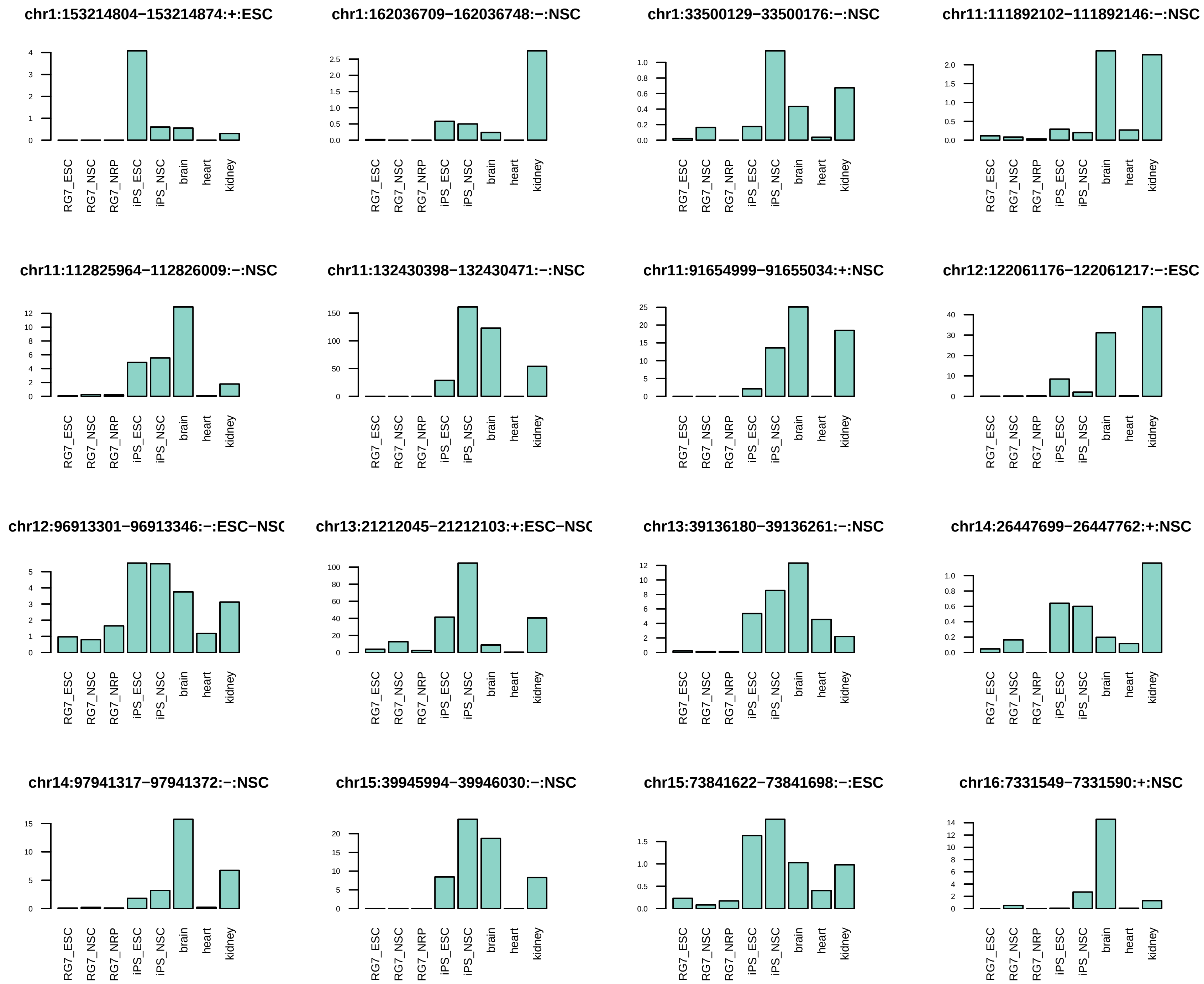
chr1:153214804−153214874:+:ESC
chr1:162036709−162036748:−:NSC
chr1:33500129−33500176:−:NSC
chr11:111892102−111892146:−:NSC
chr11:112825964−112826009:−:NSC
chr11:132430398−132430471:−:NSC
chr11:91654999−91655034:+:NSC
chr12:122061176−122061217:−:ESC
chr12:96913301−96913346:−:ESC−NSC
chr13:21212045−21212103:+:ESC−NSC
chr13:39136180−39136261:−:NSC
chr14:26447699−26447762:+:NSC
chr14:97941317−97941372:−:NSC
chr15:39945994−39946030:−:NSC
chr15:73841622−73841698:−:ESC
chr16:7331549−7331590:+:NSC
RG7_ESC
RG7_NSC
RG7_NRP
iPS_ESC
iPS_NSC
brain
heart
kidney

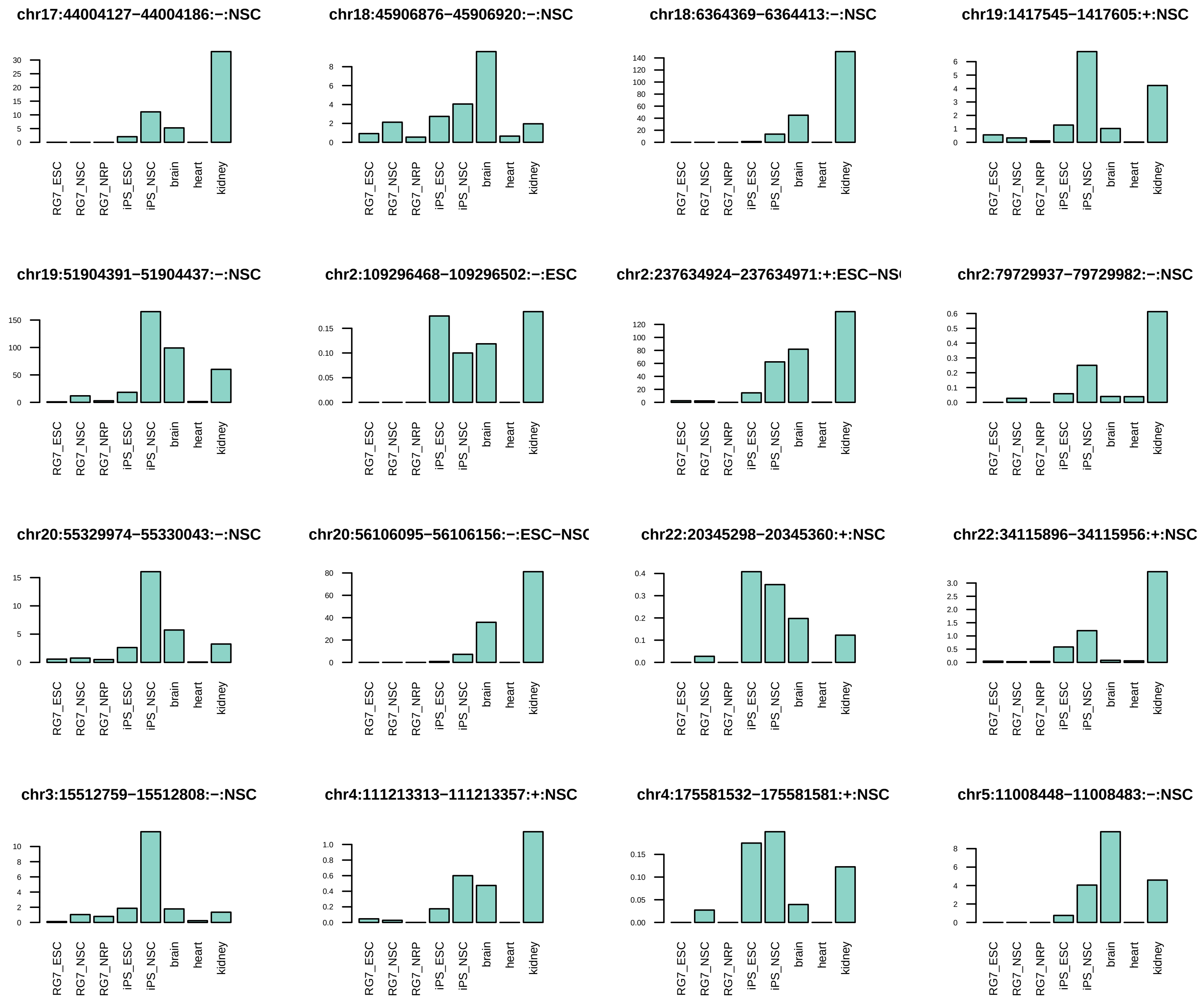

chr17:44004127−44004186:−:NSC
chr18:45906876−45906920:−:NSC
chr18:6364369−6364413:−:NSC
chr19:1417545−1417605:+:NSC
chr19:51904391−51904437:−:NSC
chr2:109296468−109296502:−:ESC
chr2:237634924−237634971:+:ESC−NS
chr2:79729937−79729982:−:NSC
chr20:55329974−55330043:−:NSC
chr20:56106095−56106156:−:ESC−NSC
chr22:20345298−20345360:+:NSC
chr22:34115896−34115956:+:NSC
chr3:15512759−15512808:−:NSC
chr4:111213313−111213357:+:NSC
chr4:175581532−175581581:+:NSC
chr5:11008448−11008483:−:NSC
RG7_ESC
RG7_NSC
RG7_NRP
iPS_ESC
iPS_NSC
brain
heart
kidney

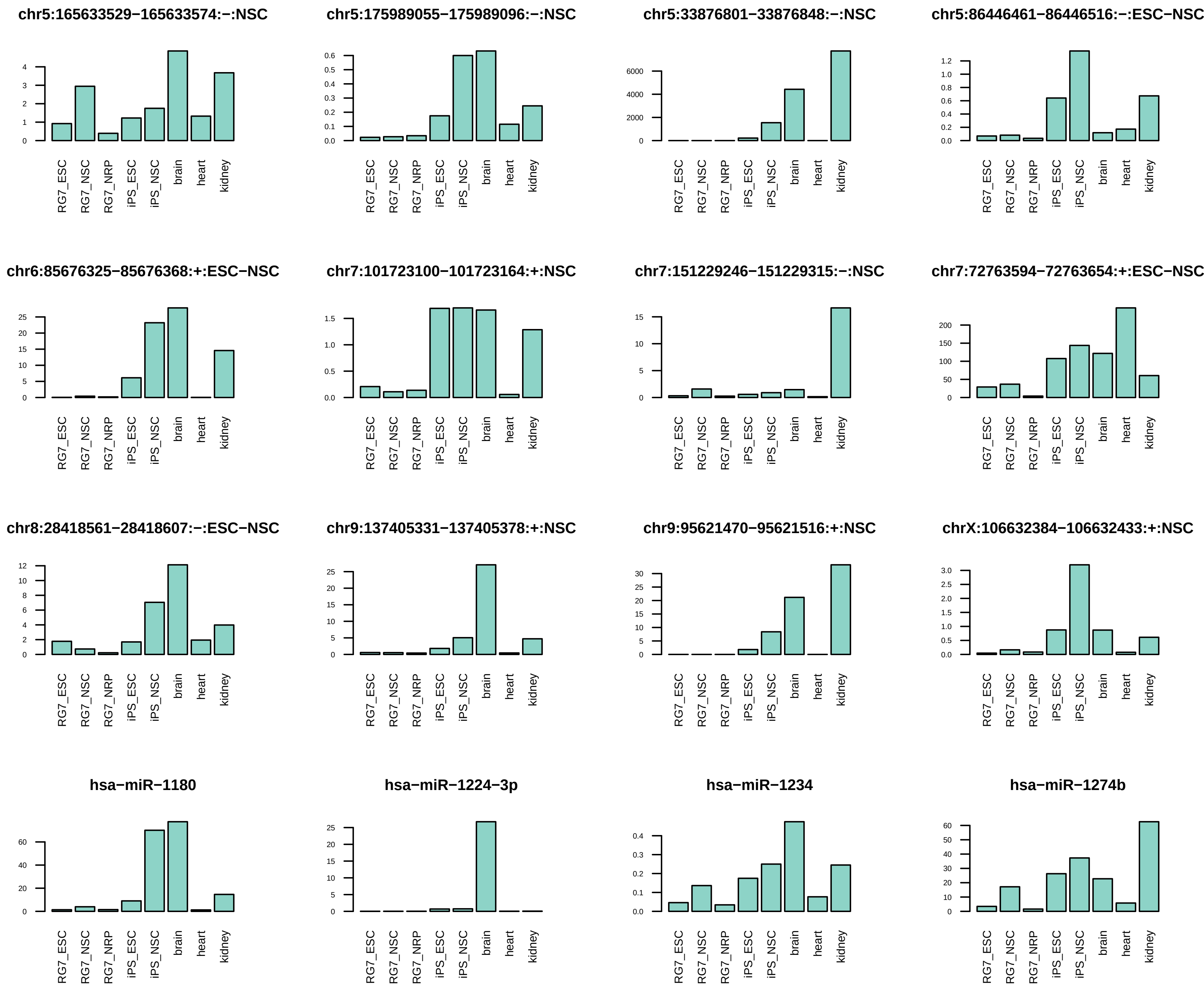
chr5:165633529−165633574:−:NSC
0 1 2 3 4
chr5:175989055−175989096:−:NSC
0.0 0.1 0.2 0.3 0.4 0.5 0.6
chr5:33876801−33876848:−:NSC
0 2000 4000 6000
chr5:86446461−86446516:−:ESC−NSC
0.0 0.2 0.4 0.6 0.8 1.0 1.2
chr6:85676325−85676368:+:ESC−NSC
0 5 10 15 20 25
chr7:101723100−101723164:+:NSC
0.0 0.5 1.0 1.5
chr7:151229246−151229315:−:NSC
0 5 10 15
chr7:72763594−72763654:+:ESC−NSC
0 50 100 150 200
chr8:28418561−28418607:−:ESC−NSC
0 2 4 6 8 10 12
chr9:137405331−137405378:+:NSC
0 5 10 15 20 25
chr9:95621470−95621516:+:NSC
0 5 10 15 20 25 30
chrX:106632384−106632433:+:NSC
0.0 0.5 1.0 1.5 2.0 2.5 3.0
hsa−miR−1180
0 20 40 60
hsa−miR−1224−3p
0 5 10 15 20 25
hsa−miR−1234
0.0 0.1 0.2 0.3 0.4
hsa−miR−1274b
0 10 20 30 40 50 60
RG7_ESC
RG7_NSC
RG7_NRP
iPS_ESC
iPS_NSC
brain
heart
kidney

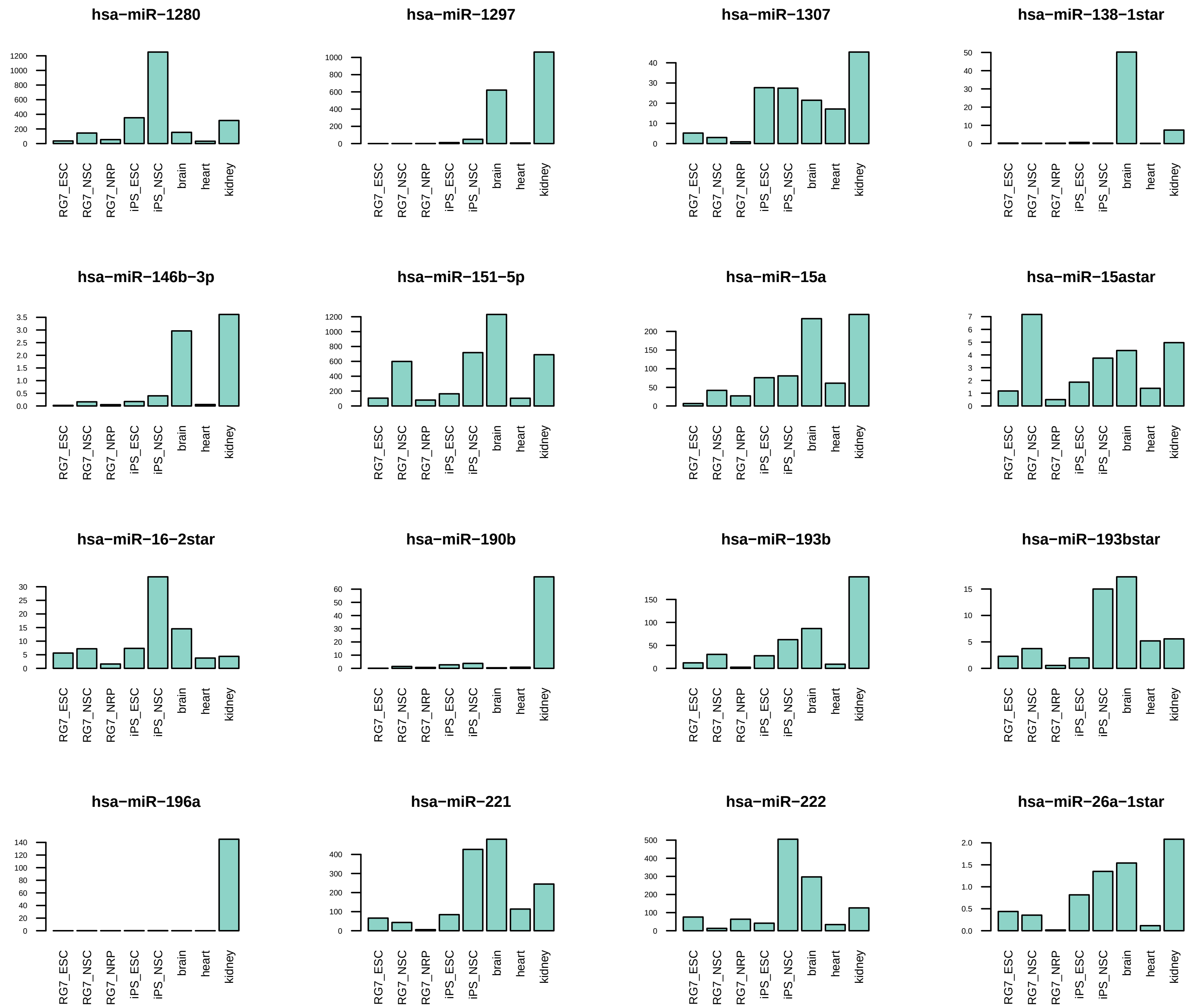

hsa−miR−1280
0
200
400
600
800
1000
1200
RG7_ESC
RG7_NSC
RG7_NRP
iPS_ESC
iPS_NSC
brain
heart
kidney
hsa−miR−1297
0
200
400
600
800
1000
hsa−miR−1307
0
10
20
30
40
hsa−miR−138−1star
0
10
20
30
40
50
hsa−miR−146b−3p
0.0
0.5
1.0
1.5
2.0
2.5
3.0
3.5
hsa−miR−151−5p
0
200
400
600
800
1000
1200
hsa−miR−15a
0
50
100
150
200
hsa−miR−15astar
0
1
2
3
4
5
6
7
hsa−miR−16−2star
0
5
10
15
20
25
30
hsa−miR−190b
0
10
20
30
40
50
60
hsa−miR−193b
0
50
100
150
hsa−miR−193bstar
0
5
10
15
hsa−miR−196a
0
20
40
60
80
100
120
140
hsa−miR−221
0
100
200
300
400
hsa−miR−222
0
100
200
300
400
500
hsa−miR−26a−1star
0.0
0.5
1.0
1.5
2.0

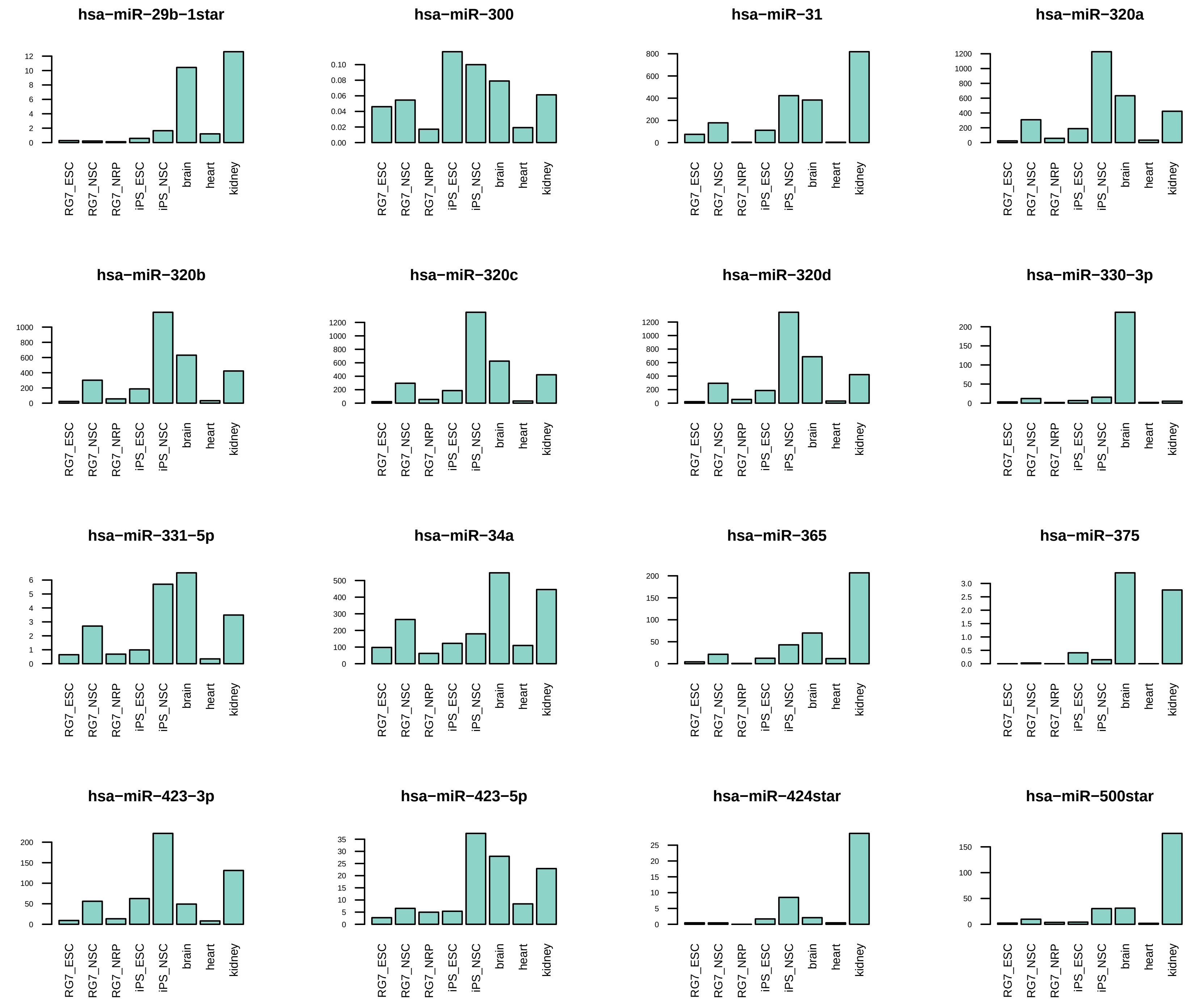

hsa−miR−29b−1star
0 2 4 6 8 10 12
hsa−miR−300
0.00 0.02 0.04 0.06 0.08 0.10
hsa−miR−31
0 200 400 600 800
hsa−miR−320a
0 200 400 600 800 1000 1200
hsa−miR−320b
0 200 400 600 800 1000
hsa−miR−320c
0 200 400 600 800 1000 1200
hsa−miR−320d
0 200 400 600 800 1000 1200
hsa−miR−330−3p
0 50 100 150 200
hsa−miR−331−5p
0 1 2 3 4 5 6
hsa−miR−34a
0 100 200 300 400 500
hsa−miR−365
0 50 100 150 200
hsa−miR−375
0.0 0.5 1.0 1.5 2.0 2.5 3.0
hsa−miR−423−3p
0 50 100 150 200
hsa−miR−423−5p
0 5 10 15 20 25 30 35
hsa−miR−424star
0 5 10 15 20 25
hsa−miR−500star
0 50 100 150
RG7_ESC
RG7_NSC
RG7_NRP
iPS_ESC
iPS_NSC
brain
heart
kidney

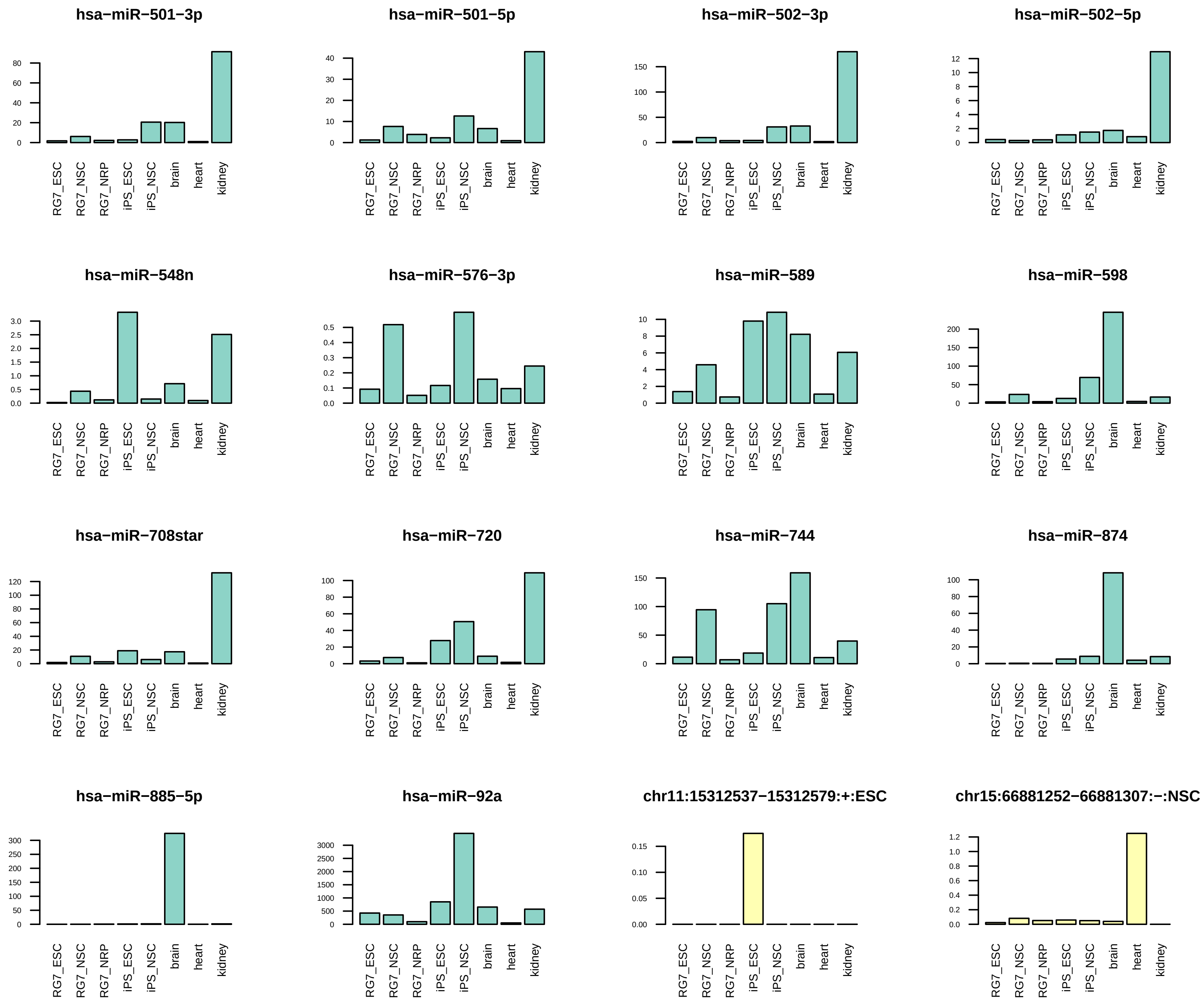
hsa−miR−501−3p
hsa−miR−501−5p
hsa−miR−502−3p
hsa−miR−502−5p
hsa−miR−548n
hsa−miR−576−3p
hsa−miR−589
hsa−miR−598
hsa−miR−708star
hsa−miR−720
hsa−miR−744
hsa−miR−874
hsa−miR−885−5p
hsa−miR−92a
chr11:15312537−15312579:+:ESC
chr15:66881252−66881307:−:NSC
RG7_ESC
RG7_NSC
RG7_NRP
iPS_ESC
iPS_NSC
brain
heart
kidney

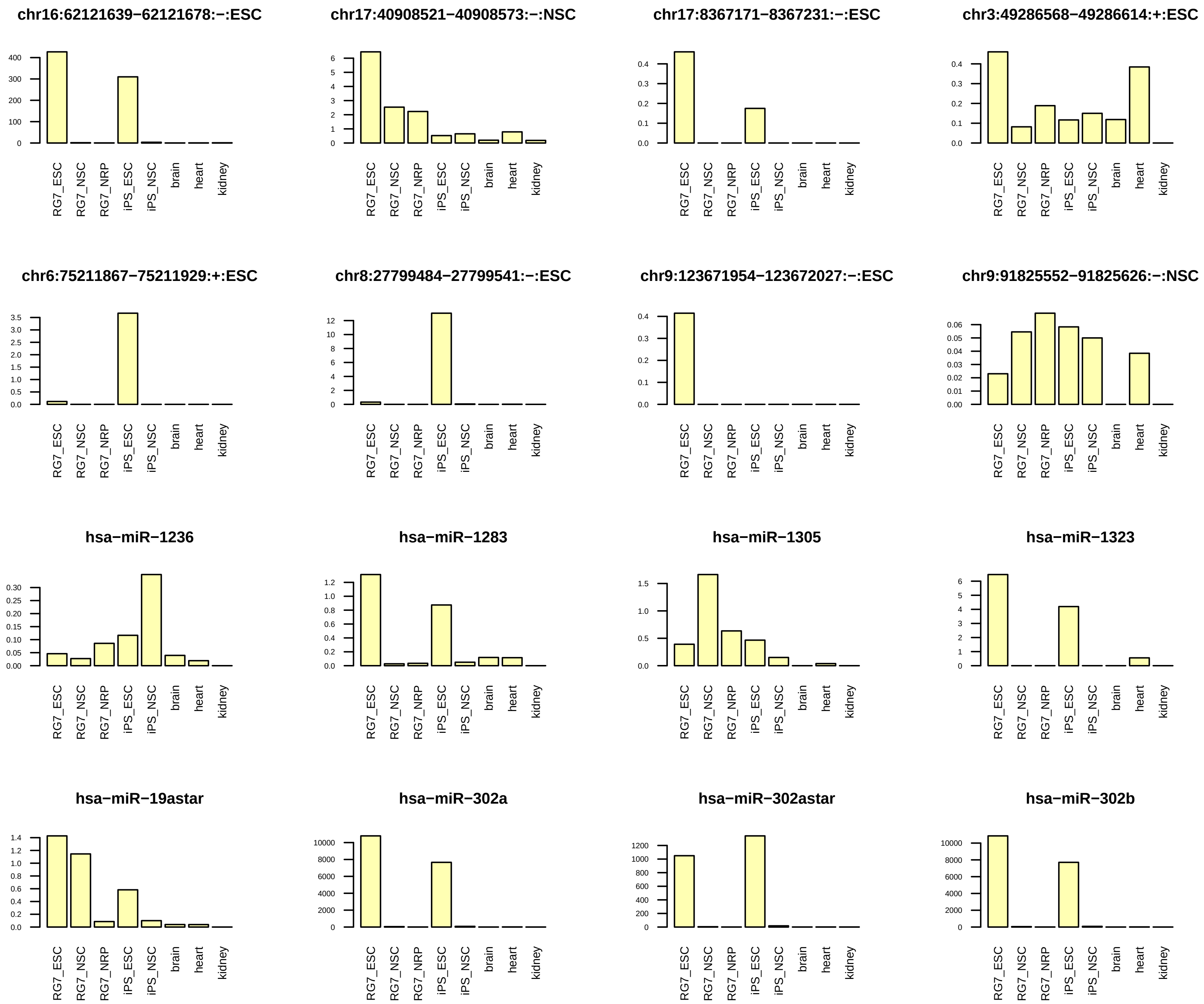
chr16:62121639−62121678:−:ESC
0
100
200
300
400
chr17:40908521−40908573:−:NSC
0
1
2
3
4
5
6
chr17:8367171−8367231:−:ESC
0.0
0.1
0.2
0.3
0.4
chr3:49286568−49286614:+:ESC
0.0
0.1
0.2
0.3
0.4
chr6:75211867−75211929:+:ESC
0.0
0.5
1.0
1.5
2.0
2.5
3.0
3.5
chr8:27799484−27799541:−:ESC
0
2
4
6
8
10
12
chr9:123671954−123672027:−:ESC
0.0
0.1
0.2
0.3
0.4
chr9:91825552−91825626:−:NSC
0.00
0.01
0.02
0.03
0.04
0.05
0.06
hsa−miR−1236
0.00
0.05
0.10
0.15
0.20
0.25
0.30
hsa−miR−1283
0.0
0.2
0.4
0.6
0.8
1.0
1.2
hsa−miR−1305
0.0
0.5
1.0
1.5
hsa−miR−1323
0
1
2
3
4
5
6
hsa−miR−19astar
0.0
0.2
0.4
0.6
0.8
1.0
1.2
1.4
hsa−miR−302a
0
2000
4000
6000
8000
10000
hsa−miR−302astar
0
200
400
600
800
1000
1200
hsa−miR−302b
0
2000
4000
6000
8000
10000
RG7_ESC
RG7_NSC
RG7_NRP
iPS_ESC
iPS_NSC
brain
heart
kidney

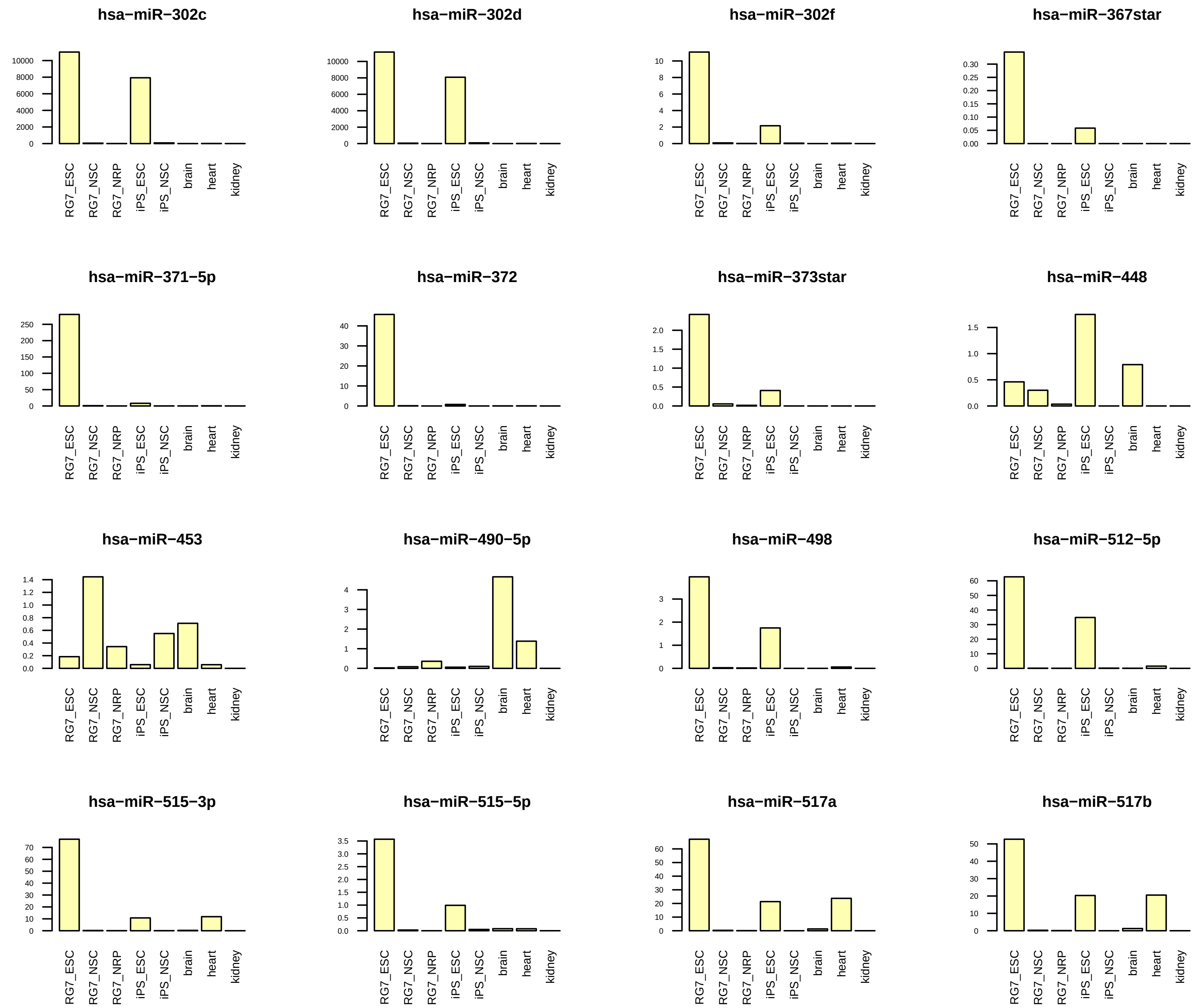
hsa−miR−302c
0
2000
4000
6000
8000
10000
hsa−miR−302d
0
2000
4000
6000
8000
10000
hsa−miR−302f
0
2
4
6
8
10
hsa−miR−367star
0.00
0.05
0.10
0.15
0.20
0.25
0.30
hsa−miR−371−5p
0
50
100
150
200
250
hsa−miR−372
0
10
20
30
40
hsa−miR−373star
0.0
0.5
1.0
1.5
2.0
hsa−miR−448
0.0
0.5
1.0
1.5
hsa−miR−453
0.0
0.2
0.4
0.6
0.8
1.0
1.2
1.4
hsa−miR−490−5p
0
1
2
3
4
hsa−miR−498
0
1
2
3
hsa−miR−512−5p
0
10
20
30
40
50
60
hsa−miR−515−3p
0
10
20
30
40
50
60
70
hsa−miR−515−5p
0.0
0.5
1.0
1.5
2.0
2.5
3.0
3.5
hsa−miR−517a
0
10
20
30
40
50
60
hsa−miR−517b
0
10
20
30
40
50
RG7_ESC
RG7_NSC
RG7_NRP
iPS_ESC
iPS_NSC
brain
heart
kidney

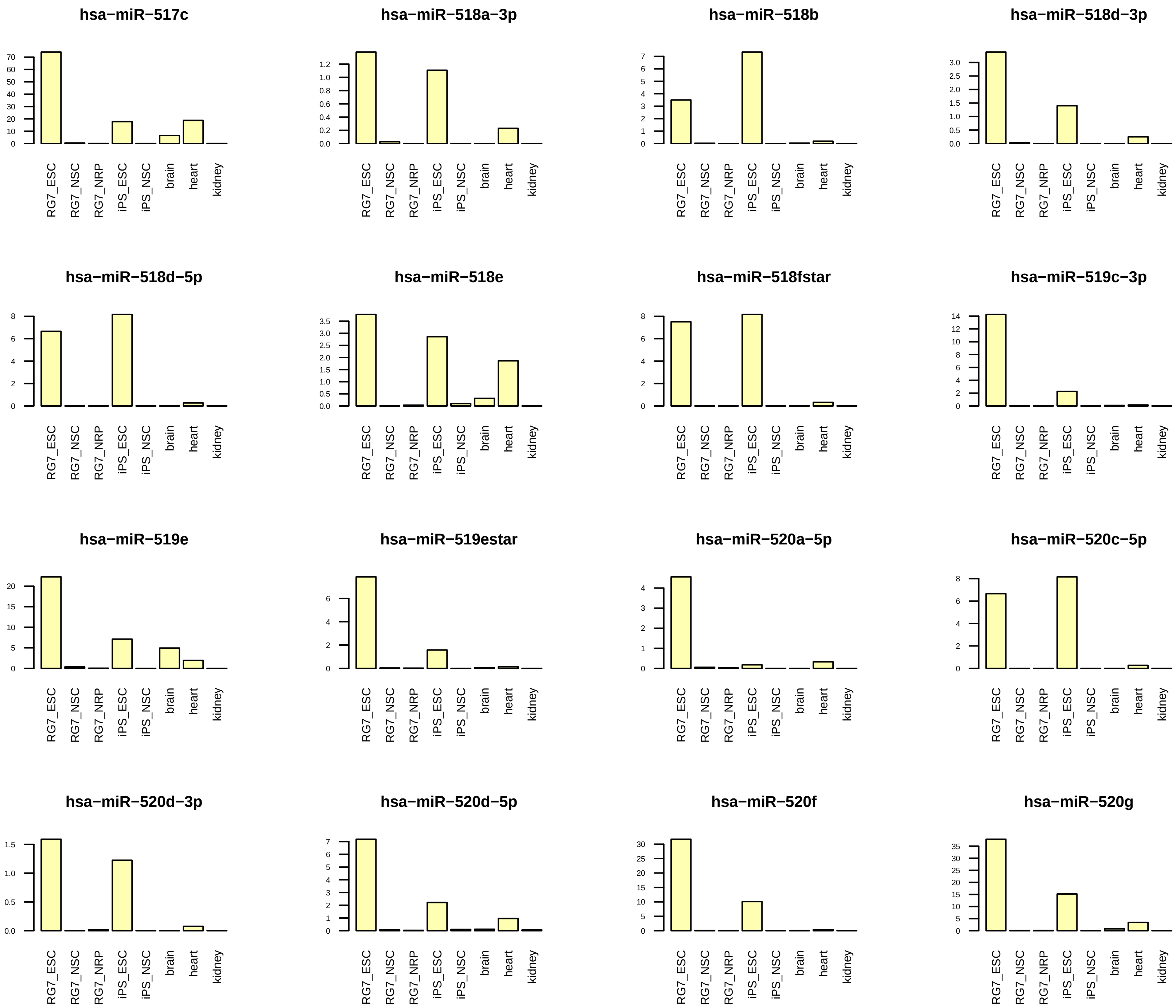
hsa−miR−517c
hsa−miR−518a−3p
hsa−miR−518b
hsa−miR−518d−3p
hsa−miR−518d−5p
hsa−miR−518e
hsa−miR−518fstar
hsa−miR−519c−3p
hsa−miR−519e
hsa−miR−519estar
hsa−miR−520a−5p
hsa−miR−520c−5p
hsa−miR−520d−3p
hsa−miR−520d−5p
hsa−miR−520f
hsa−miR−520g
RG7_ESC
RG7_NSC
RG7_NRP
iPS_ESC
iPS_NSC
brain
heart
kidney

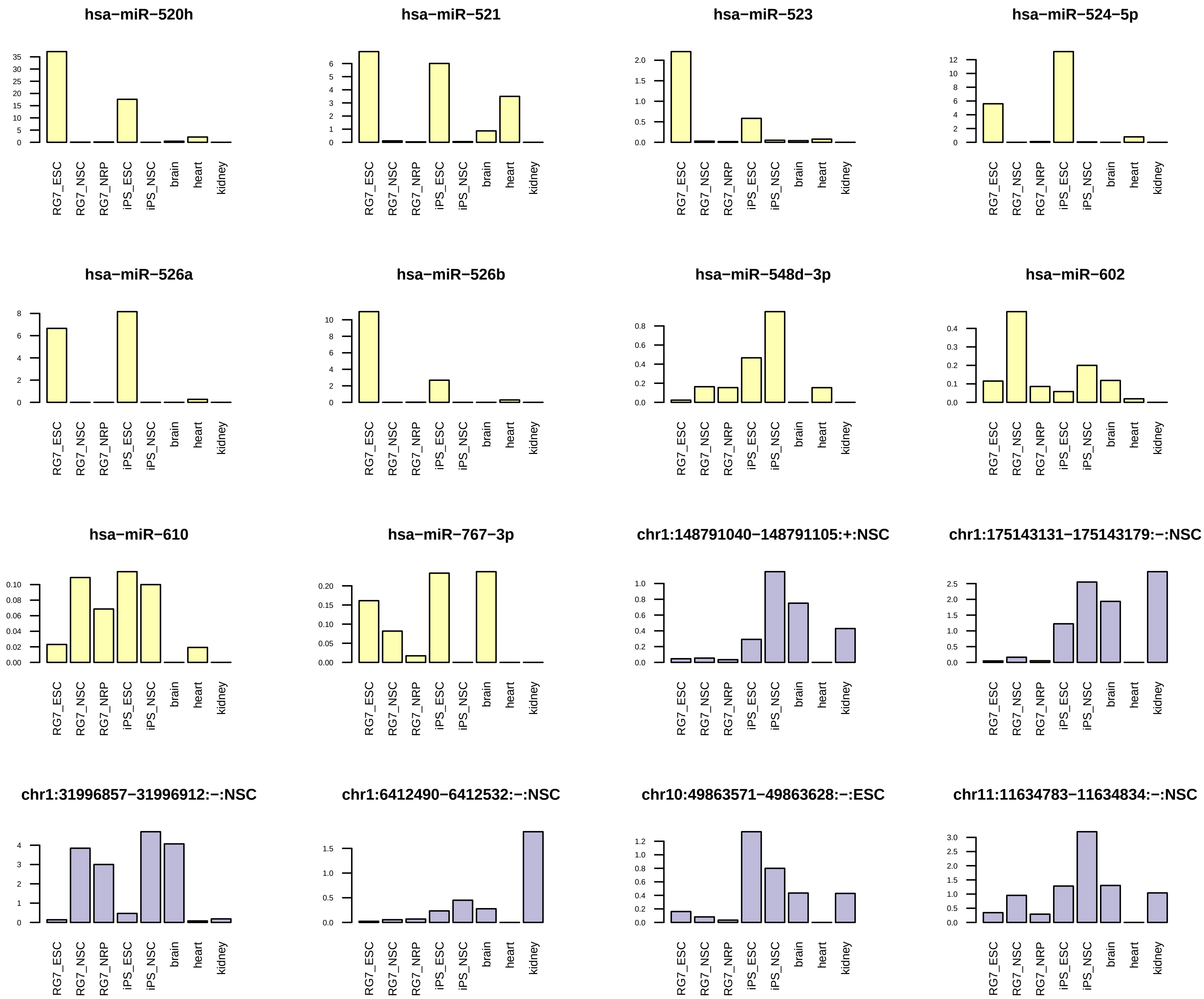
hsa−miR−520h
0
5
10
15
20
25
30
35
RG7_ESC
RG7_NSC
RG7_NRP
iPS_ESC
iPS_NSC
brain
heart
kidney
hsa−miR−521
0
1
2
3
4
5
6
RG7_ESC
RG7_NSC
RG7_NRP
iPS_ESC
iPS_NSC
brain
heart
kidney
hsa−miR−523
0.0
0.5
1.0
1.5
2.0
RG7_ESC
RG7_NSC
RG7_NRP
iPS_ESC
iPS_NSC
brain
heart
kidney
hsa−miR−524−5p
0
2
4
6
8
10
12
RG7_ESC
RG7_NSC
RG7_NRP
iPS_ESC
iPS_NSC
brain
heart
kidney
hsa−miR−526a
0
2
4
6
8
RG7_ESC
RG7_NSC
RG7_NRP
iPS_ESC
iPS_NSC
brain
heart
kidney
hsa−miR−526b
0
2
4
6
8
10
RG7_ESC
RG7_NSC
RG7_NRP
iPS_ESC
iPS_NSC
brain
heart
kidney
hsa−miR−548d−3p
0.0
0.2
0.4
0.6
0.8
RG7_ESC
RG7_NSC
RG7_NRP
iPS_ESC
iPS_NSC
brain
heart
kidney
hsa−miR−602
0.0
0.1
0.2
0.3
0.4
RG7_ESC
RG7_NSC
RG7_NRP
iPS_ESC
iPS_NSC
brain
heart
kidney
hsa−miR−610
0.00
0.02
0.04
0.06
0.08
0.10
RG7_ESC
RG7_NSC
RG7_NRP
iPS_ESC
iPS_NSC
brain
heart
kidney
hsa−miR−767−3p
0.00
0.05
0.10
0.15
0.20
RG7_ESC
RG7_NSC
RG7_NRP
iPS_ESC
iPS_NSC
brain
heart
kidney
chr1:148791040−148791105:+:NSC
0.0
0.2
0.4
0.6
0.8
1.0
RG7_ESC
RG7_NSC
RG7_NRP
iPS_ESC
iPS_NSC
brain
heart
kidney
chr1:175143131−175143179:−:NSC
0.0
0.5
1.0
1.5
2.0
2.5
RG7_ESC
RG7_NSC
RG7_NRP
iPS_ESC
iPS_NSC
brain
heart
kidney
chr1:31996857−31996912:−:NSC
0
1
2
3
4
RG7_ESC
RG7_NSC
RG7_NRP
iPS_ESC
iPS_NSC
brain
heart
kidney
chr1:6412490−6412532:−:NSC
0.0
0.5
1.0
1.5
RG7_ESC
RG7_NSC
RG7_NRP
iPS_ESC
iPS_NSC
brain
heart
kidney
chr10:49863571−49863628:−:ESC
0.0
0.2
0.4
0.6
0.8
1.0
1.2
RG7_ESC
RG7_NSC
RG7_NRP
iPS_ESC
iPS_NSC
brain
heart
kidney
chr11:11634783−11634834:−:NSC
0.0
0.5
1.0
1.5
2.0
2.5
3.0
RG7_ESC
RG7_NSC
RG7_NRP
iPS_ESC
iPS_NSC
brain
heart
kidney

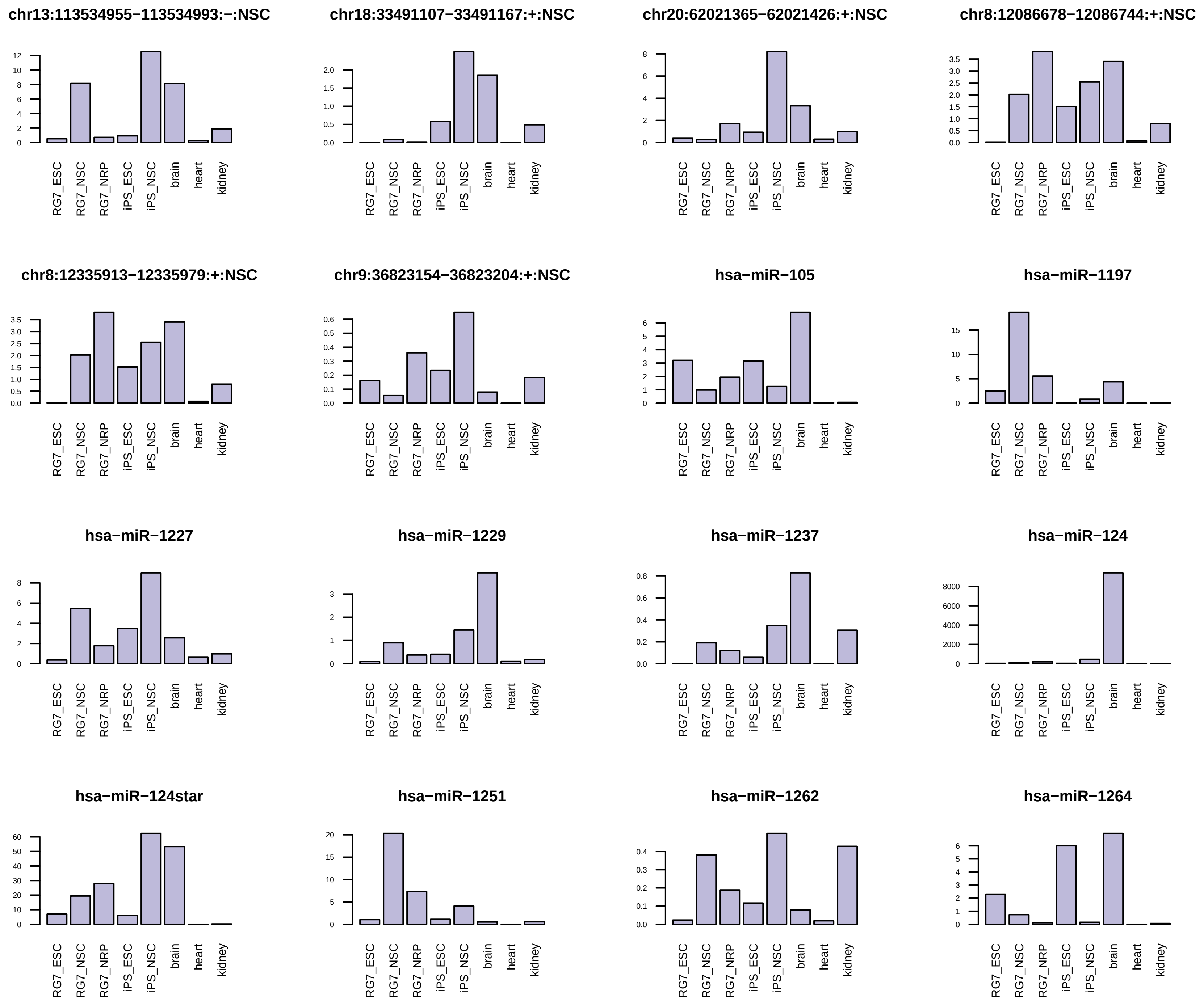
chr13:113534955−113534993:−:NSC
0
2
4
6
8
10
12
chr18:33491107−33491167:+:NSC
0.0
0.5
1.0
1.5
2.0
chr20:62021365−62021426:+:NSC
0
2
4
6
8
chr8:12086678−12086744:+:NSC
0.0
0.5
1.0
1.5
2.0
2.5
3.0
3.5
chr8:12335913−12335979:+:NSC
0.0
0.5
1.0
1.5
2.0
2.5
3.0
3.5
chr9:36823154−36823204:+:NSC
0.0
0.1
0.2
0.3
0.4
0.5
0.6
hsa−miR−105
0
1
2
3
4
5
6
hsa−miR−1197
0
5
10
15
hsa−miR−1227
0
2
4
6
8
hsa−miR−1229
0
1
2
3
hsa−miR−1237
0.0
0.2
0.4
0.6
0.8
hsa−miR−124
0
2000
4000
6000
8000
hsa−miR−124star
0
10
20
30
40
50
60
hsa−miR−1251
0
5
10
15
20
hsa−miR−1262
0.0
0.1
0.2
0.3
0.4
hsa−miR−1264
0
1
2
3
4
5
6
RG7_ESC
RG7_NSC
RG7_NRP
iPS_ESC
iPS_NSC
brain
heart
kidney

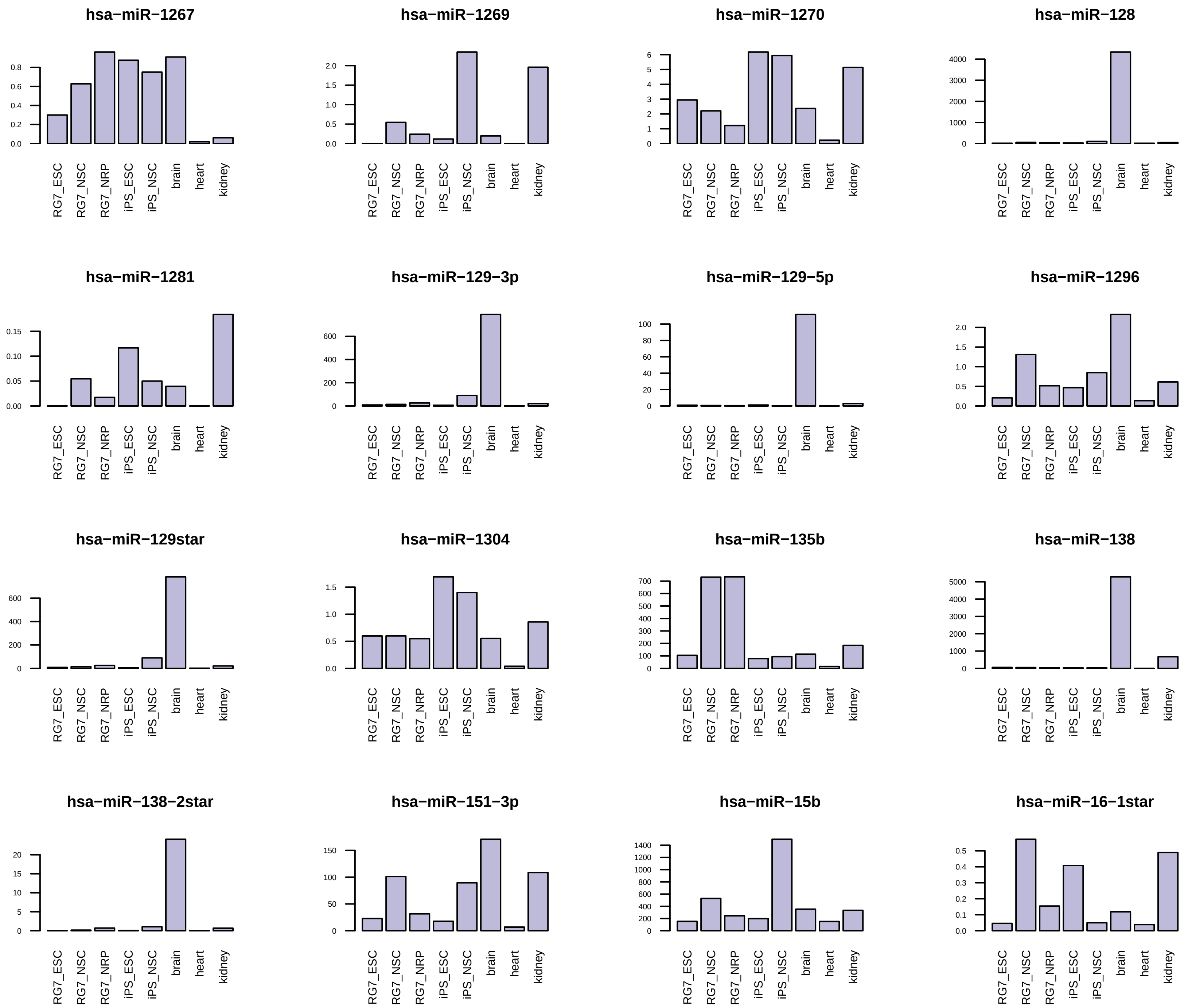
hsa−miR−1267
0.0 0.2 0.4 0.6 0.8
RG7_ESC RG7_NSC RG7_NRP iPS_ESC iPS_NSC brain heart kidney
hsa−miR−1269
0.0 0.5 1.0 1.5 2.0
RG7_ESC RG7_NSC RG7_NRP iPS_ESC iPS_NSC brain heart kidney
hsa−miR−1270
0 1 2 3 4 5 6
RG7_ESC RG7_NSC RG7_NRP iPS_ESC iPS_NSC brain heart kidney
hsa−miR−128
0 1000 2000 3000 4000
RG7_ESC RG7_NSC RG7_NRP iPS_ESC iPS_NSC brain heart kidney
hsa−miR−1281
0.00 0.05 0.10 0.15
RG7_ESC RG7_NSC RG7_NRP iPS_ESC iPS_NSC brain heart kidney
hsa−miR−129−3p
0 200 400 600
RG7_ESC RG7_NSC RG7_NRP iPS_ESC iPS_NSC brain heart kidney
hsa−miR−129−5p
0 20 40 60 80 100
RG7_ESC RG7_NSC RG7_NRP iPS_ESC iPS_NSC brain heart kidney
hsa−miR−1296
0.0 0.5 1.0 1.5 2.0
RG7_ESC RG7_NSC RG7_NRP iPS_ESC iPS_NSC brain heart kidney
hsa−miR−129star
0 200 400 600
RG7_ESC RG7_NSC RG7_NRP iPS_ESC iPS_NSC brain heart kidney
hsa−miR−1304
0.0 0.5 1.0 1.5
RG7_ESC RG7_NSC RG7_NRP iPS_ESC iPS_NSC brain heart kidney
hsa−miR−135b
0 100 200 300 400 500 600 700
RG7_ESC RG7_NSC RG7_NRP iPS_ESC iPS_NSC brain heart kidney
hsa−miR−138
0 1000 2000 3000 4000 5000
RG7_ESC RG7_NSC RG7_NRP iPS_ESC iPS_NSC brain heart kidney
hsa−miR−138−2star
0 5 10 15 20
RG7_ESC RG7_NSC RG7_NRP iPS_ESC iPS_NSC brain heart kidney
hsa−miR−151−3p
0 50 100 150
RG7_ESC RG7_NSC RG7_NRP iPS_ESC iPS_NSC brain heart kidney
hsa−miR−15b
0 200 400 600 800 1000 1200 1400
RG7_ESC RG7_NSC RG7_NRP iPS_ESC iPS_NSC brain heart kidney
hsa−miR−16−1star
0.0 0.1 0.2 0.3 0.4 0.5
RG7_ESC RG7_NSC RG7_NRP iPS_ESC iPS_NSC brain heart kidney

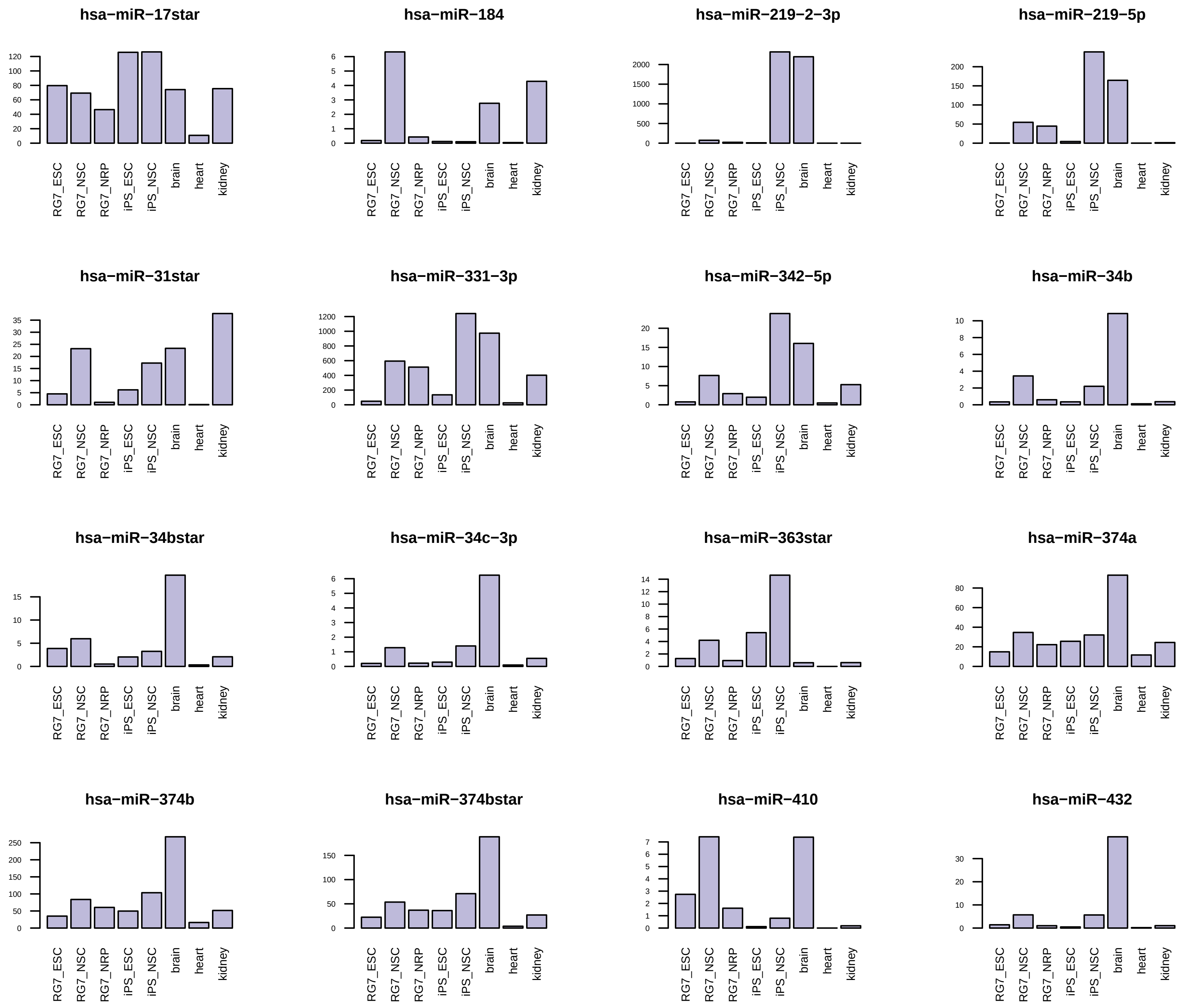
hsa−miR−17star
0
20
40
60
80
100
120
hsa−miR−184
0
1
2
3
4
5
6
hsa−miR−219−2−3p
0
500
1000
1500
2000
hsa−miR−219−5p
0
50
100
150
200
hsa−miR−31star
0
5
10
15
20
25
30
35
hsa−miR−331−3p
0
200
400
600
800
1000
1200
hsa−miR−342−5p
0
5
10
15
20
hsa−miR−34b
0
2
4
6
8
10
hsa−miR−34bstar
0
5
10
15
hsa−miR−34c−3p
0
1
2
3
4
5
6
hsa−miR−363star
0
2
4
6
8
10
12
14
hsa−miR−374a
0
20
40
60
80
hsa−miR−374b
0
50
100
150
200
250
hsa−miR−374bstar
0
50
100
150
hsa−miR−410
0
1
2
3
4
5
6
7
hsa−miR−432
0
10
20
30
RG7_ESC
RG7_NSC
RG7_NRP
iPS_ESC
iPS_NSC
brain
heart
kidney

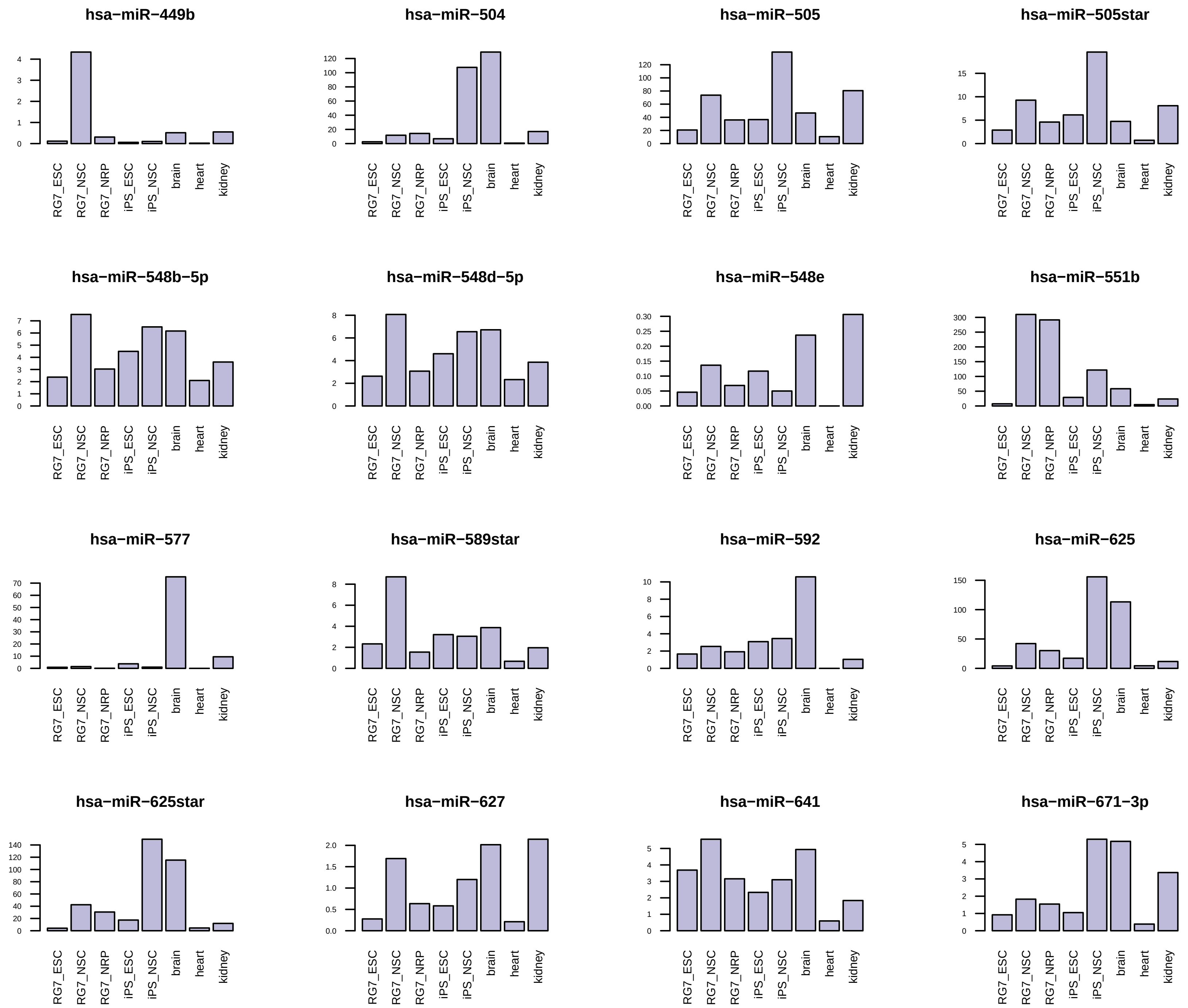
hsa−miR−449b
hsa−miR−504
hsa−miR−505
hsa−miR−505star
hsa−miR−548b−5p
hsa−miR−548d−5p
hsa−miR−548e
hsa−miR−551b
hsa−miR−577
hsa−miR−589star
hsa−miR−592
hsa−miR−625
hsa−miR−625star
hsa−miR−627
hsa−miR−641
hsa−miR−671−3p
RG7_ESC
RG7_NSC
RG7_NRP
iPS_ESC
iPS_NSC
brain
heart
kidney

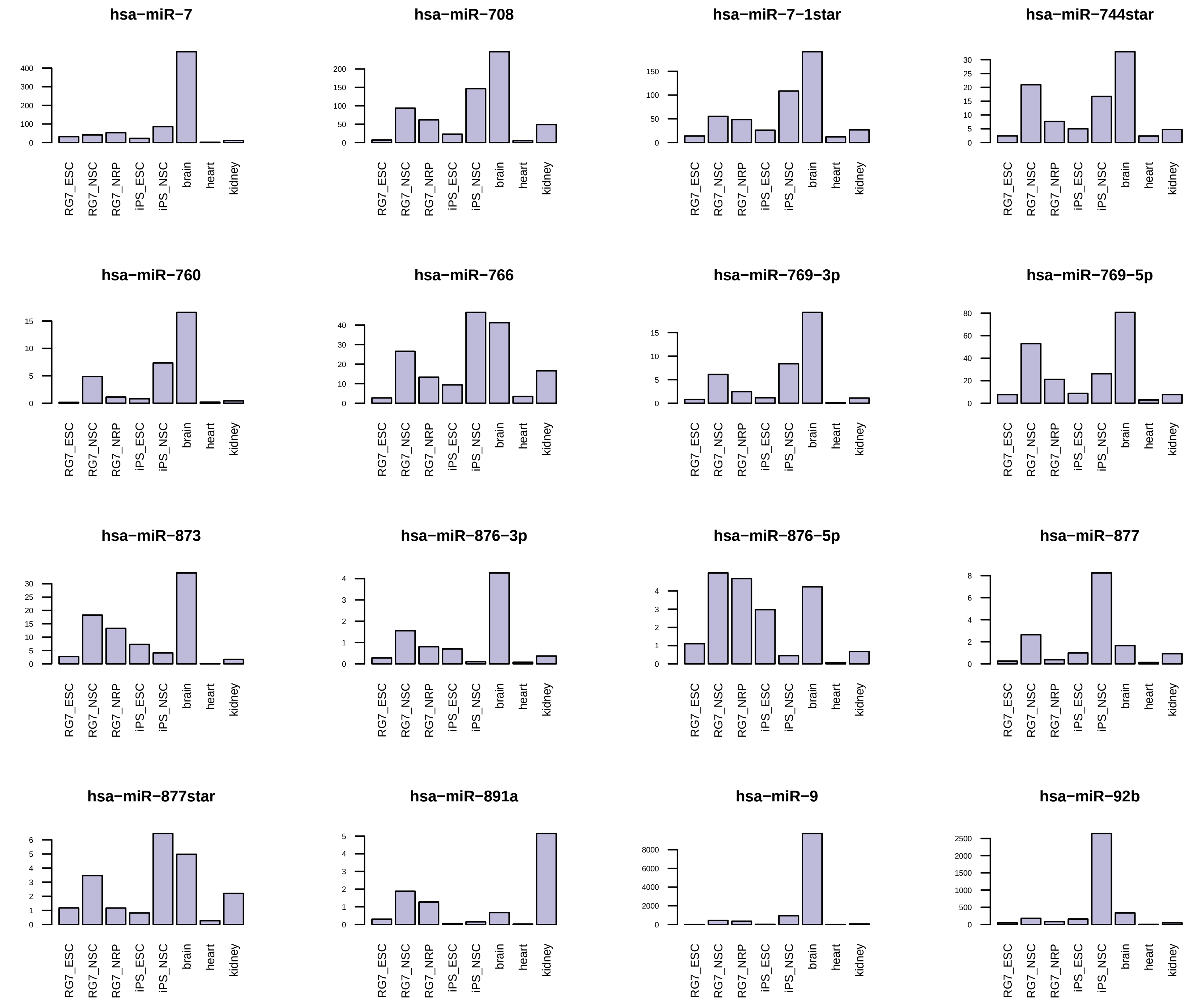
hsa−miR−7
400
300
200
100
0
hsa−miR−708
200
150
100
50
0
hsa−miR−7−1star
150
100
50
0
hsa−miR−744star
30
25
20
15
10
5
0
hsa−miR−760
15
10
5
0
hsa−miR−766
40
30
20
10
0
hsa−miR−769−3p
15
10
5
0
hsa−miR−769−5p
80
60
40
20
0
hsa−miR−873
30
25
20
15
10
5
0
hsa−miR−876−3p
4
3
2
1
0
hsa−miR−876−5p
4
3
2
1
0
hsa−miR−877
8
6
4
2
0
hsa−miR−877star
6
5
4
3
2
1
0
hsa−miR−891a
5
4
3
2
1
0
hsa−miR−9
8000
6000
4000
2000
0
hsa−miR−92b
2500
2000
1500
1000
500
0
RG7_ESC
RG7_NSC
RG7_NRP
iPS_ESC
iPS_NSC
brain
heart
kidney

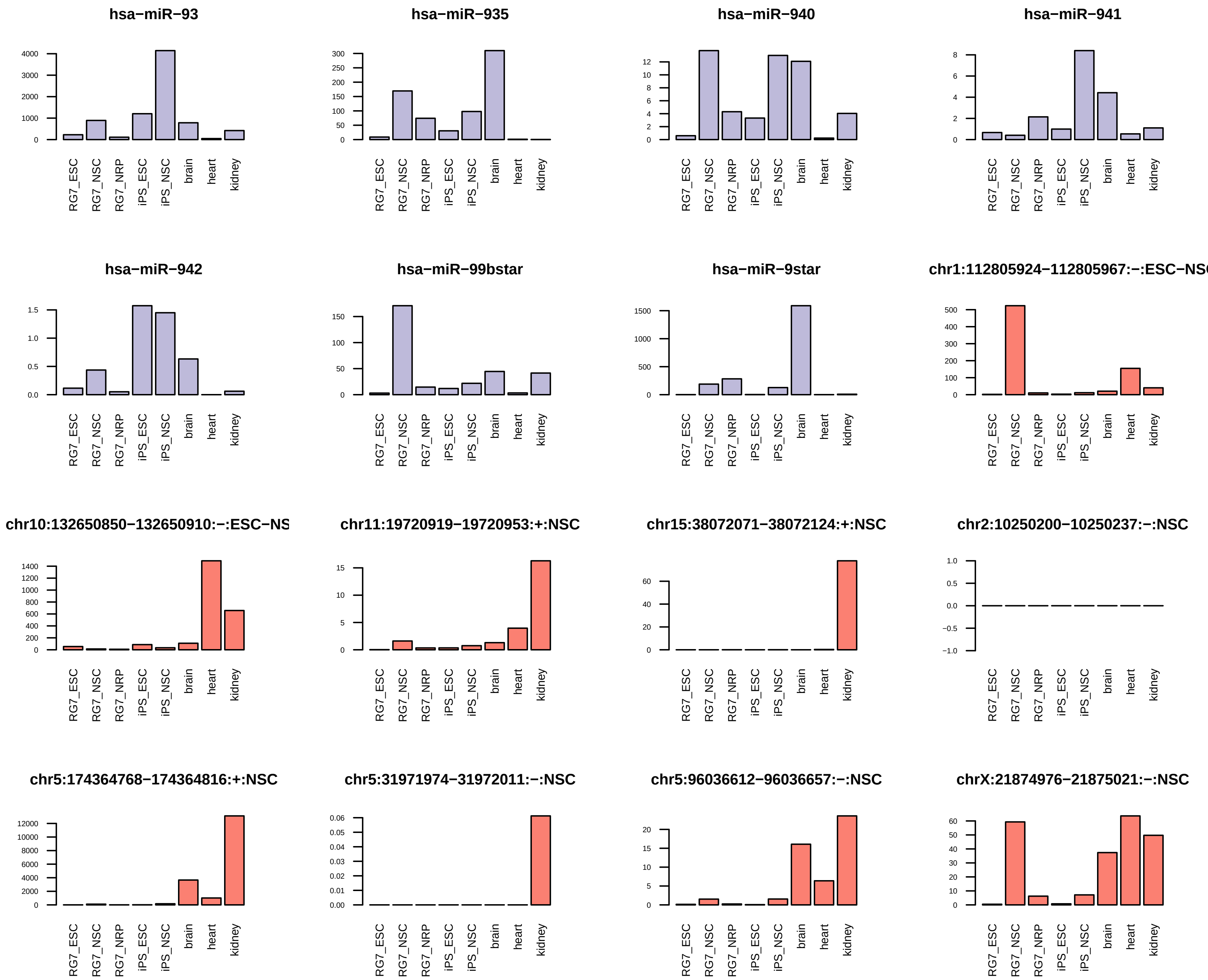
hsa−miR−93
hsa−miR−935
hsa−miR−940
hsa−miR−941
hsa−miR−942
hsa−miR−99bstar
hsa−miR−9star
chr1:112805924−112805967:−:ESC−NSC
chr10:132650850−132650910:−:ESC−NS
chr11:19720919−19720953:+:NSC
chr15:38072071−38072124:+:NSC
chr2:10250200−10250237:−:NSC
chr5:174364768−174364816:+:NSC
chr5:31971974−31972011:−:NSC
chr5:96036612−96036657:−:NSC
chrX:21874976−21875021:−:NSC
RG7_ESC
RG7_NSC
RG7_NRP
iPS_ESC
iPS_NSC
brain
heart
kidney

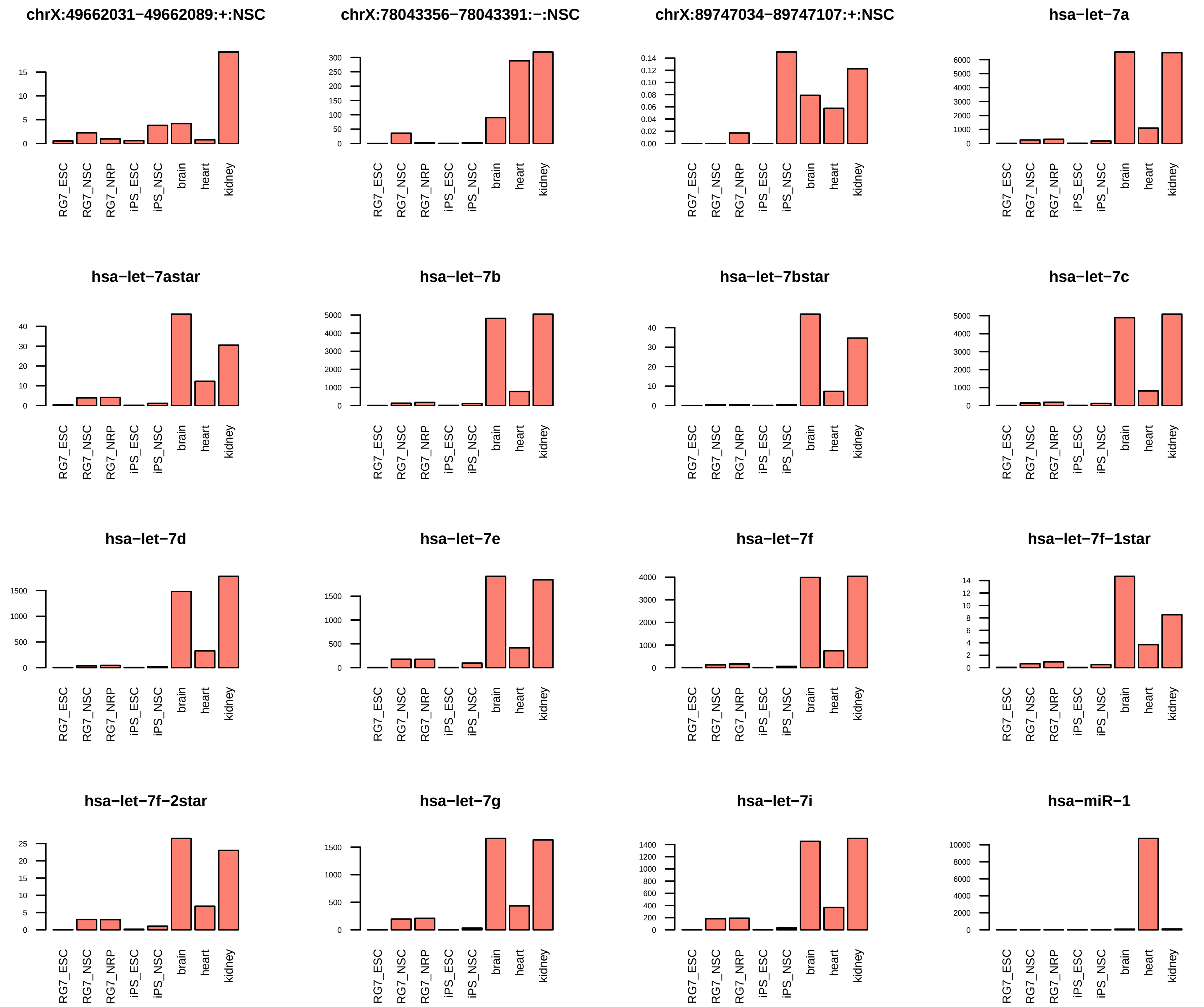
chrX:49662031−49662089:+:NSC
0
5
10
15
chrX:78043356−78043391:−:NSC
0
50
100
150
200
250
300
chrX:89747034−89747107:+:NSC
0.00
0.02
0.04
0.06
0.08
0.10
0.12
0.14
hsa−let−7a
0
1000
2000
3000
4000
5000
6000
hsa−let−7astar
0
10
20
30
40
hsa−let−7b
0
1000
2000
3000
4000
5000
hsa−let−7bstar
0
10
20
30
40
hsa−let−7c
0
1000
2000
3000
4000
5000
hsa−let−7d
0
500
1000
1500
hsa−let−7e
0
500
1000
1500
hsa−let−7f
0
1000
2000
3000
4000
hsa−let−7f−1star
0
2
4
6
8
10
12
14
hsa−let−7f−2star
0
5
10
15
20
25
hsa−let−7g
0
500
1000
1500
hsa−let−7i
0
200
400
600
800
1000
1200
1400
hsa−miR−1
0
2000
4000
6000
8000
10000
RG7_ESC
RG7_NSC
RG7_NRP
iPS_ESC
iPS_NSC
brain
heart
kidney

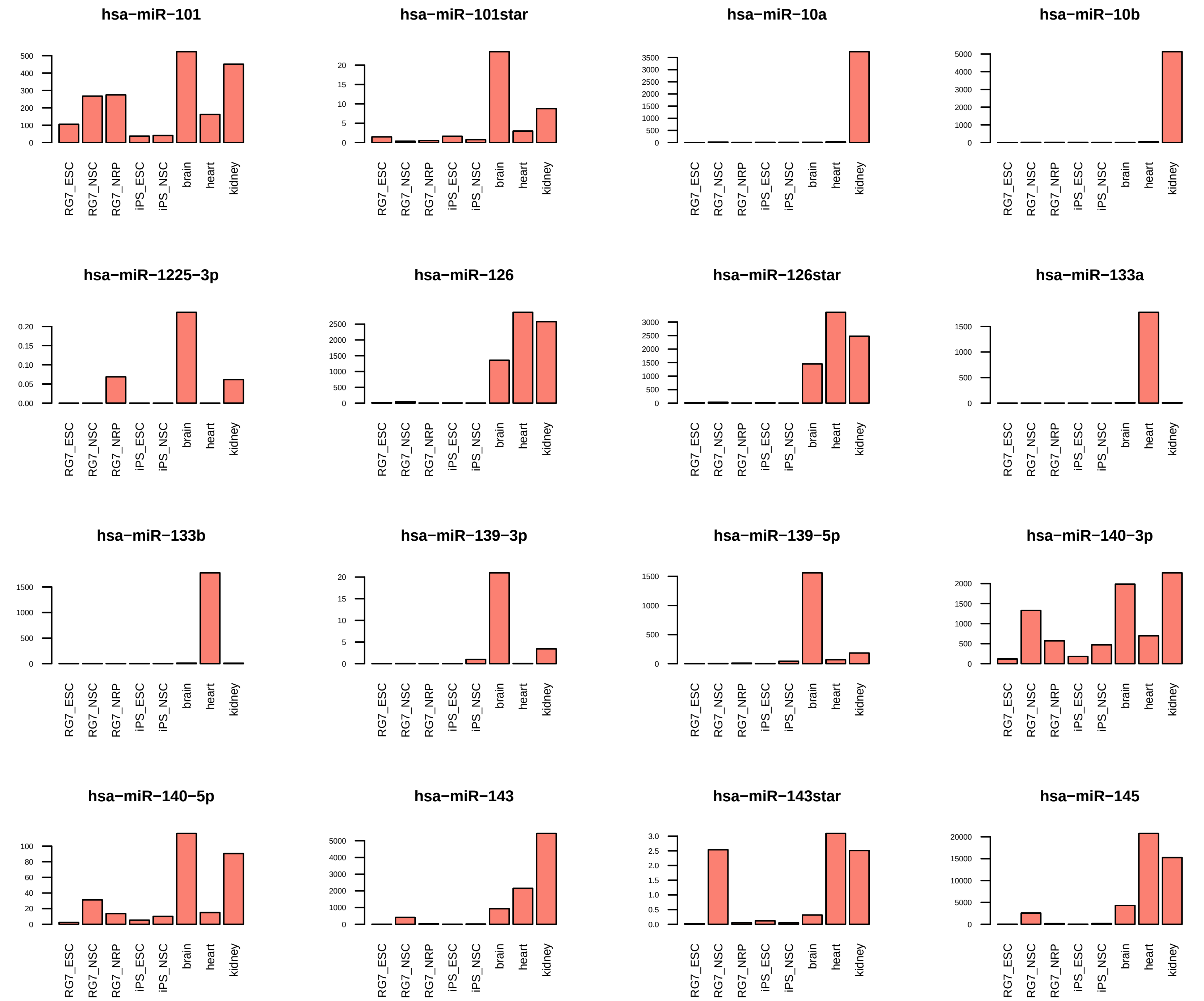
hsa−miR−101
0
100
200
300
400
500
hsa−miR−101star
0
5
10
15
20
hsa−miR−10a
0
500
1000
1500
2000
2500
3000
3500
hsa−miR−10b
0
1000
2000
3000
4000
5000
hsa−miR−1225−3p
0.00
0.05
0.10
0.15
0.20
hsa−miR−126
0
500
1000
1500
2000
2500
hsa−miR−126star
0
500
1000
1500
2000
2500
3000
hsa−miR−133a
0
500
1000
1500
hsa−miR−133b
0
500
1000
1500
hsa−miR−139−3p
0
5
10
15
20
hsa−miR−139−5p
0
500
1000
1500
hsa−miR−140−3p
0
500
1000
1500
2000
hsa−miR−140−5p
0
20
40
60
80
100
hsa−miR−143
0
1000
2000
3000
4000
5000
hsa−miR−143star
0.0
0.5
1.0
1.5
2.0
2.5
3.0
hsa−miR−145
0
5000
10000
15000
20000
RG7_ESC
RG7_NSC
RG7_NRP
iPS_ESC
iPS_NSC
brain
heart
kidney

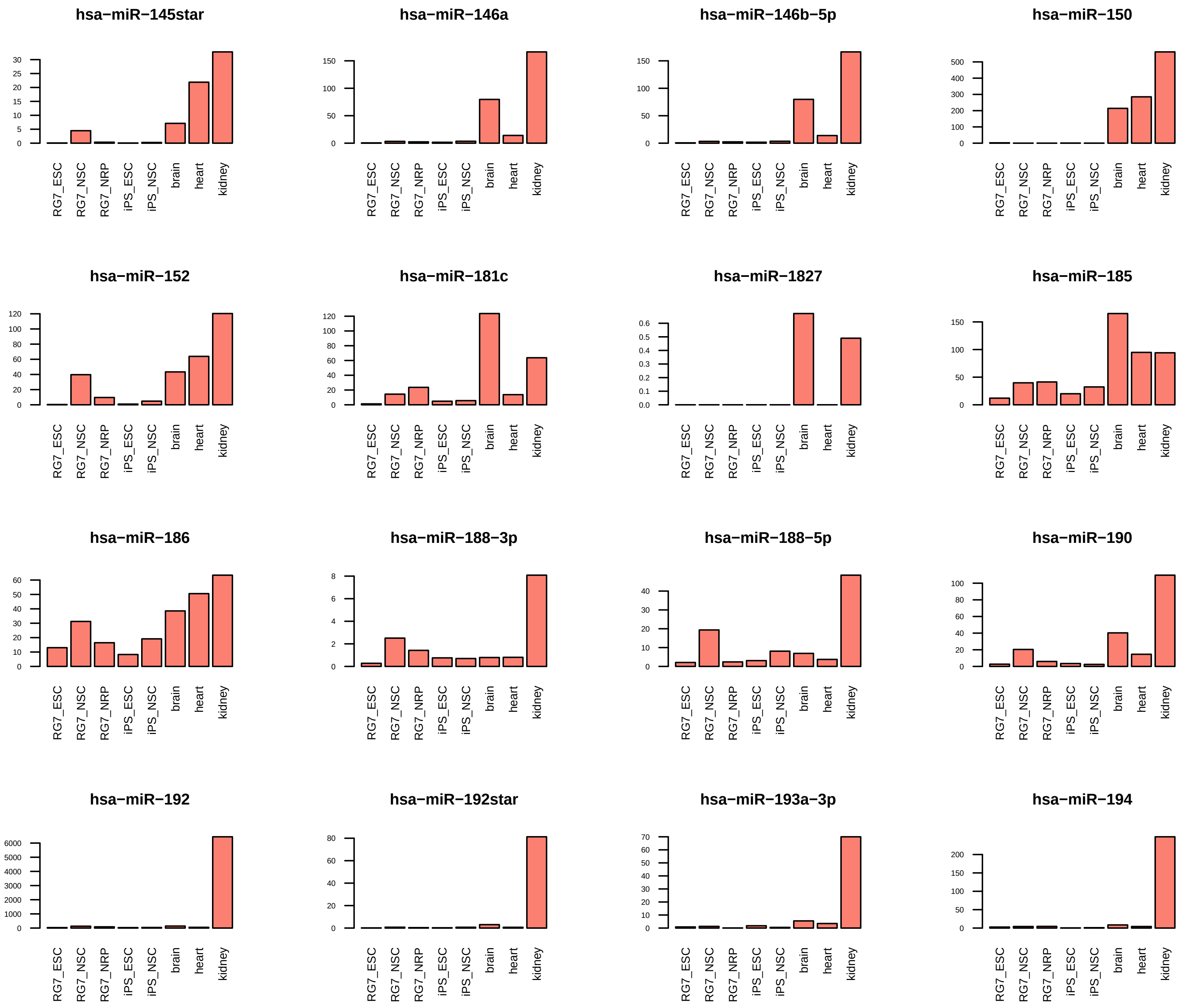
hsa−miR−145star
0
5
10
15
20
25
30
hsa−miR−146a
0
50
100
150
hsa−miR−146b−5p
0
50
100
150
hsa−miR−150
0
100
200
300
400
500
hsa−miR−152
0
20
40
60
80
100
120
hsa−miR−181c
0
20
40
60
80
100
120
hsa−miR−1827
0.0
0.1
0.2
0.3
0.4
0.5
0.6
hsa−miR−185
0
50
100
150
hsa−miR−186
0
10
20
30
40
50
60
hsa−miR−188−3p
0
2
4
6
8
hsa−miR−188−5p
0
10
20
30
40
hsa−miR−190
0
20
40
60
80
100
hsa−miR−192
0
1000
2000
3000
4000
5000
6000
hsa−miR−192star
0
20
40
60
80
hsa−miR−193a−3p
0
10
20
30
40
50
60
70
hsa−miR−194
0
50
100
150
200
RG7_ESC
RG7_NSC
RG7_NRP
iPS_ESC
iPS_NSC
brain
heart
kidney

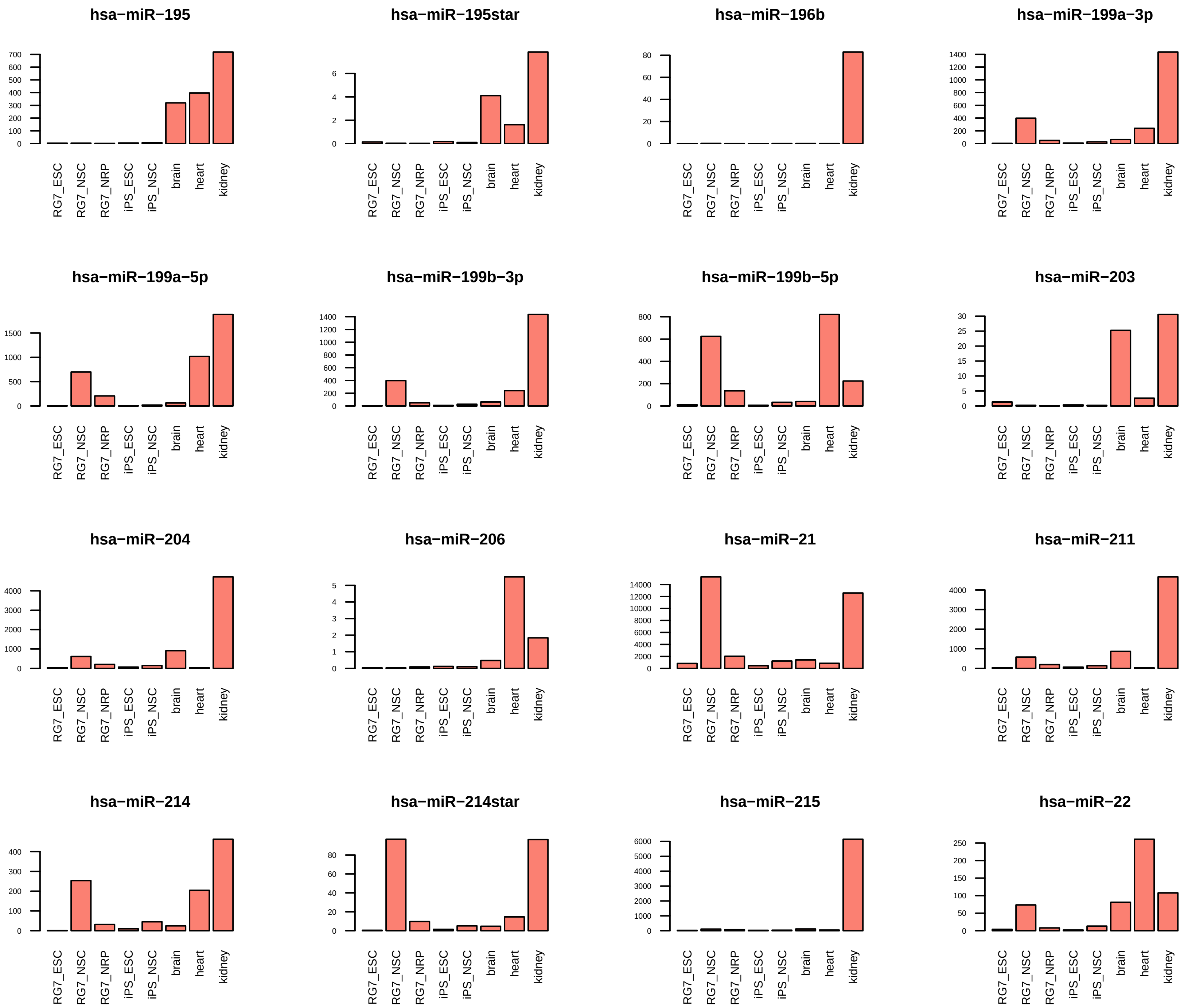
hsa−miR−195
0 100 200 300 400 500 600 700
RG7_ESC RG7_NSC RG7_NRP iPS_ESC iPS_NSC brain heart kidney
hsa−miR−195star
0 2 4 6
RG7_ESC RG7_NSC RG7_NRP iPS_ESC iPS_NSC brain heart kidney
hsa−miR−196b
0 20 40 60 80
RG7_ESC RG7_NSC RG7_NRP iPS_ESC iPS_NSC brain heart kidney
hsa−miR−199a−3p
0 200 400 600 800 1000 1200 1400
RG7_ESC RG7_NSC RG7_NRP iPS_ESC iPS_NSC brain heart kidney
hsa−miR−199a−5p
0 500 1000 1500
RG7_ESC RG7_NSC RG7_NRP iPS_ESC iPS_NSC brain heart kidney
hsa−miR−199b−3p
0 200 400 600 800 1000 1200 1400
RG7_ESC RG7_NSC RG7_NRP iPS_ESC iPS_NSC brain heart kidney
hsa−miR−199b−5p
0 200 400 600 800
RG7_ESC RG7_NSC RG7_NRP iPS_ESC iPS_NSC brain heart kidney
hsa−miR−203
0 5 10 15 20 25 30
RG7_ESC RG7_NSC RG7_NRP iPS_ESC iPS_NSC brain heart kidney
hsa−miR−204
0 1000 2000 3000 4000
RG7_ESC RG7_NSC RG7_NRP iPS_ESC iPS_NSC brain heart kidney
hsa−miR−206
0 1 2 3 4 5
RG7_ESC RG7_NSC RG7_NRP iPS_ESC iPS_NSC brain heart kidney
hsa−miR−21
0 2000 4000 6000 8000 10000 12000 14000
RG7_ESC RG7_NSC RG7_NRP iPS_ESC iPS_NSC brain heart kidney
hsa−miR−211
0 1000 2000 3000 4000
RG7_ESC RG7_NSC RG7_NRP iPS_ESC iPS_NSC brain heart kidney
hsa−miR−214
0 100 200 300 400
RG7_ESC RG7_NSC RG7_NRP iPS_ESC iPS_NSC brain heart kidney
hsa−miR−214star
0 20 40 60 80
RG7_ESC RG7_NSC RG7_NRP iPS_ESC iPS_NSC brain heart kidney
hsa−miR−215
0 1000 2000 3000 4000 5000 6000
RG7_ESC RG7_NSC RG7_NRP iPS_ESC iPS_NSC brain heart kidney
hsa−miR−22
0 50 100 150 200 250
RG7_ESC RG7_NSC RG7_NRP iPS_ESC iPS_NSC brain heart kidney

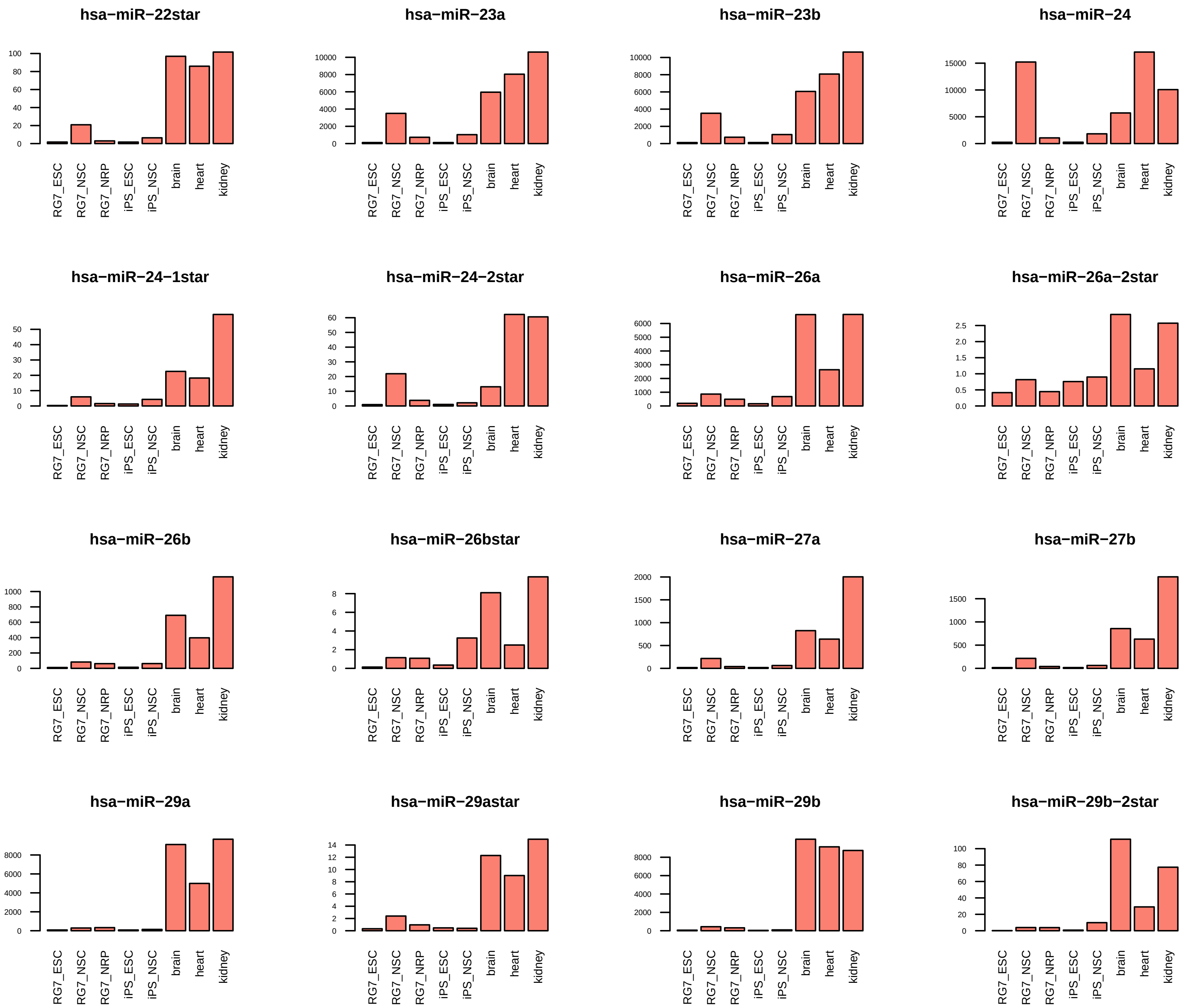
hsa−miR−22star
100
80
60
40
20
0
hsa−miR−23a
10000
8000
6000
4000
2000
0
hsa−miR−23b
10000
8000
6000
4000
2000
0
hsa−miR−24
15000
10000
5000
0
hsa−miR−24−1star
50
40
30
20
10
0
hsa−miR−24−2star
60
50
40
30
20
10
0
hsa−miR−26a
6000
5000
4000
3000
2000
1000
0
hsa−miR−26a−2star
2.5
2.0
1.5
1.0
0.5
0.0
hsa−miR−26b
1000
800
600
400
200
0
hsa−miR−26bstar
8
6
4
2
0
hsa−miR−27a
2000
1500
1000
500
0
hsa−miR−27b
1500
1000
500
0
hsa−miR−29a
8000
6000
4000
2000
0
hsa−miR−29astar
14
12
10
8
6
4
2
0
hsa−miR−29b
8000
6000
4000
2000
0
hsa−miR−29b−2star
100
80
60
40
20
0
RG7_ESC
RG7_NSC
RG7_NRP
iPS_ESC
iPS_NSC
brain
heart
kidney

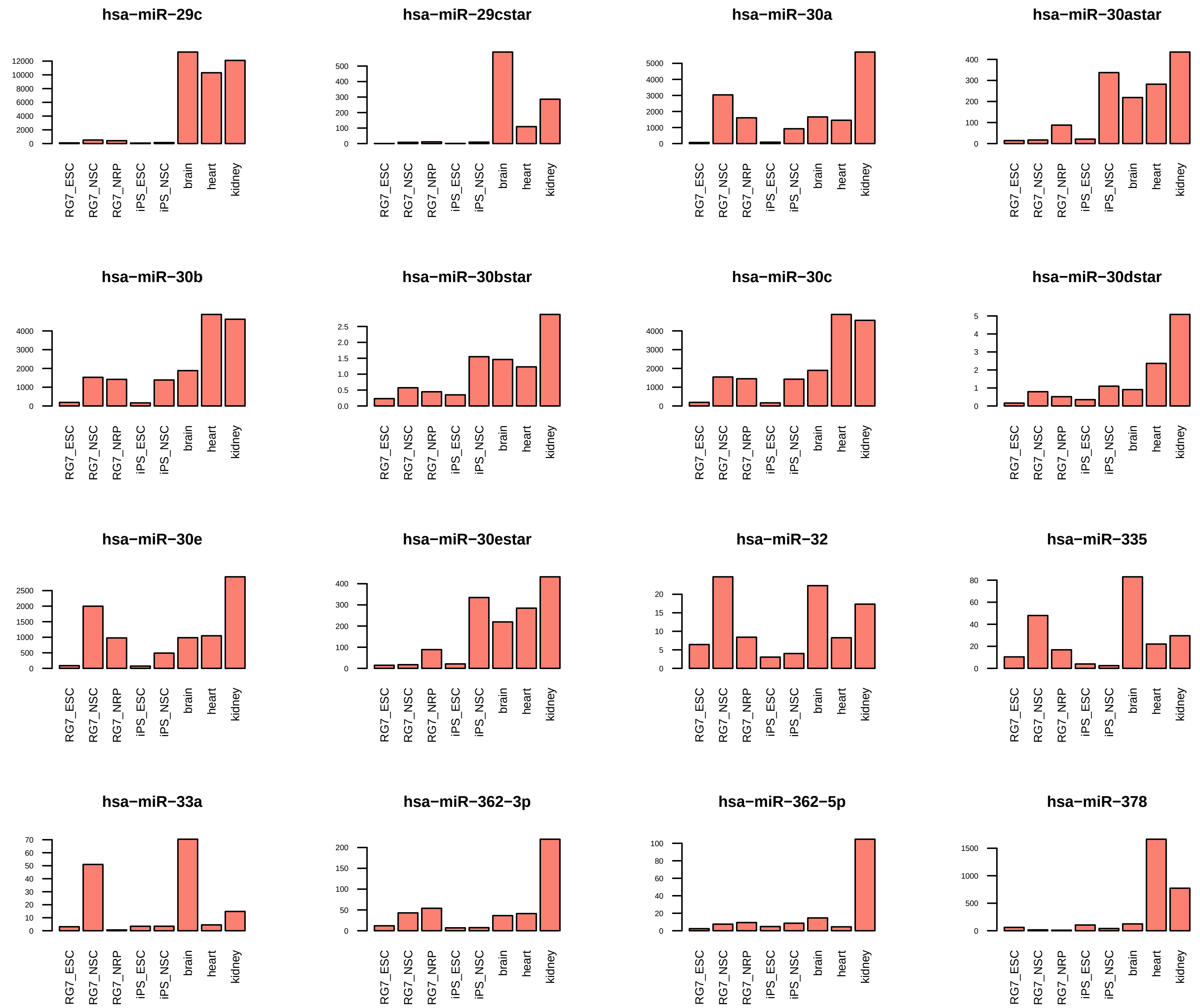
hsa−miR−29c
0
2000
4000
6000
8000
10000
12000
hsa−miR−29cstar
0
100
200
300
400
500
hsa−miR−30a
0
1000
2000
3000
4000
5000
hsa−miR−30astar
0
100
200
300
400
hsa−miR−30b
0
1000
2000
3000
4000
hsa−miR−30bstar
0.0
0.5
1.0
1.5
2.0
2.5
hsa−miR−30c
0
1000
2000
3000
4000
hsa−miR−30dstar
0
1
2
3
4
5
hsa−miR−30e
0
500
1000
1500
2000
2500
hsa−miR−30estar
0
100
200
300
400
hsa−miR−32
0
5
10
15
20
hsa−miR−335
0
20
40
60
80
hsa−miR−33a
0
10
20
30
40
50
60
70
hsa−miR−362−3p
0
50
100
150
200
hsa−miR−362−5p
0
20
40
60
80
100
hsa−miR−378
0
500
1000
1500
RG7_ESC
RG7_NSC
RG7_NRP
iPS_ESC
iPS_NSC
brain
heart
kidney

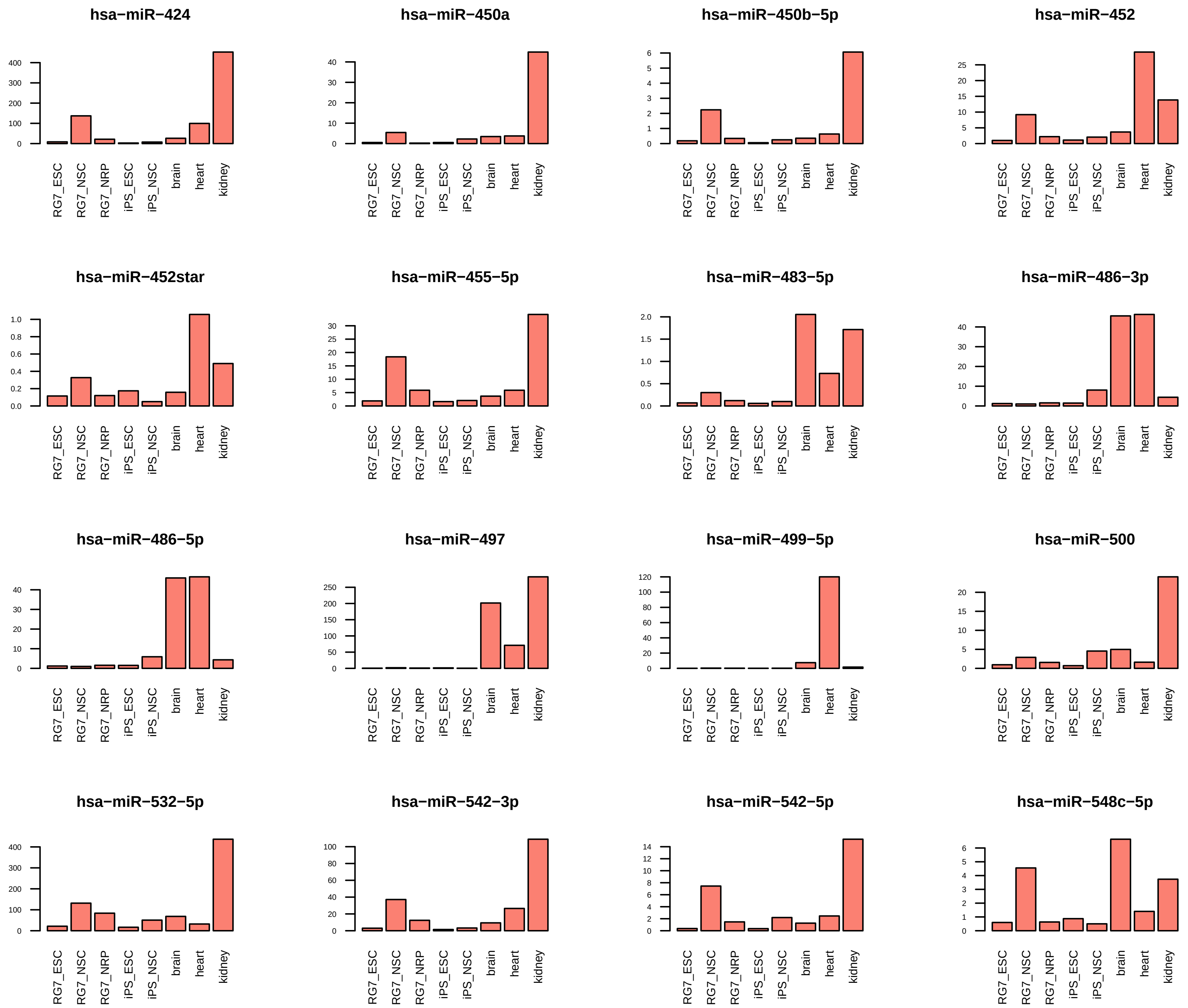
hsa−miR−424
0 100 200 300 400
RG7_ESC RG7_NSC RG7_NRP iPS_ESC iPS_NSC brain heart kidney
hsa−miR−450a
0 10 20 30 40
RG7_ESC RG7_NSC RG7_NRP iPS_ESC iPS_NSC brain heart kidney
hsa−miR−450b−5p
0 1 2 3 4 5 6
RG7_ESC RG7_NSC RG7_NRP iPS_ESC iPS_NSC brain heart kidney
hsa−miR−452
0 5 10 15 20 25
RG7_ESC RG7_NSC RG7_NRP iPS_ESC iPS_NSC brain heart kidney
hsa−miR−452star
0.0 0.2 0.4 0.6 0.8 1.0
RG7_ESC RG7_NSC RG7_NRP iPS_ESC iPS_NSC brain heart kidney
hsa−miR−455−5p
0 5 10 15 20 25 30
RG7_ESC RG7_NSC RG7_NRP iPS_ESC iPS_NSC brain heart kidney
hsa−miR−483−5p
0.0 0.5 1.0 1.5 2.0
RG7_ESC RG7_NSC RG7_NRP iPS_ESC iPS_NSC brain heart kidney
hsa−miR−486−3p
0 10 20 30 40
RG7_ESC RG7_NSC RG7_NRP iPS_ESC iPS_NSC brain heart kidney
hsa−miR−486−5p
0 10 20 30 40
RG7_ESC RG7_NSC RG7_NRP iPS_ESC iPS_NSC brain heart kidney
hsa−miR−497
0 50 100 150 200 250
RG7_ESC RG7_NSC RG7_NRP iPS_ESC iPS_NSC brain heart kidney
hsa−miR−499−5p
0 20 40 60 80 100 120
RG7_ESC RG7_NSC RG7_NRP iPS_ESC iPS_NSC brain heart kidney
hsa−miR−500
0 5 10 15 20
RG7_ESC RG7_NSC RG7_NRP iPS_ESC iPS_NSC brain heart kidney
hsa−miR−532−5p
0 100 200 300 400
RG7_ESC RG7_NSC RG7_NRP iPS_ESC iPS_NSC brain heart kidney
hsa−miR−542−3p
0 20 40 60 80 100
RG7_ESC RG7_NSC RG7_NRP iPS_ESC iPS_NSC brain heart kidney
hsa−miR−542−5p
0 2 4 6 8 10 12 14
RG7_ESC RG7_NSC RG7_NRP iPS_ESC iPS_NSC brain heart kidney
hsa−miR−548c−5p
0 1 2 3 4 5 6
RG7_ESC RG7_NSC RG7_NRP iPS_ESC iPS_NSC brain heart kidney

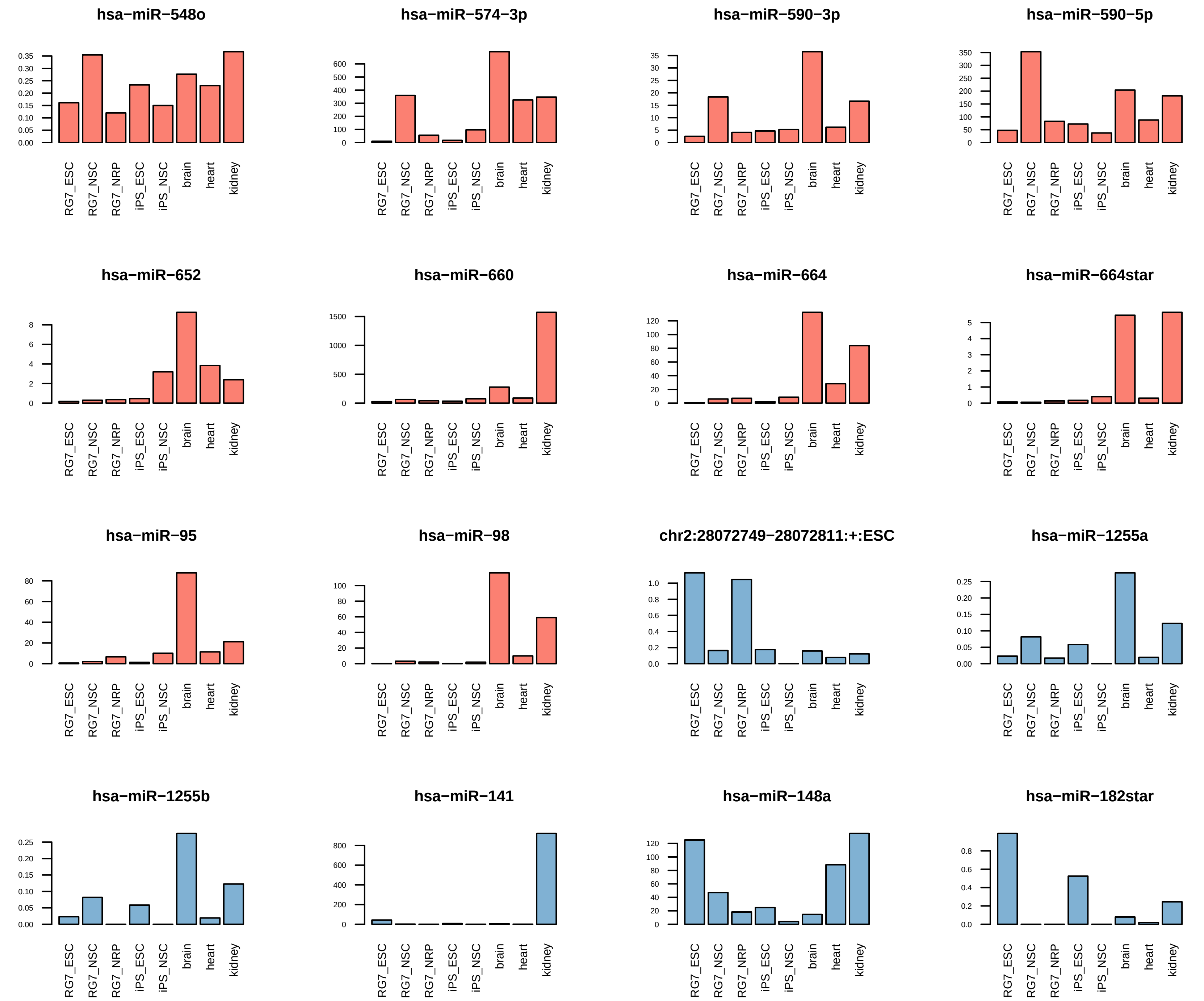

hsa−miR−548o
0.00 0.05 0.10 0.15 0.20 0.25 0.30 0.35
hsa−miR−574−3p
0 100 200 300 400 500 600
hsa−miR−590−3p
0 5 10 15 20 25 30 35
hsa−miR−590−5p
0 50 100 150 200 250 300 350
hsa−miR−652
0 2 4 6 8
hsa−miR−660
0 500 1000 1500
hsa−miR−664
0 20 40 60 80 100 120
hsa−miR−664star
0 1 2 3 4 5
hsa−miR−95
0 20 40 60 80
hsa−miR−98
0 20 40 60 80 100
chr2:28072749−28072811:+:ESC
0.0 0.2 0.4 0.6 0.8 1.0
hsa−miR−1255a
0.00 0.05 0.10 0.15 0.20 0.25
hsa−miR−1255b
0.00 0.05 0.10 0.15 0.20 0.25
hsa−miR−141
0 200 400 600 800
hsa−miR−148a
0 20 40 60 80 100 120
hsa−miR−182star
0.0 0.2 0.4 0.6 0.8
RG7_ESC
RG7_NSC
RG7_NRP
iPS_ESC
iPS_NSC
brain
heart
kidney

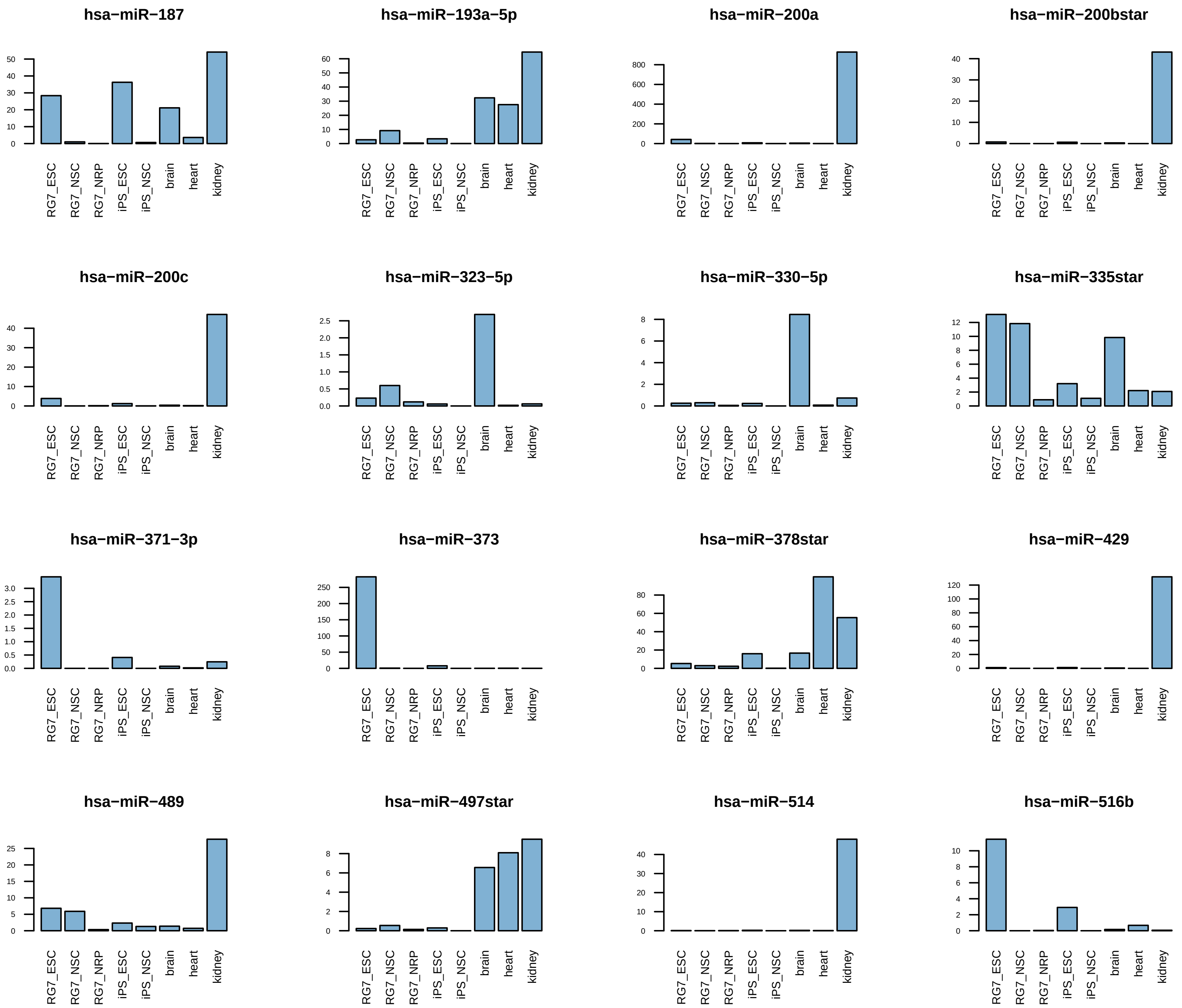
hsa−miR−187
0
10
20
30
40
50
RG7_ESC
RG7_NSC
RG7_NRP
iPS_ESC
iPS_NSC
brain
heart
kidney
hsa−miR−193a−5p
0
10
20
30
40
50
60
RG7_ESC
RG7_NSC
RG7_NRP
iPS_ESC
iPS_NSC
brain
heart
kidney
hsa−miR−200a
0
200
400
600
800
RG7_ESC
RG7_NSC
RG7_NRP
iPS_ESC
iPS_NSC
brain
heart
kidney
hsa−miR−200bstar
0
10
20
30
40
RG7_ESC
RG7_NSC
RG7_NRP
iPS_ESC
iPS_NSC
brain
heart
kidney
hsa−miR−200c
0
10
20
30
40
RG7_ESC
RG7_NSC
RG7_NRP
iPS_ESC
iPS_NSC
brain
heart
kidney
hsa−miR−323−5p
0.0
0.5
1.0
1.5
2.0
2.5
RG7_ESC
RG7_NSC
RG7_NRP
iPS_ESC
iPS_NSC
brain
heart
kidney
hsa−miR−330−5p
0
2
4
6
8
RG7_ESC
RG7_NSC
RG7_NRP
iPS_ESC
iPS_NSC
brain
heart
kidney
hsa−miR−335star
0
2
4
6
8
10
12
RG7_ESC
RG7_NSC
RG7_NRP
iPS_ESC
iPS_NSC
brain
heart
kidney
hsa−miR−371−3p
0.0
0.5
1.0
1.5
2.0
2.5
3.0
RG7_ESC
RG7_NSC
RG7_NRP
iPS_ESC
iPS_NSC
brain
heart
kidney
hsa−miR−373
0
50
100
150
200
250
RG7_ESC
RG7_NSC
RG7_NRP
iPS_ESC
iPS_NSC
brain
heart
kidney
hsa−miR−378star
0
20
40
60
80
RG7_ESC
RG7_NSC
RG7_NRP
iPS_ESC
iPS_NSC
brain
heart
kidney
hsa−miR−429
0
20
40
60
80
100
120
RG7_ESC
RG7_NSC
RG7_NRP
iPS_ESC
iPS_NSC
brain
heart
kidney
hsa−miR−489
0
5
10
15
20
25
RG7_ESC
RG7_NSC
RG7_NRP
iPS_ESC
iPS_NSC
brain
heart
kidney
hsa−miR−497star
0
2
4
6
8
RG7_ESC
RG7_NSC
RG7_NRP
iPS_ESC
iPS_NSC
brain
heart
kidney
hsa−miR−514
0
10
20
30
40
RG7_ESC
RG7_NSC
RG7_NRP
iPS_ESC
iPS_NSC
brain
heart
kidney
hsa−miR−516b
0
2
4
6
8
10
RG7_ESC
RG7_NSC
RG7_NRP
iPS_ESC
iPS_NSC
brain
heart
kidney

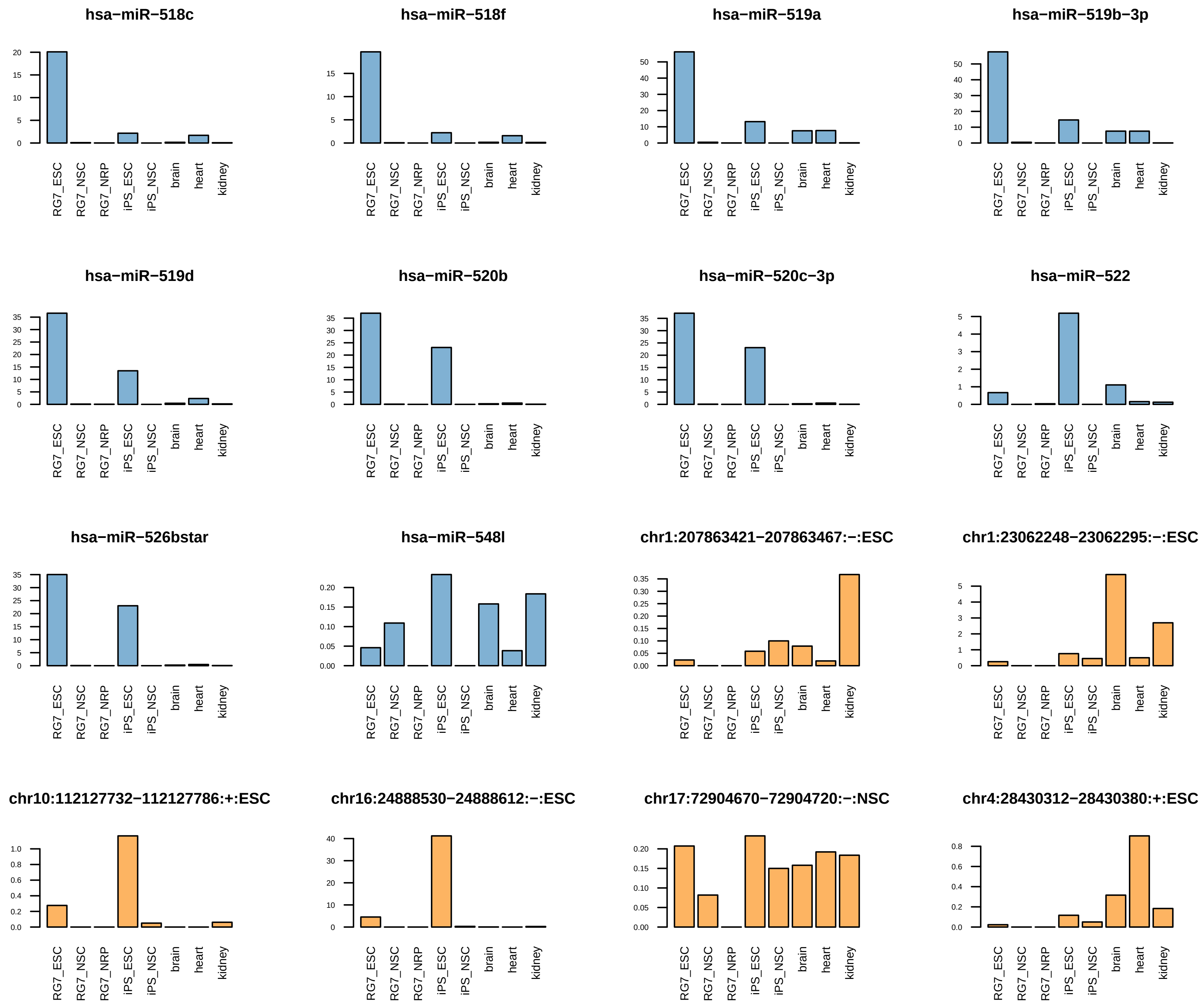

hsa−miR−518c
hsa−miR−518f
hsa−miR−519a
hsa−miR−519b−3p
hsa−miR−519d
hsa−miR−520b
hsa−miR−520c−3p
hsa−miR−522
hsa−miR−526bstar
hsa−miR−548l
chr1:207863421−207863467:−:ESC
chr1:23062248−23062295:−:ESC
chr10:112127732−112127786:+:ESC
chr16:24888530−24888612:−:ESC
chr17:72904670−72904720:−:NSC
chr4:28430312−28430380:+:ESC
RG7_ESC
RG7_NSC
RG7_NRP
iPS_ESC
iPS_NSC
brain
heart
kidney

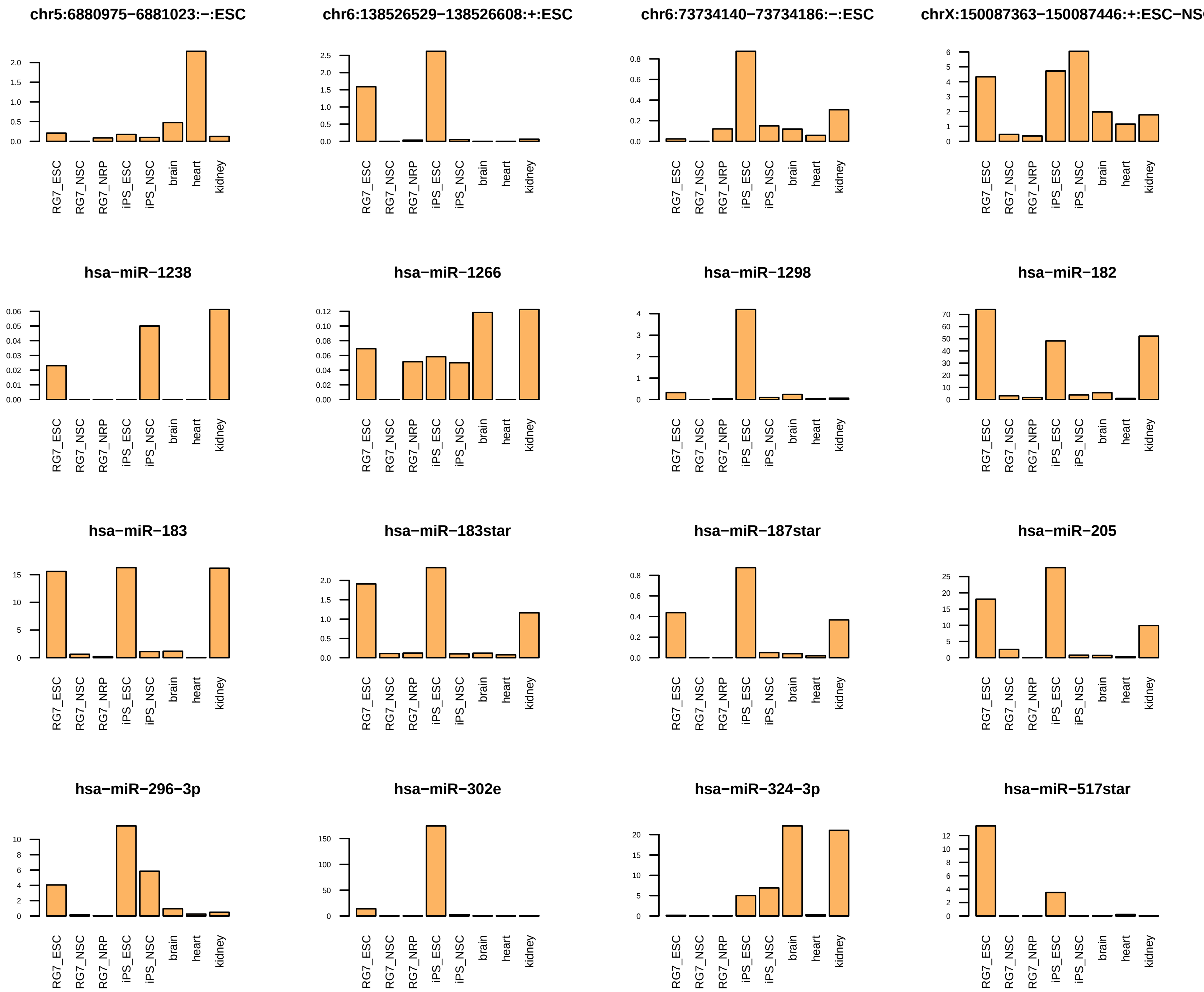
chr5:6880975−6881023:−:ESC
0.0 0.5 1.0 1.5 2.0
RG7_ESC RG7_NSC RG7_NRP iPS_ESC iPS_NSC brain heart kidney
chr6:138526529−138526608:+:ESC
0.0 0.5 1.0 1.5 2.0 2.5
RG7_ESC RG7_NSC RG7_NRP iPS_ESC iPS_NSC brain heart kidney
chr6:73734140−73734186:−:ESC
0.0 0.2 0.4 0.6 0.8
RG7_ESC RG7_NSC RG7_NRP iPS_ESC iPS_NSC brain heart kidney
chrX:150087363−150087446:+:ESC−NS
0 1 2 3 4 5 6
RG7_ESC RG7_NSC RG7_NRP iPS_ESC iPS_NSC brain heart kidney
hsa−miR−1238
0.00 0.01 0.02 0.03 0.04 0.05 0.06
RG7_ESC RG7_NSC RG7_NRP iPS_ESC iPS_NSC brain heart kidney
hsa−miR−1266
0.00 0.02 0.04 0.06 0.08 0.10 0.12
RG7_ESC RG7_NSC RG7_NRP iPS_ESC iPS_NSC brain heart kidney
hsa−miR−1298
0 1 2 3 4
RG7_ESC RG7_NSC RG7_NRP iPS_ESC iPS_NSC brain heart kidney
hsa−miR−182
0 10 20 30 40 50 60 70
RG7_ESC RG7_NSC RG7_NRP iPS_ESC iPS_NSC brain heart kidney
hsa−miR−183
0 5 10 15
RG7_ESC RG7_NSC RG7_NRP iPS_ESC iPS_NSC brain heart kidney
hsa−miR−183star
0.0 0.5 1.0 1.5 2.0
RG7_ESC RG7_NSC RG7_NRP iPS_ESC iPS_NSC brain heart kidney
hsa−miR−187star
0.0 0.2 0.4 0.6 0.8
RG7_ESC RG7_NSC RG7_NRP iPS_ESC iPS_NSC brain heart kidney
hsa−miR−205
0 5 10 15 20 25
RG7_ESC RG7_NSC RG7_NRP iPS_ESC iPS_NSC brain heart kidney
hsa−miR−296−3p
0 2 4 6 8 10
RG7_ESC RG7_NSC RG7_NRP iPS_ESC iPS_NSC brain heart kidney
hsa−miR−302e
0 50 100 150
RG7_ESC RG7_NSC RG7_NRP iPS_ESC iPS_NSC brain heart kidney
hsa−miR−324−3p
0 5 10 15 20
RG7_ESC RG7_NSC RG7_NRP iPS_ESC iPS_NSC brain heart kidney
hsa−miR−517star
0 2 4 6 8 10 12
RG7_ESC RG7_NSC RG7_NRP iPS_ESC iPS_NSC brain heart kidney

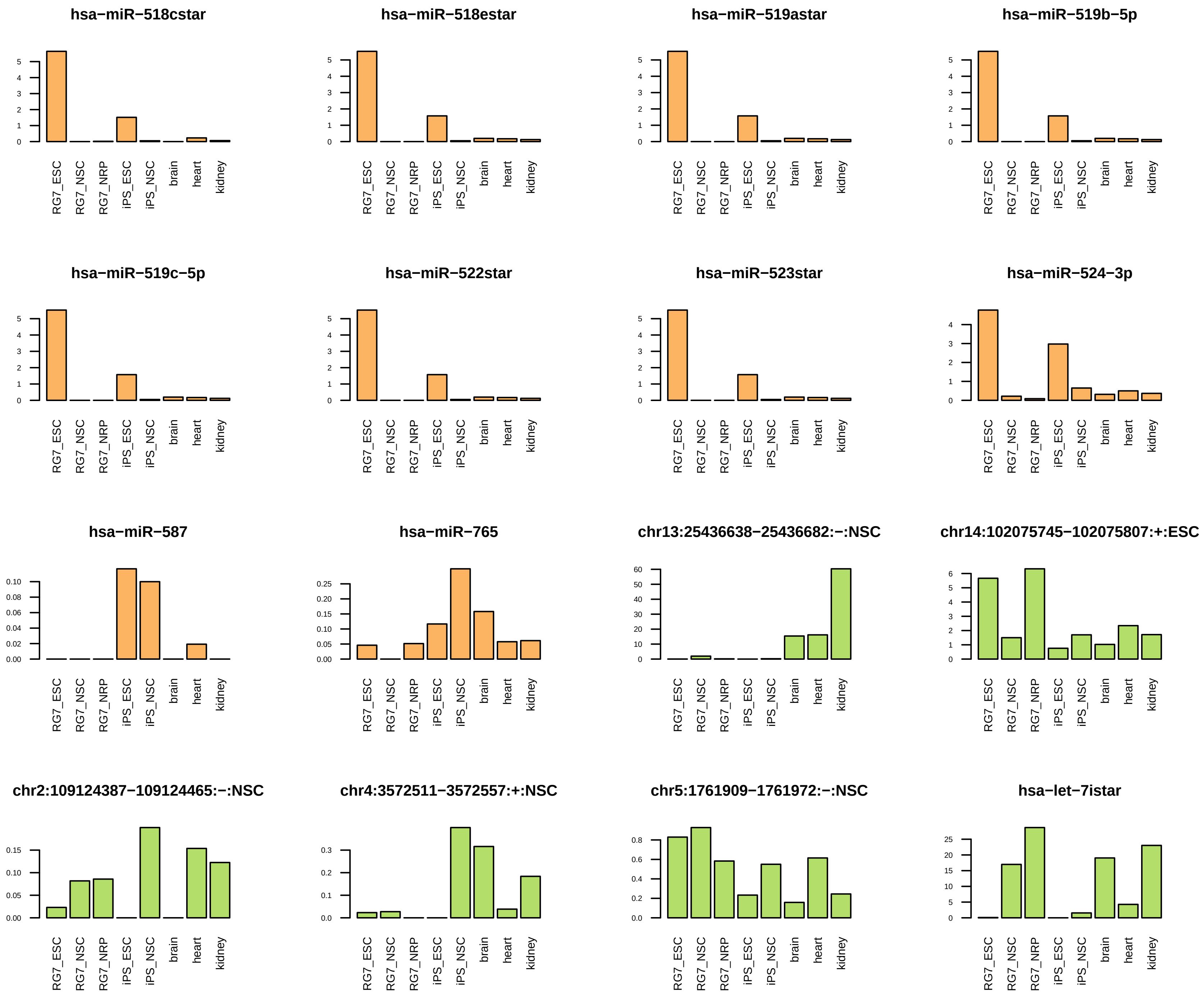
hsa−miR−518cstar
hsa−miR−518estar
hsa−miR−519astar
hsa−miR−519b−5p
hsa−miR−519c−5p
hsa−miR−522star
hsa−miR−523star
hsa−miR−524−3p
hsa−miR−587
hsa−miR−765
chr13:25436638−25436682:−:NSC
chr14:102075745−102075807:+:ESC
chr2:109124387−109124465:−:NSC
chr4:3572511−3572557:+:NSC
chr5:1761909−1761972:−:NSC
hsa−let−7istar
RG7_ESC
RG7_NSC
RG7_NRP
iPS_ESC
iPS_NSC
brain
heart
kidney

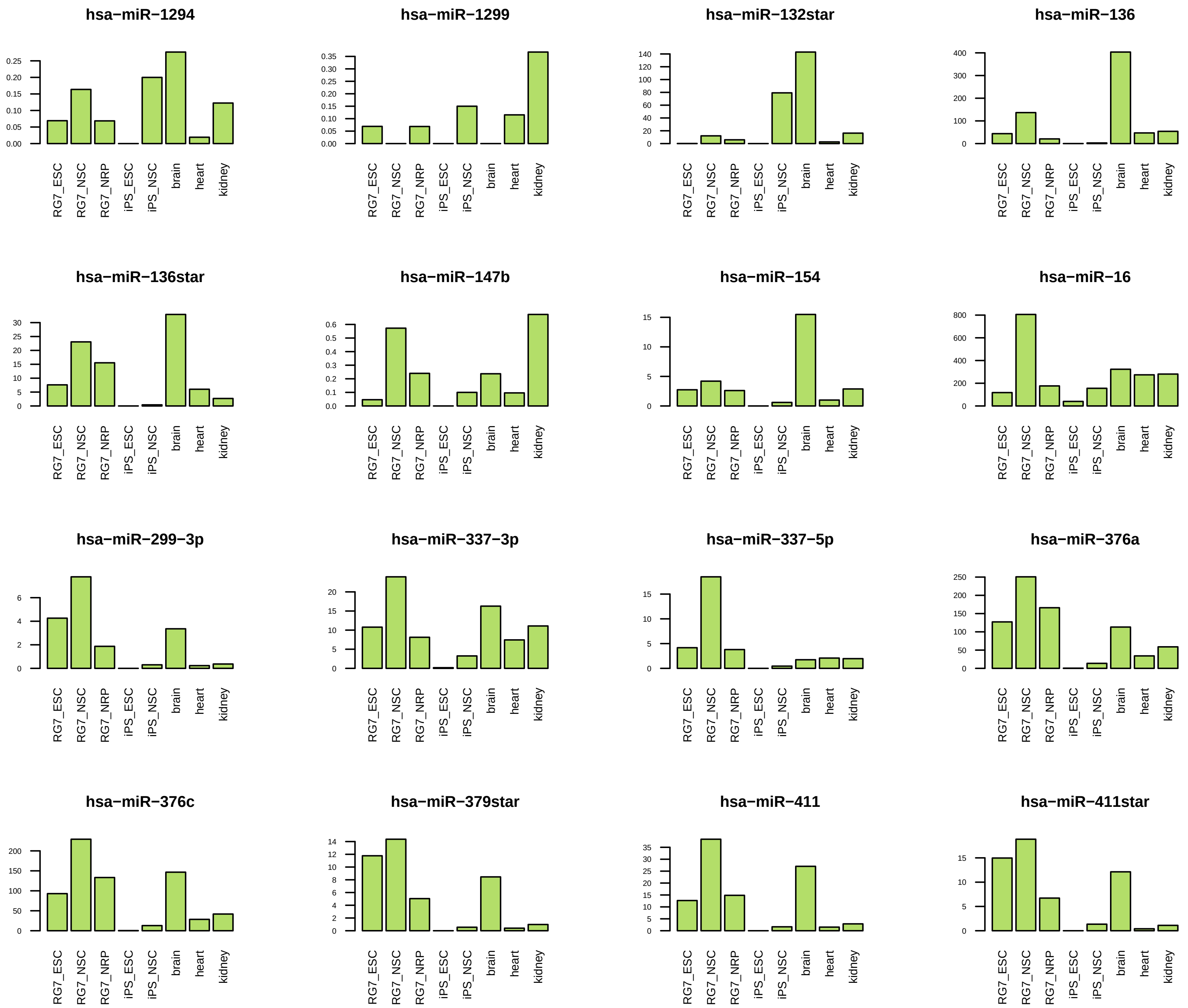
hsa−miR−1294
0.00 0.05 0.10 0.15 0.20 0.25
RG7_ESC RG7_NSC RG7_NRP iPS_ESC iPS_NSC brain heart kidney
hsa−miR−1299
0.00 0.05 0.10 0.15 0.20 0.25 0.30 0.35
RG7_ESC RG7_NSC RG7_NRP iPS_ESC iPS_NSC brain heart kidney
hsa−miR−132star
0 20 40 60 80 100 120 140
RG7_ESC RG7_NSC RG7_NRP iPS_ESC iPS_NSC brain heart kidney
hsa−miR−136
0 100 200 300 400
RG7_ESC RG7_NSC RG7_NRP iPS_ESC iPS_NSC brain heart kidney
hsa−miR−136star
0 5 10 15 20 25 30
RG7_ESC RG7_NSC RG7_NRP iPS_ESC iPS_NSC brain heart kidney
hsa−miR−147b
0.0 0.1 0.2 0.3 0.4 0.5 0.6
RG7_ESC RG7_NSC RG7_NRP iPS_ESC iPS_NSC brain heart kidney
hsa−miR−154
0 5 10 15
RG7_ESC RG7_NSC RG7_NRP iPS_ESC iPS_NSC brain heart kidney
hsa−miR−16
0 200 400 600 800
RG7_ESC RG7_NSC RG7_NRP iPS_ESC iPS_NSC brain heart kidney
hsa−miR−299−3p
0 2 4 6
RG7_ESC RG7_NSC RG7_NRP iPS_ESC iPS_NSC brain heart kidney
hsa−miR−337−3p
0 5 10 15 20
RG7_ESC RG7_NSC RG7_NRP iPS_ESC iPS_NSC brain heart kidney
hsa−miR−337−5p
0 5 10 15
RG7_ESC RG7_NSC RG7_NRP iPS_ESC iPS_NSC brain heart kidney
hsa−miR−376a
0 50 100 150 200 250
RG7_ESC RG7_NSC RG7_NRP iPS_ESC iPS_NSC brain heart kidney
hsa−miR−376c
0 50 100 150 200
RG7_ESC RG7_NSC RG7_NRP iPS_ESC iPS_NSC brain heart kidney
hsa−miR−379star
0 2 4 6 8 10 12 14
RG7_ESC RG7_NSC RG7_NRP iPS_ESC iPS_NSC brain heart kidney
hsa−miR−411
0 5 10 15 20 25 30 35
RG7_ESC RG7_NSC RG7_NRP iPS_ESC iPS_NSC brain heart kidney
hsa−miR−411star
0 5 10 15
RG7_ESC RG7_NSC RG7_NRP iPS_ESC iPS_NSC brain heart kidney

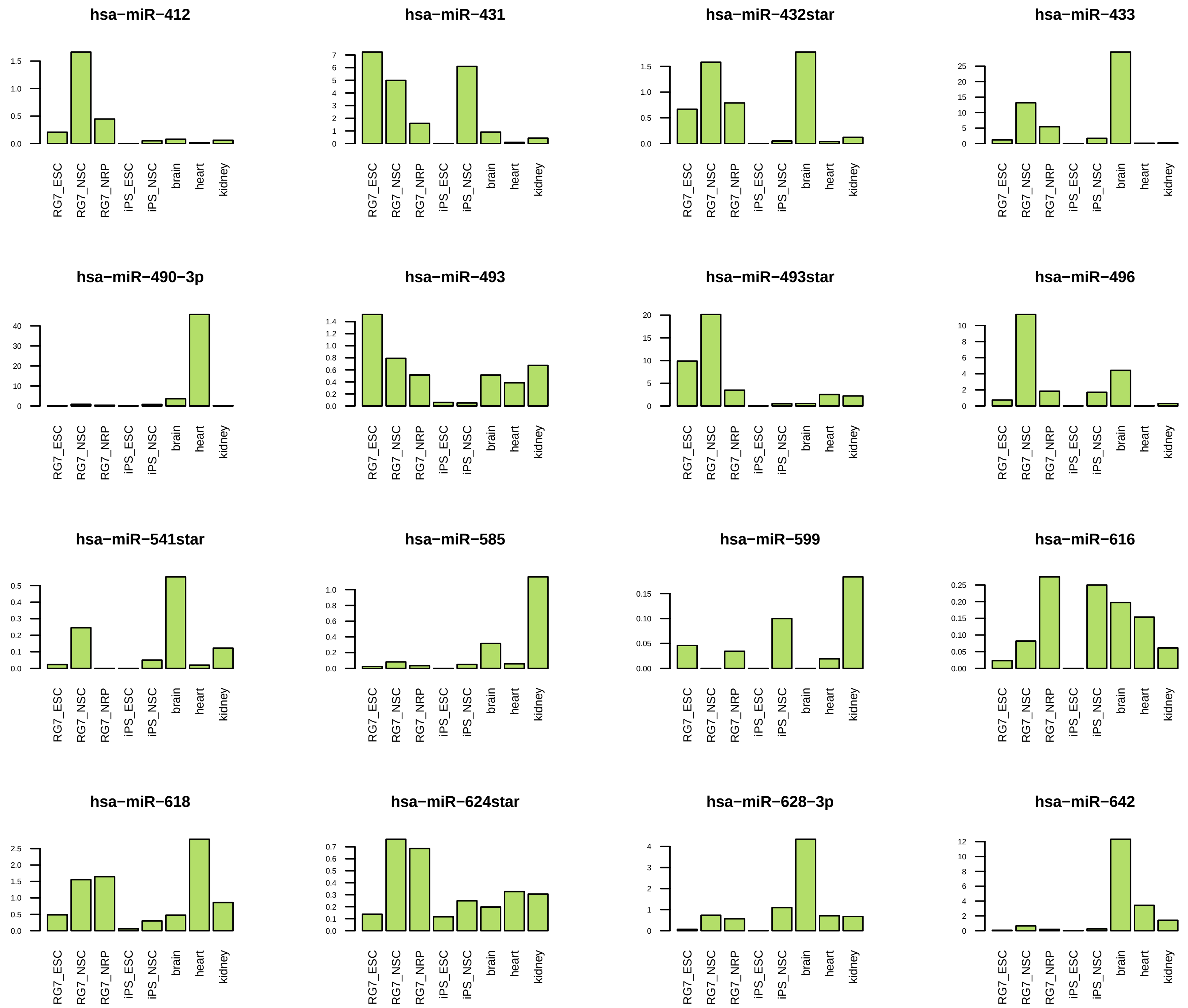
hsa−miR−412
0.0
0.5
1.0
1.5
hsa−miR−431
0
1
2
3
4
5
6
7
hsa−miR−432star
0.0
0.5
1.0
1.5
hsa−miR−433
0
5
10
15
20
25
hsa−miR−490−3p
0
10
20
30
40
hsa−miR−493
0.0
0.2
0.4
0.6
0.8
1.0
1.2
1.4
hsa−miR−493star
0
5
10
15
20
hsa−miR−496
0
2
4
6
8
10
hsa−miR−541star
0.0
0.1
0.2
0.3
0.4
0.5
hsa−miR−585
0.0
0.2
0.4
0.6
0.8
1.0
hsa−miR−599
0.00
0.05
0.10
0.15
hsa−miR−616
0.00
0.05
0.10
0.15
0.20
0.25
hsa−miR−618
0.0
0.5
1.0
1.5
2.0
2.5
hsa−miR−624star
0.0
0.1
0.2
0.3
0.4
0.5
0.6
0.7
hsa−miR−628−3p
0
1
2
3
4
hsa−miR−642
0
2
4
6
8
10
12
RG7_ESC
RG7_NSC
RG7_NRP
iPS_ESC
iPS_NSC
brain
heart
kidney

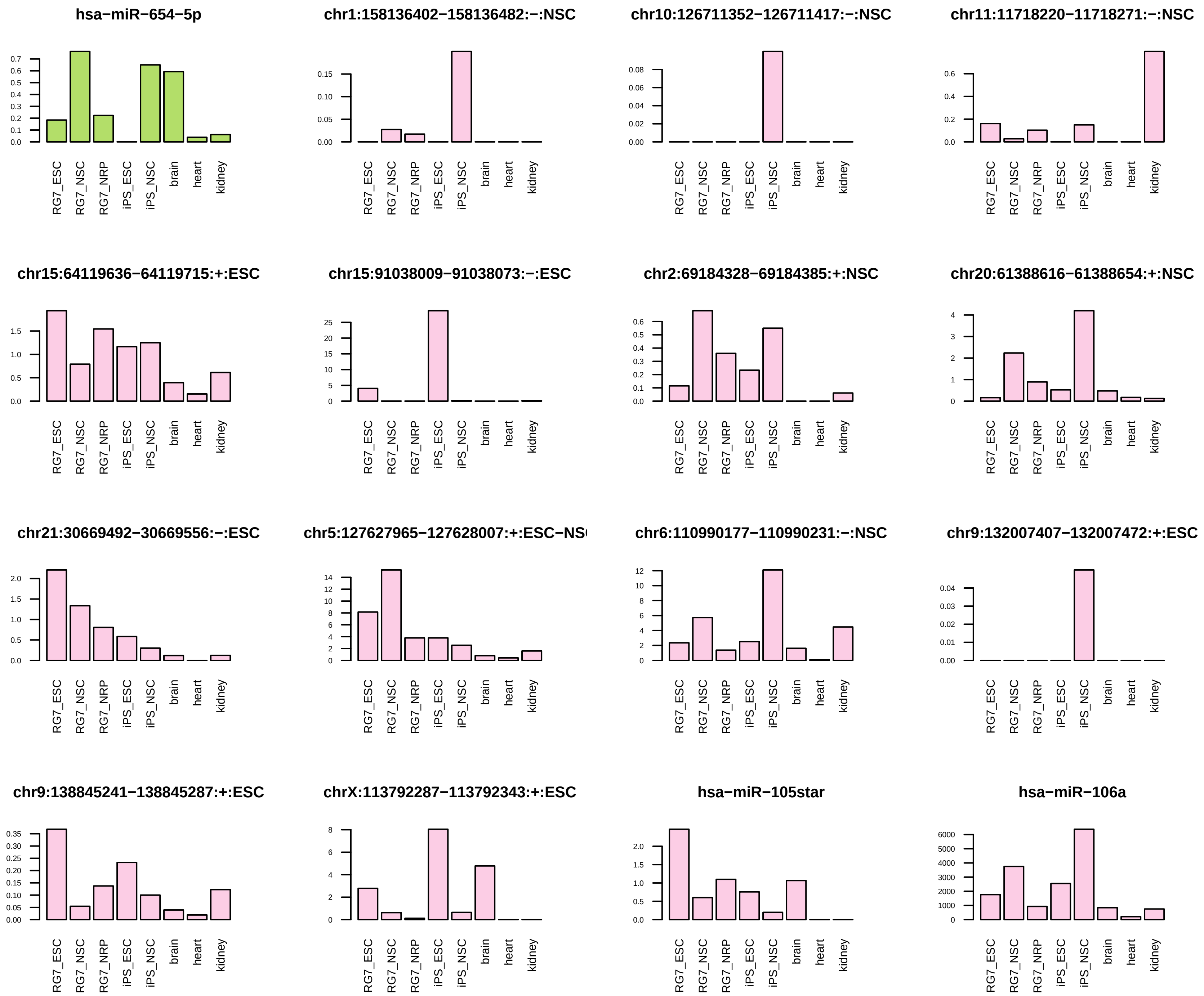
hsa−miR−654−5p
0.0 0.1 0.2 0.3 0.4 0.5 0.6 0.7
chr1:158136402−158136482:−:NSC
0.00 0.05 0.10 0.15
chr10:126711352−126711417:−:NSC
0.00 0.02 0.04 0.06 0.08
chr11:11718220−11718271:−:NSC
0.0 0.2 0.4 0.6
chr15:64119636−64119715:+:ESC
0.0 0.5 1.0 1.5
chr15:91038009−91038073:−:ESC
0 5 10 15 20 25
chr2:69184328−69184385:+:NSC
0.0 0.1 0.2 0.3 0.4 0.5 0.6
chr20:61388616−61388654:+:NSC
0 1 2 3 4
chr21:30669492−30669556:−:ESC
0.0 0.5 1.0 1.5 2.0
chr5:127627965−127628007:+:ESC−NS
0 2 4 6 8 10 12 14
chr6:110990177−110990231:−:NSC
0 2 4 6 8 10 12
chr9:132007407−132007472:+:ESC
0.00 0.01 0.02 0.03 0.04
chr9:138845241−138845287:+:ESC
0.00 0.05 0.10 0.15 0.20 0.25 0.30 0.35
chrX:113792287−113792343:+:ESC
0 2 4 6 8
hsa−miR−105star
0.0 0.5 1.0 1.5 2.0
hsa−miR−106a
0 1000 2000 3000 4000 5000 6000
RG7_ESC
RG7_NSC
RG7_NRP
iPS_ESC
iPS_NSC
brain
heart
kidney

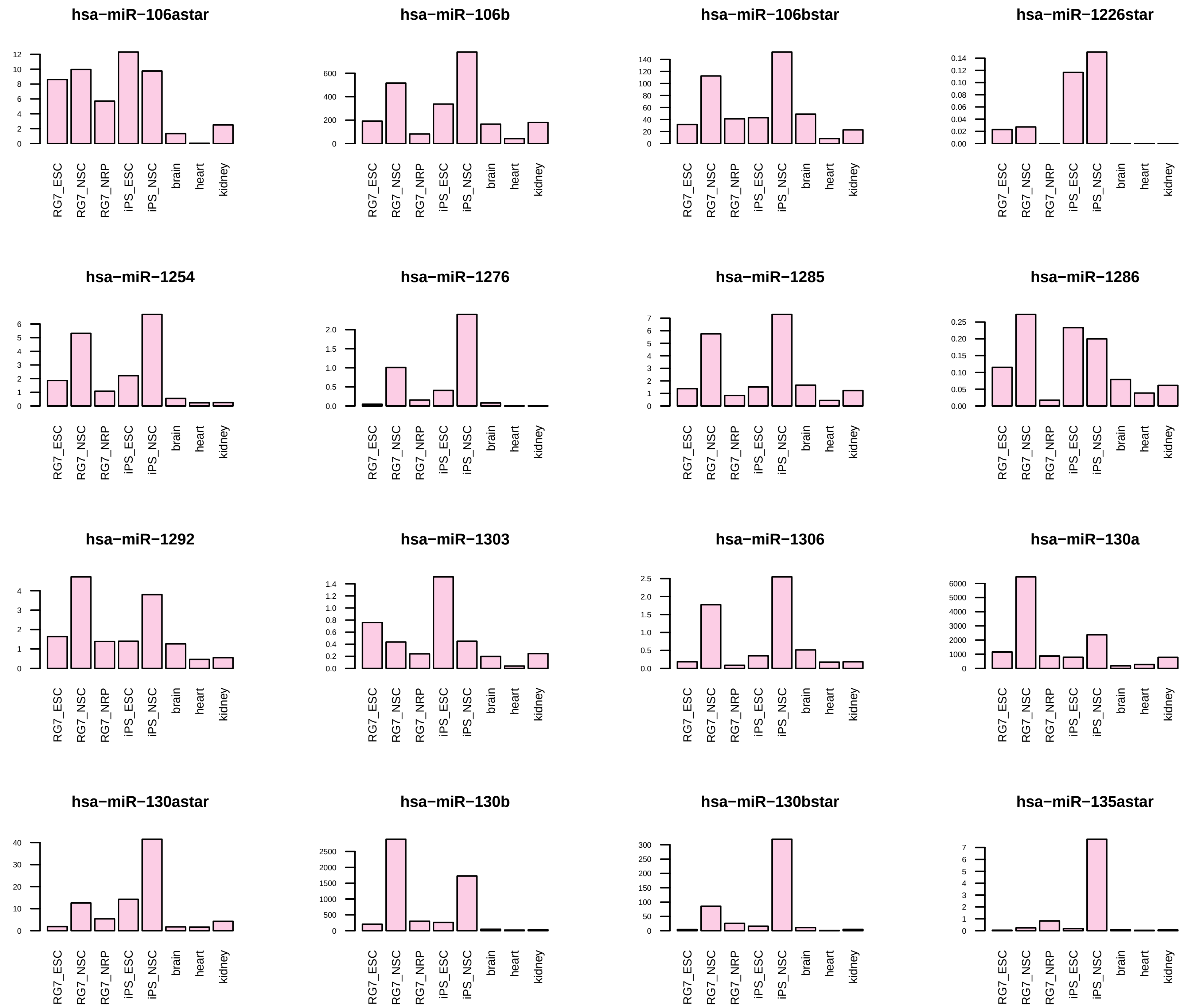
hsa−miR−106astar
hsa−miR−106b
hsa−miR−106bstar
hsa−miR−1226star
hsa−miR−1254
hsa−miR−1276
hsa−miR−1285
hsa−miR−1286
hsa−miR−1292
hsa−miR−1303
hsa−miR−1306
hsa−miR−130a
hsa−miR−130astar
hsa−miR−130b
hsa−miR−130bstar
hsa−miR−135astar
RG7_ESC
RG7_NSC
RG7_NRP
iPS_ESC
iPS_NSC
brain
heart
kidney

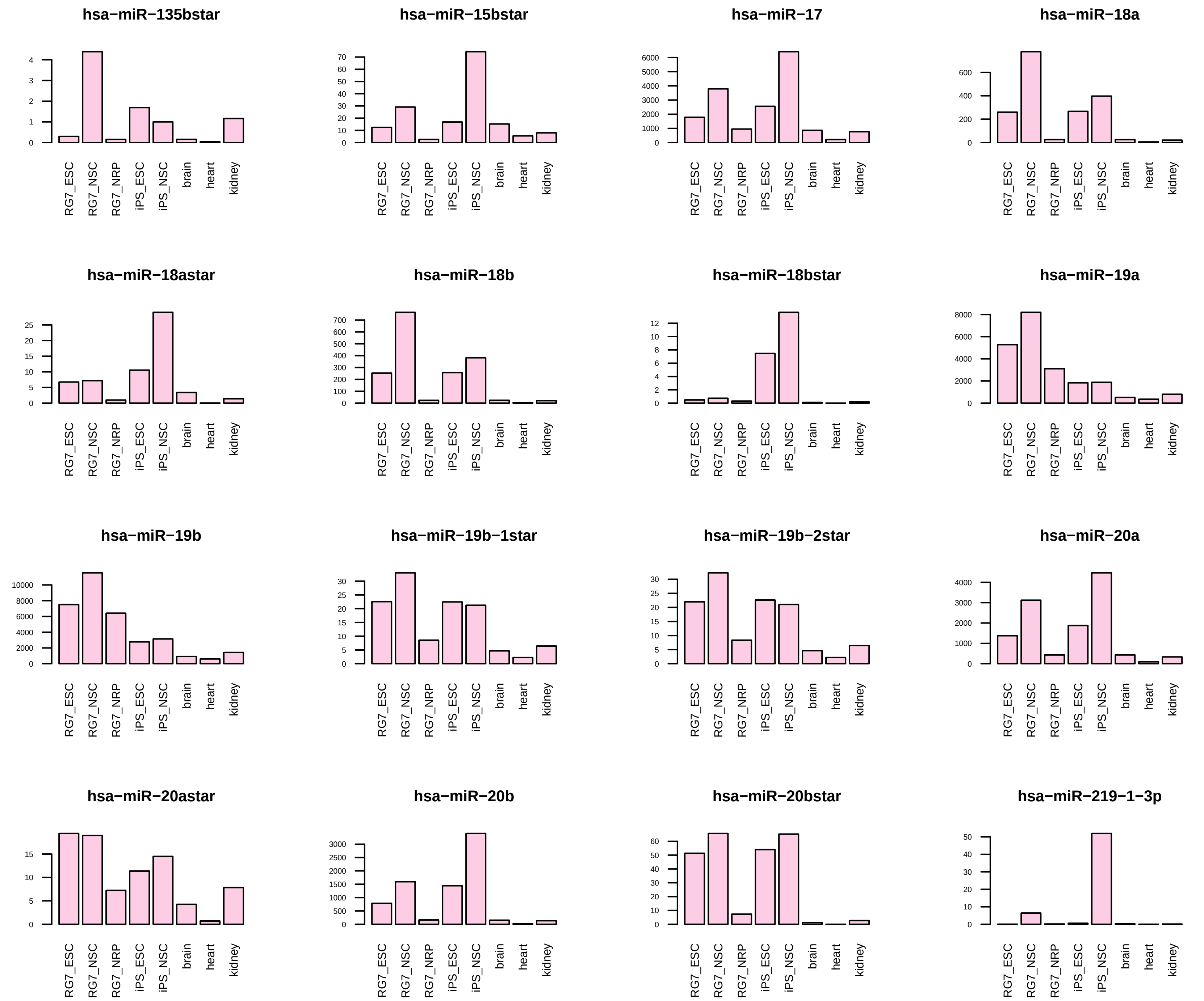
hsa−miR−135bstar
0
1
2
3
4
hsa−miR−15bstar
0
10
20
30
40
50
60
70
hsa−miR−17
0
1000
2000
3000
4000
5000
6000
hsa−miR−18a
0
200
400
600
hsa−miR−18astar
0
5
10
15
20
25
hsa−miR−18b
0
100
200
300
400
500
600
700
hsa−miR−18bstar
0
2
4
6
8
10
12
hsa−miR−19a
0
2000
4000
6000
8000
hsa−miR−19b
0
2000
4000
6000
8000
10000
hsa−miR−19b−1star
0
5
10
15
20
25
30
hsa−miR−19b−2star
0
5
10
15
20
25
30
hsa−miR−20a
0
1000
2000
3000
4000
hsa−miR−20astar
0
5
10
15
hsa−miR−20b
0
500
1000
1500
2000
2500
3000
hsa−miR−20bstar
0
10
20
30
40
50
60
hsa−miR−219−1−3p
0
10
20
30
40
50
RG7_ESC
RG7_NSC
RG7_NRP
iPS_ESC
iPS_NSC
brain
heart
kidney

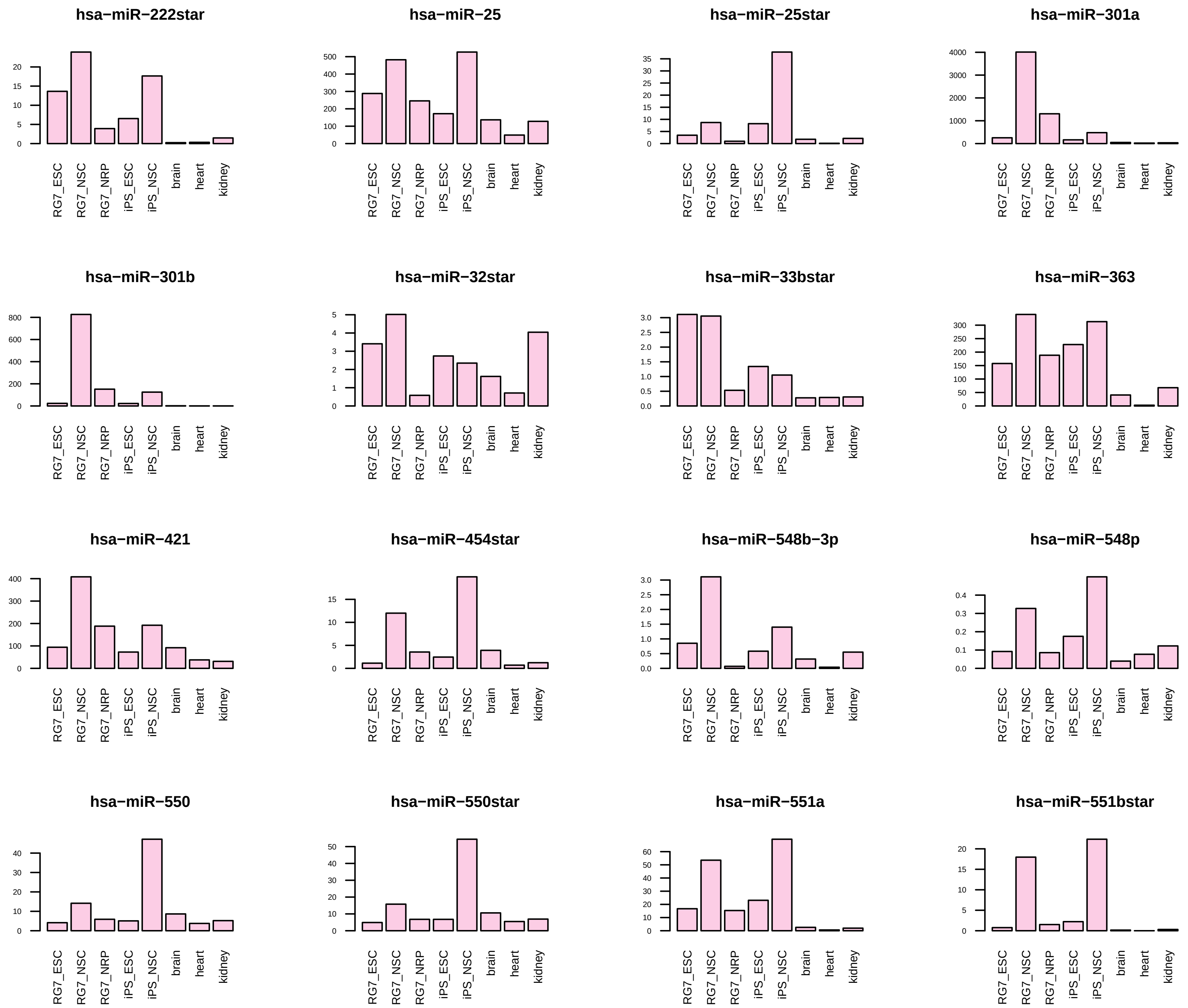

hsa−miR−222star
0 5 10 15 20
hsa−miR−25
0 100 200 300 400 500
hsa−miR−25star
0 5 10 15 20 25 30 35
hsa−miR−301a
0 1000 2000 3000 4000
hsa−miR−301b
0 200 400 600 800
hsa−miR−32star
0 1 2 3 4 5
hsa−miR−33bstar
0.0 0.5 1.0 1.5 2.0 2.5 3.0
hsa−miR−363
0 50 100 150 200 250 300
hsa−miR−421
0 100 200 300 400
hsa−miR−454star
0 5 10 15
hsa−miR−548b−3p
0.0 0.5 1.0 1.5 2.0 2.5 3.0
hsa−miR−548p
0.0 0.1 0.2 0.3 0.4
hsa−miR−550
0 10 20 30 40
hsa−miR−550star
0 10 20 30 40 50
hsa−miR−551a
0 10 20 30 40 50 60
hsa−miR−551bstar
0 5 10 15 20
RG7_ESC
RG7_NSC
RG7_NRP
iPS_ESC
iPS_NSC
brain
heart
kidney

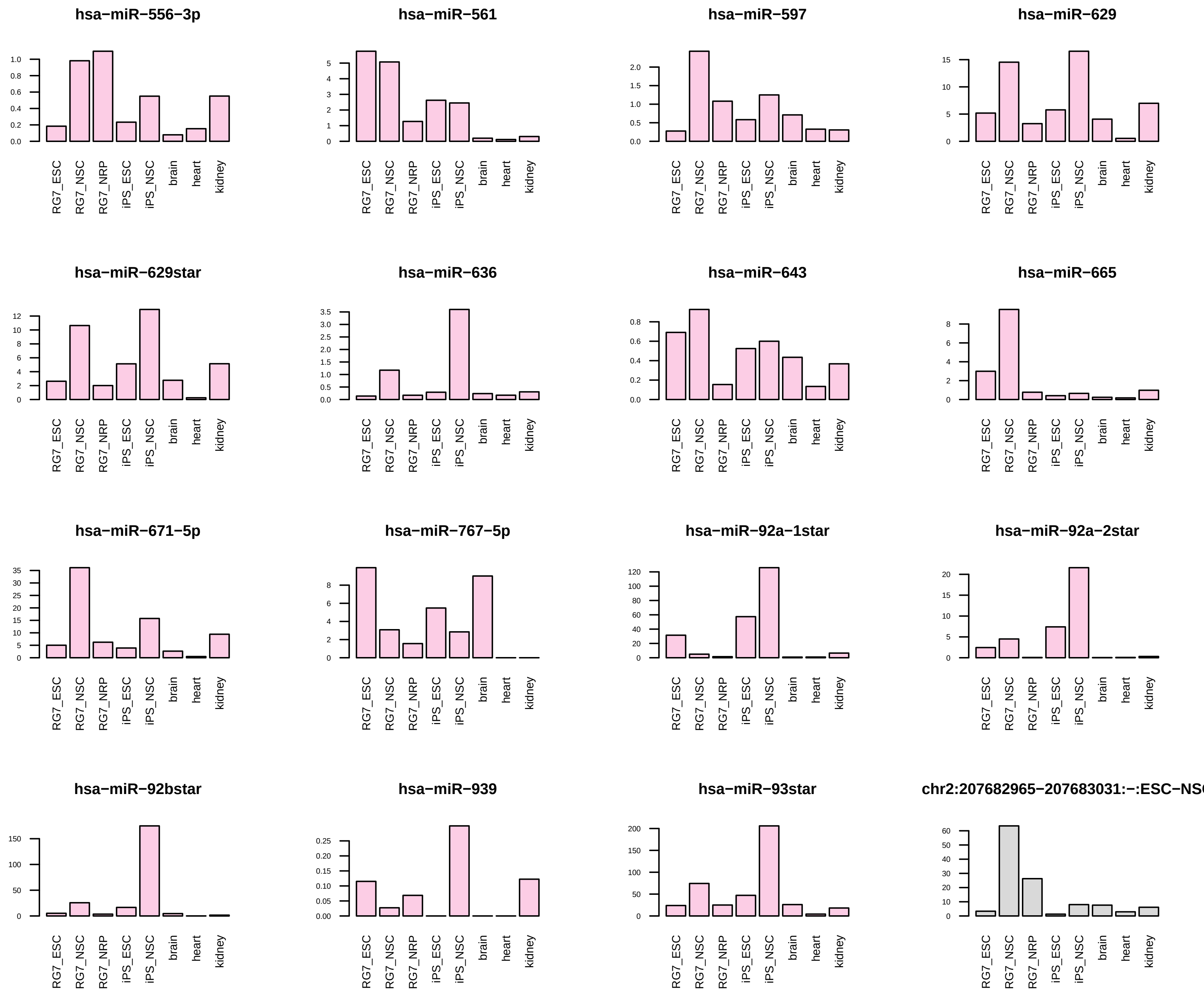

hsa−miR−556−3p
hsa−miR−561
hsa−miR−597
hsa−miR−629
hsa−miR−629star
hsa−miR−636
hsa−miR−643
hsa−miR−665
hsa−miR−671−5p
hsa−miR−767−5p
hsa−miR−92a−1star
hsa−miR−92a−2star
hsa−miR−92bstar
hsa−miR−939
hsa−miR−93star
chr2:207682965−207683031:−:ESC−NS
RG7_ESC
RG7_NSC
RG7_NRP
iPS_ESC
iPS_NSC
brain
heart
kidney

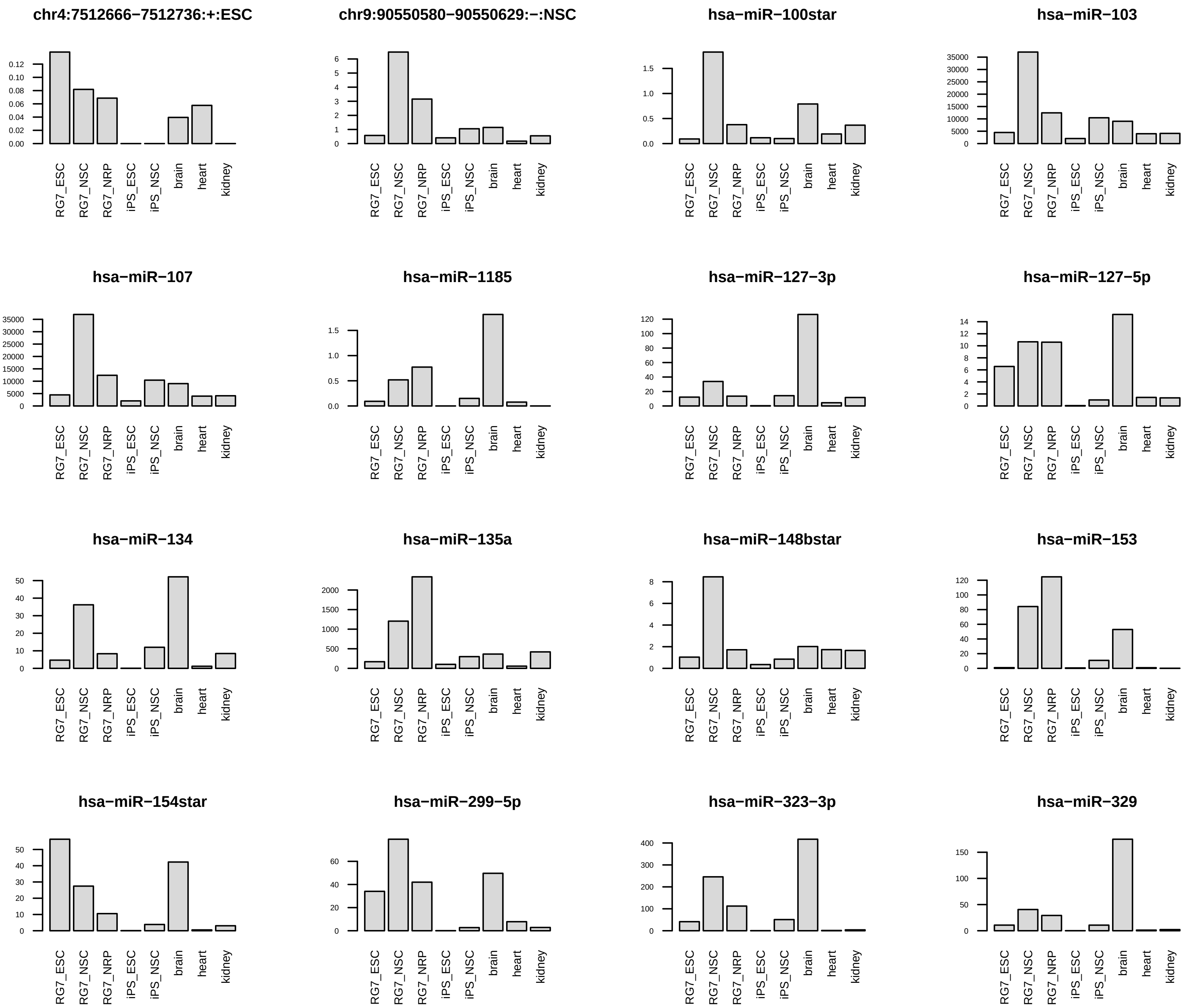
chr4:7512666−7512736:+:ESC
chr9:90550580−90550629:−:NSC
hsa−miR−100star
hsa−miR−103
hsa−miR−107
hsa−miR−1185
hsa−miR−127−3p
hsa−miR−127−5p
hsa−miR−134
hsa−miR−135a
hsa−miR−148bstar
hsa−miR−153
hsa−miR−154star
hsa−miR−299−5p
hsa−miR−323−3p
hsa−miR−329
RG7_ESC
RG7_NSC
RG7_NRP
iPS_ESC
iPS_NSC
brain
heart
kidney

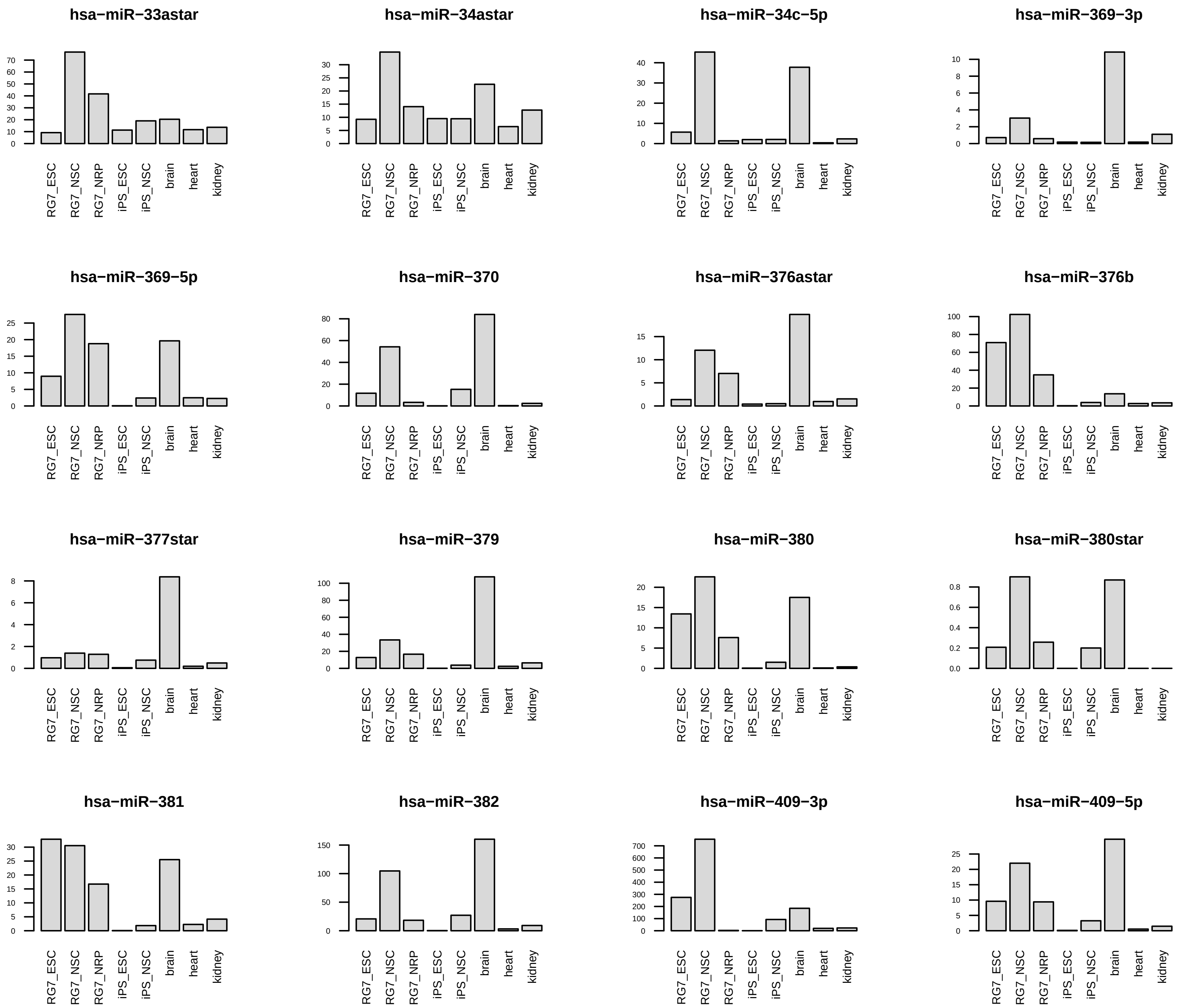
hsa−miR−33astar
0 10 20 30 40 50 60 70
RG7_ESC RG7_NSC RG7_NRP iPS_ESC iPS_NSC brain heart kidney
hsa−miR−34astar
0 5 10 15 20 25 30
RG7_ESC RG7_NSC RG7_NRP iPS_ESC iPS_NSC brain heart kidney
hsa−miR−34c−5p
0 10 20 30 40
RG7_ESC RG7_NSC RG7_NRP iPS_ESC iPS_NSC brain heart kidney
hsa−miR−369−3p
0 2 4 6 8 10
RG7_ESC RG7_NSC RG7_NRP iPS_ESC iPS_NSC brain heart kidney
hsa−miR−369−5p
0 5 10 15 20 25
RG7_ESC RG7_NSC RG7_NRP iPS_ESC iPS_NSC brain heart kidney
hsa−miR−370
0 20 40 60 80
RG7_ESC RG7_NSC RG7_NRP iPS_ESC iPS_NSC brain heart kidney
hsa−miR−376astar
0 5 10 15
RG7_ESC RG7_NSC RG7_NRP iPS_ESC iPS_NSC brain heart kidney
hsa−miR−376b
0 20 40 60 80 100
RG7_ESC RG7_NSC RG7_NRP iPS_ESC iPS_NSC brain heart kidney
hsa−miR−377star
0 2 4 6 8
RG7_ESC RG7_NSC RG7_NRP iPS_ESC iPS_NSC brain heart kidney
hsa−miR−379
0 20 40 60 80 100
RG7_ESC RG7_NSC RG7_NRP iPS_ESC iPS_NSC brain heart kidney
hsa−miR−380
0 5 10 15 20
RG7_ESC RG7_NSC RG7_NRP iPS_ESC iPS_NSC brain heart kidney
hsa−miR−380star
0.0 0.2 0.4 0.6 0.8
RG7_ESC RG7_NSC RG7_NRP iPS_ESC iPS_NSC brain heart kidney
hsa−miR−381
0 5 10 15 20 25 30
RG7_ESC RG7_NSC RG7_NRP iPS_ESC iPS_NSC brain heart kidney
hsa−miR−382
0 50 100 150
RG7_ESC RG7_NSC RG7_NRP iPS_ESC iPS_NSC brain heart kidney
hsa−miR−409−3p
0 100 200 300 400 500 600 700
RG7_ESC RG7_NSC RG7_NRP iPS_ESC iPS_NSC brain heart kidney
hsa−miR−409−5p
0 5 10 15 20 25
RG7_ESC RG7_NSC RG7_NRP iPS_ESC iPS_NSC brain heart kidney

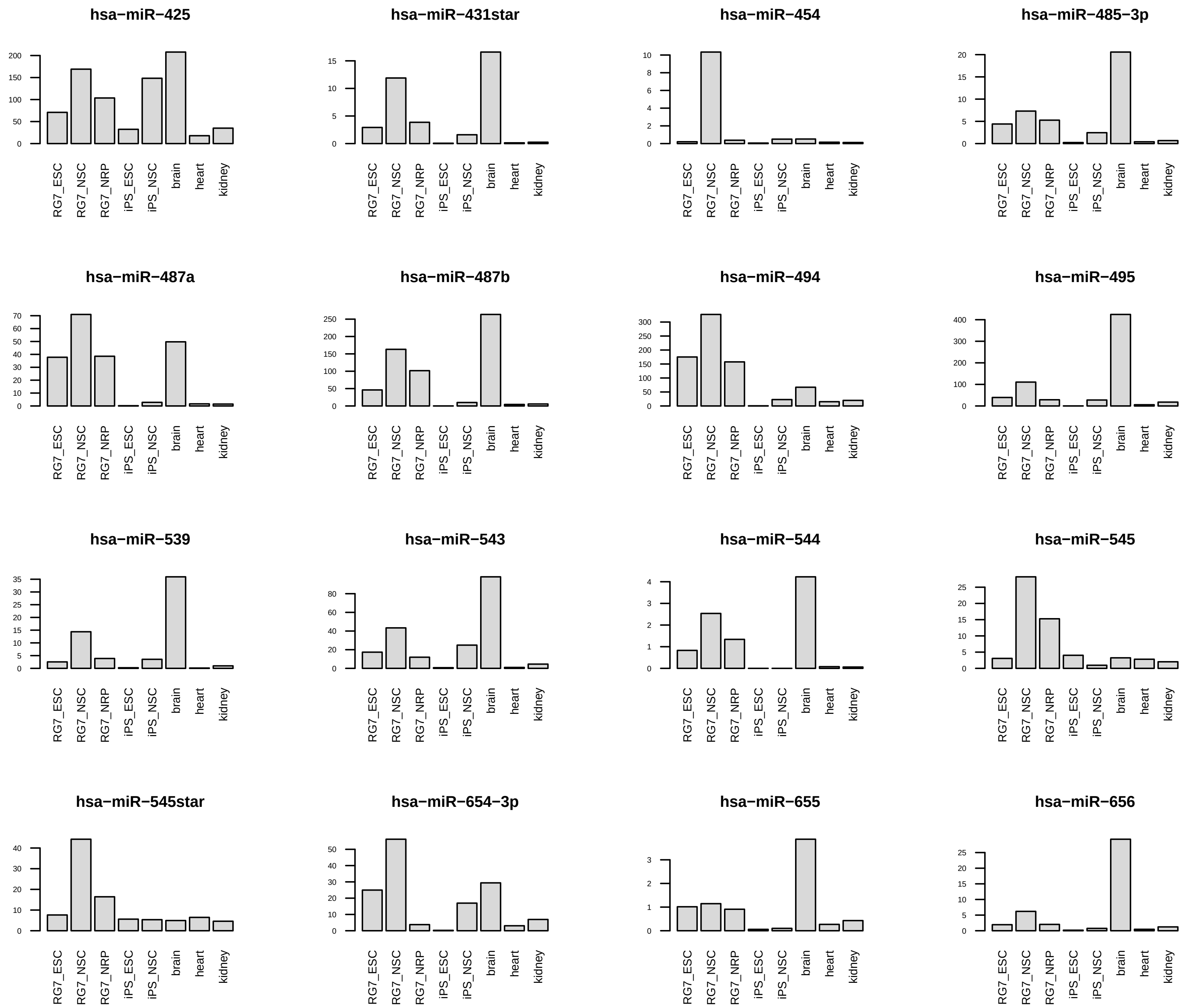

hsa−miR−425
0
50
100
150
200
RG7_ESC
RG7_NSC
RG7_NRP
iPS_ESC
iPS_NSC
brain
heart
kidney
hsa−miR−431star
0
5
10
15
hsa−miR−454
0
2
4
6
8
10
hsa−miR−485−3p
0
5
10
15
20
hsa−miR−487a
0
10
20
30
40
50
60
70
hsa−miR−487b
0
50
100
150
200
250
hsa−miR−494
0
50
100
150
200
250
300
hsa−miR−495
0
100
200
300
400
hsa−miR−539
0
5
10
15
20
25
30
35
hsa−miR−543
0
20
40
60
80
hsa−miR−544
0
1
2
3
4
hsa−miR−545
0
5
10
15
20
25
hsa−miR−545star
0
10
20
30
40
hsa−miR−654−3p
0
10
20
30
40
50
hsa−miR−655
0
1
2
3
hsa−miR−656
0
5
10
15
20
25

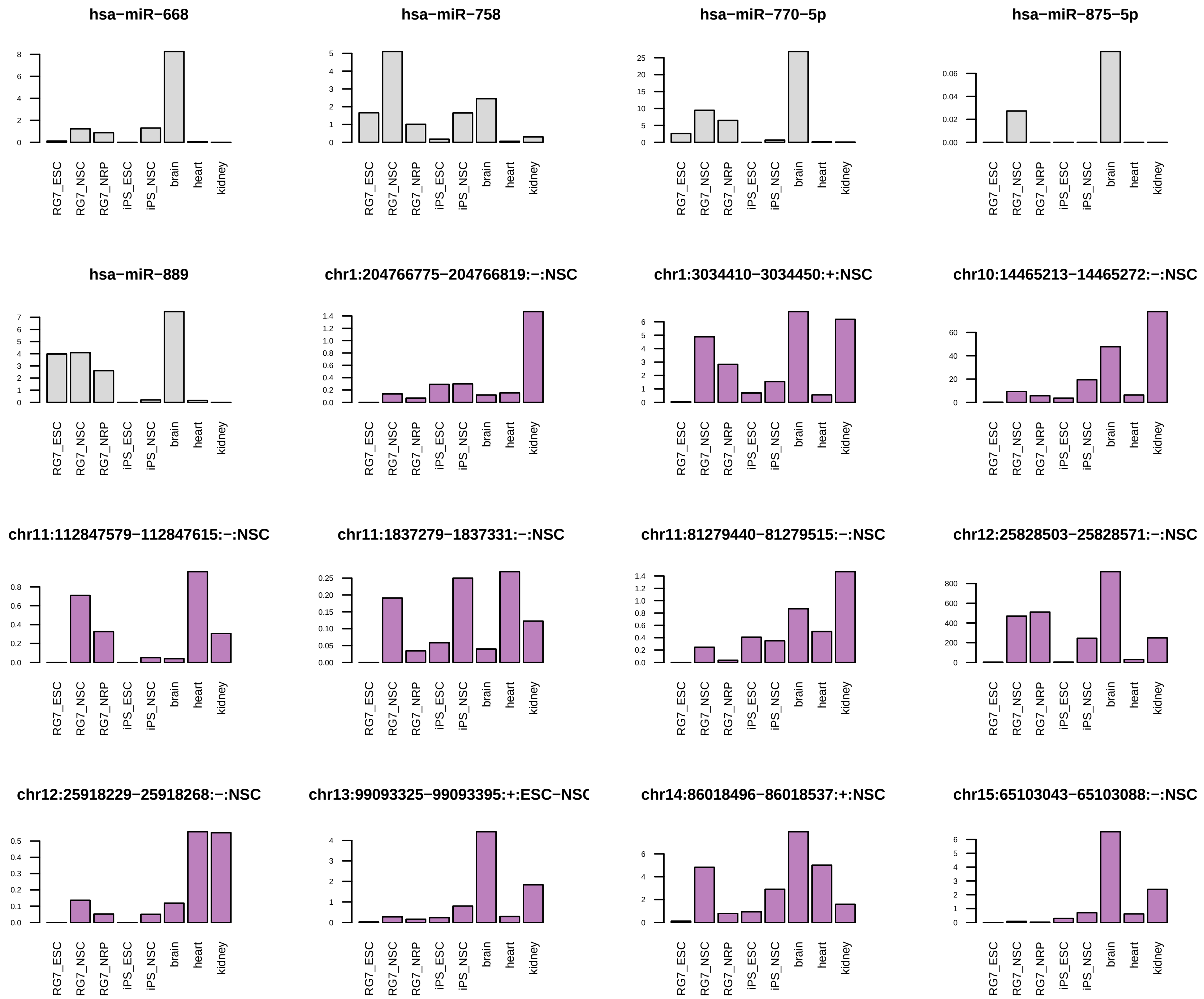
hsa−miR−668
0 2 4 6 8
hsa−miR−758
0 1 2 3 4 5
hsa−miR−770−5p
0 5 10 15 20 25
hsa−miR−875−5p
0.00 0.02 0.04 0.06
hsa−miR−889
0 1 2 3 4 5 6 7
chr1:204766775−204766819:−:NSC
0.0 0.2 0.4 0.6 0.8 1.0 1.2 1.4
chr1:3034410−3034450:+:NSC
0 1 2 3 4 5 6
chr10:14465213−14465272:−:NSC
0 20 40 60
chr11:112847579−112847615:−:NSC
0.0 0.2 0.4 0.6 0.8
chr11:1837279−1837331:−:NSC
0.00 0.05 0.10 0.15 0.20 0.25
chr11:81279440−81279515:−:NSC
0.0 0.2 0.4 0.6 0.8 1.0 1.2 1.4
chr12:25828503−25828571:−:NSC
0 200 400 600 800
chr12:25918229−25918268:−:NSC
0.0 0.1 0.2 0.3 0.4 0.5
chr13:99093325−99093395:+:ESC−NSC
0 1 2 3 4
chr14:86018496−86018537:+:NSC
0 2 4 6
chr15:65103043−65103088:−:NSC
0 1 2 3 4 5 6
RG7_ESC
RG7_NSC
RG7_NRP
iPS_ESC
iPS_NSC
brain
heart
kidney

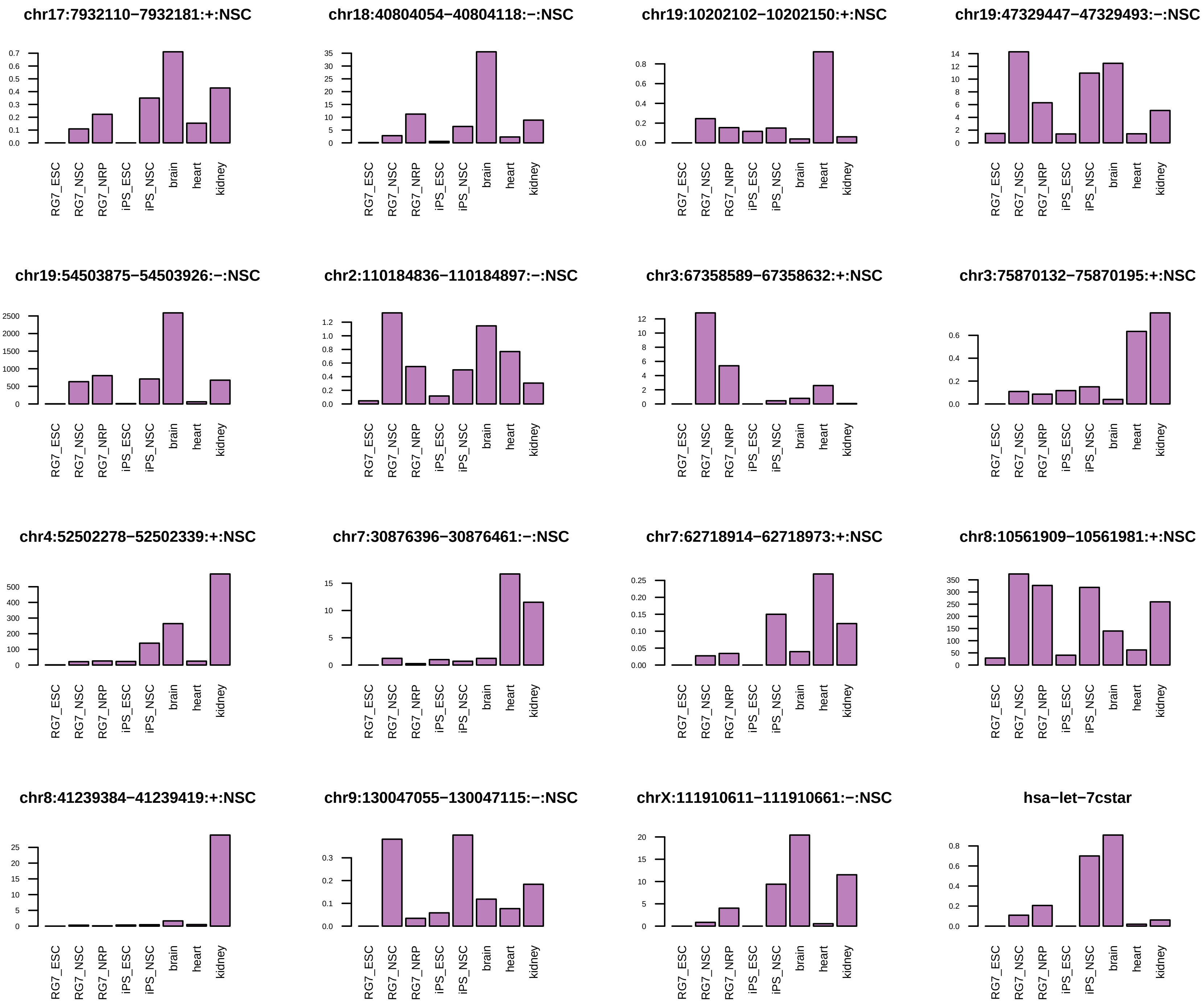
chr17:7932110−7932181:+:NSC
0.0 0.1 0.2 0.3 0.4 0.5 0.6 0.7
RG7_ESC RG7_NSC RG7_NRP iPS_ESC iPS_NSC brain heart kidney
chr18:40804054−40804118:−:NSC
0 5 10 15 20 25 30 35
RG7_ESC RG7_NSC RG7_NRP iPS_ESC iPS_NSC brain heart kidney
chr19:10202102−10202150:+:NSC
0.0 0.2 0.4 0.6 0.8
RG7_ESC RG7_NSC RG7_NRP iPS_ESC iPS_NSC brain heart kidney
chr19:47329447−47329493:−:NSC
0 2 4 6 8 10 12 14
RG7_ESC RG7_NSC RG7_NRP iPS_ESC iPS_NSC brain heart kidney
chr19:54503875−54503926:−:NSC
0 500 1000 1500 2000 2500
RG7_ESC RG7_NSC RG7_NRP iPS_ESC iPS_NSC brain heart kidney
chr2:110184836−110184897:−:NSC
0.0 0.2 0.4 0.6 0.8 1.0 1.2
RG7_ESC RG7_NSC RG7_NRP iPS_ESC iPS_NSC brain heart kidney
chr3:67358589−67358632:+:NSC
0 2 4 6 8 10 12
RG7_ESC RG7_NSC RG7_NRP iPS_ESC iPS_NSC brain heart kidney
chr3:75870132−75870195:+:NSC
0.0 0.2 0.4 0.6
RG7_ESC RG7_NSC RG7_NRP iPS_ESC iPS_NSC brain heart kidney
chr4:52502278−52502339:+:NSC
0 100 200 300 400 500
RG7_ESC RG7_NSC RG7_NRP iPS_ESC iPS_NSC brain heart kidney
chr7:30876396−30876461:−:NSC
0 5 10 15
RG7_ESC RG7_NSC RG7_NRP iPS_ESC iPS_NSC brain heart kidney
chr7:62718914−62718973:+:NSC
0.00 0.05 0.10 0.15 0.20 0.25
RG7_ESC RG7_NSC RG7_NRP iPS_ESC iPS_NSC brain heart kidney
chr8:10561909−10561981:+:NSC
0 50 100 150 200 250 300 350
RG7_ESC RG7_NSC RG7_NRP iPS_ESC iPS_NSC brain heart kidney
chr8:41239384−41239419:+:NSC
0 5 10 15 20 25
RG7_ESC RG7_NSC RG7_NRP iPS_ESC iPS_NSC brain heart kidney
chr9:130047055−130047115:−:NSC
0.0 0.1 0.2 0.3
RG7_ESC RG7_NSC RG7_NRP iPS_ESC iPS_NSC brain heart kidney
chrX:111910611−111910661:−:NSC
0 5 10 15 20
RG7_ESC RG7_NSC RG7_NRP iPS_ESC iPS_NSC brain heart kidney
hsa−let−7cstar
0.0 0.2 0.4 0.6 0.8
RG7_ESC RG7_NSC RG7_NRP iPS_ESC iPS_NSC brain heart kidney

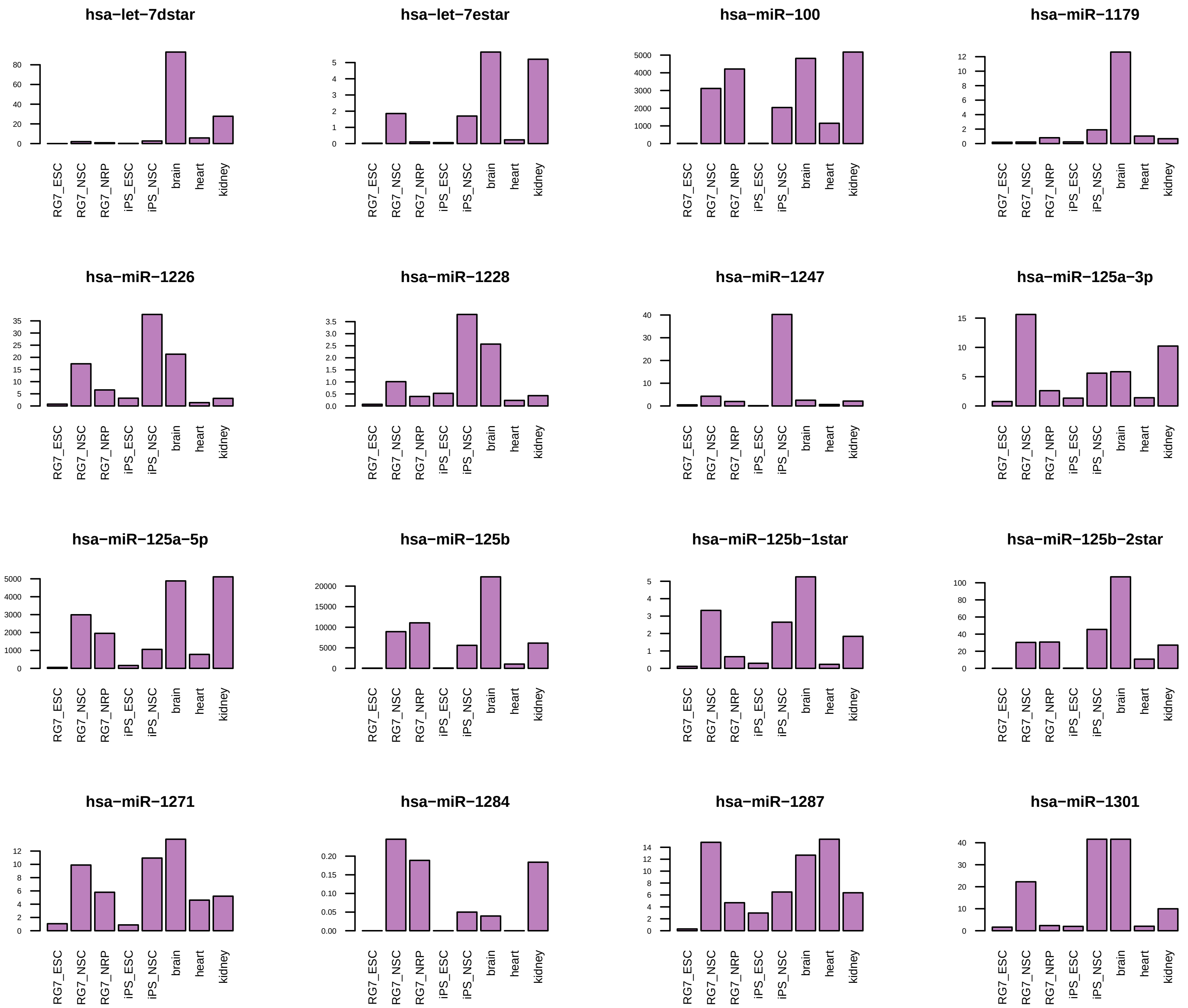
hsa−let−7dstar
0 20 40 60 80
hsa−let−7estar
0 1 2 3 4 5
hsa−miR−100
0 1000 2000 3000 4000 5000
hsa−miR−1179
0 2 4 6 8 10 12
hsa−miR−1226
0 5 10 15 20 25 30 35
hsa−miR−1228
0.0 0.5 1.0 1.5 2.0 2.5 3.0 3.5
hsa−miR−1247
0 10 20 30 40
hsa−miR−125a−3p
0 5 10 15
hsa−miR−125a−5p
0 1000 2000 3000 4000 5000
hsa−miR−125b
0 5000 10000 15000 20000
hsa−miR−125b−1star
0 1 2 3 4 5
hsa−miR−125b−2star
0 20 40 60 80 100
hsa−miR−1271
0 2 4 6 8 10 12
hsa−miR−1284
0.00 0.05 0.10 0.15 0.20
hsa−miR−1287
0 2 4 6 8 10 12 14
hsa−miR−1301
0 10 20 30 40
RG7_ESC
RG7_NSC
RG7_NRP
iPS_ESC
iPS_NSC
brain
heart
kidney

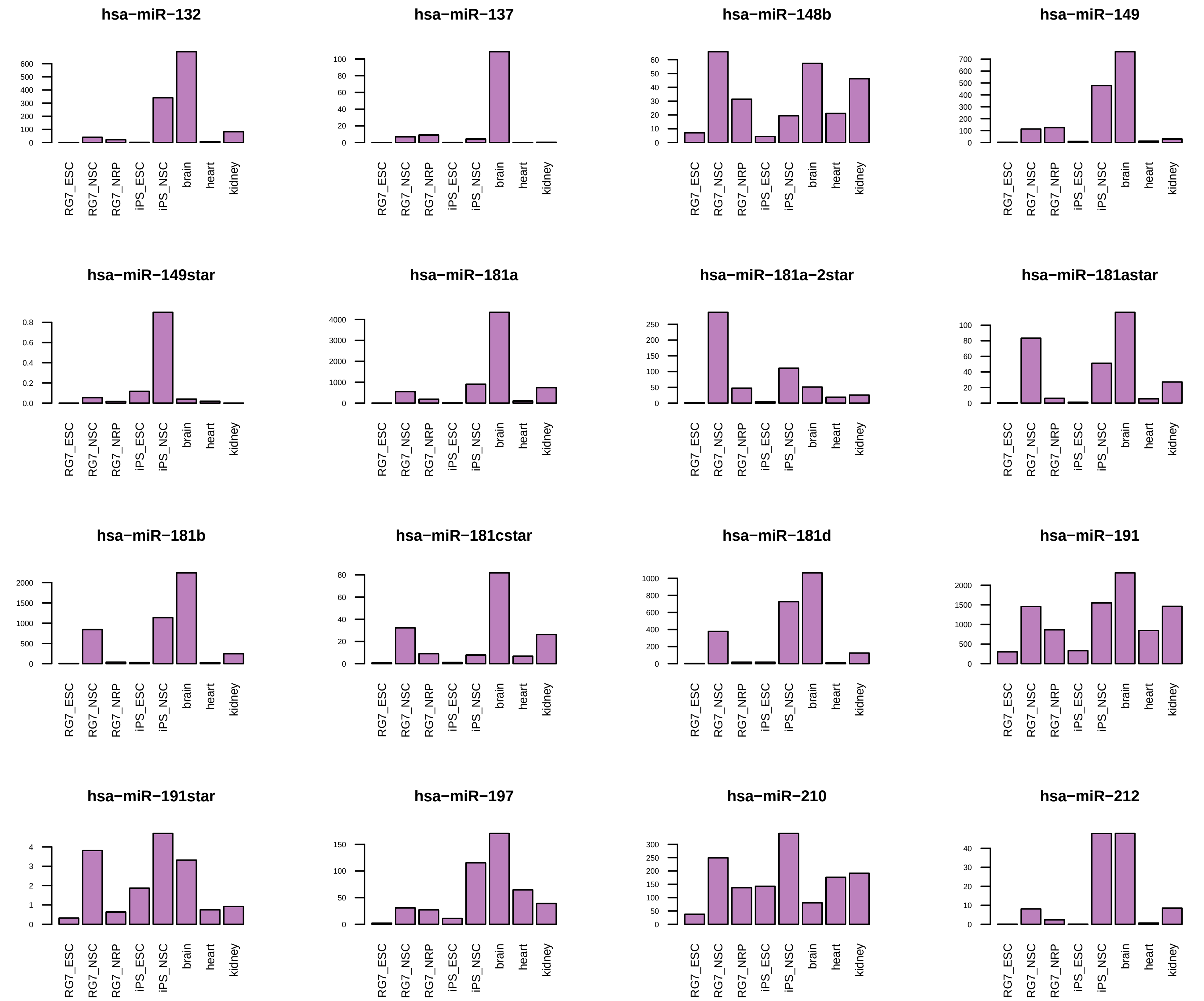

hsa−miR−132
0
100
200
300
400
500
600
hsa−miR−137
0
20
40
60
80
100
hsa−miR−148b
0
10
20
30
40
50
60
hsa−miR−149
0
100
200
300
400
500
600
700
hsa−miR−149star
0.0
0.2
0.4
0.6
0.8
hsa−miR−181a
0
1000
2000
3000
4000
hsa−miR−181a−2star
0
50
100
150
200
250
hsa−miR−181astar
0
20
40
60
80
100
hsa−miR−181b
0
500
1000
1500
2000
hsa−miR−181cstar
0
20
40
60
80
hsa−miR−181d
0
200
400
600
800
1000
hsa−miR−191
0
500
1000
1500
2000
hsa−miR−191star
0
1
2
3
4
hsa−miR−197
0
50
100
150
hsa−miR−210
0
50
100
150
200
250
300
hsa−miR−212
0
10
20
30
40
RG7_ESC
RG7_NSC
RG7_NRP
iPS_ESC
iPS_NSC
brain
heart
kidney

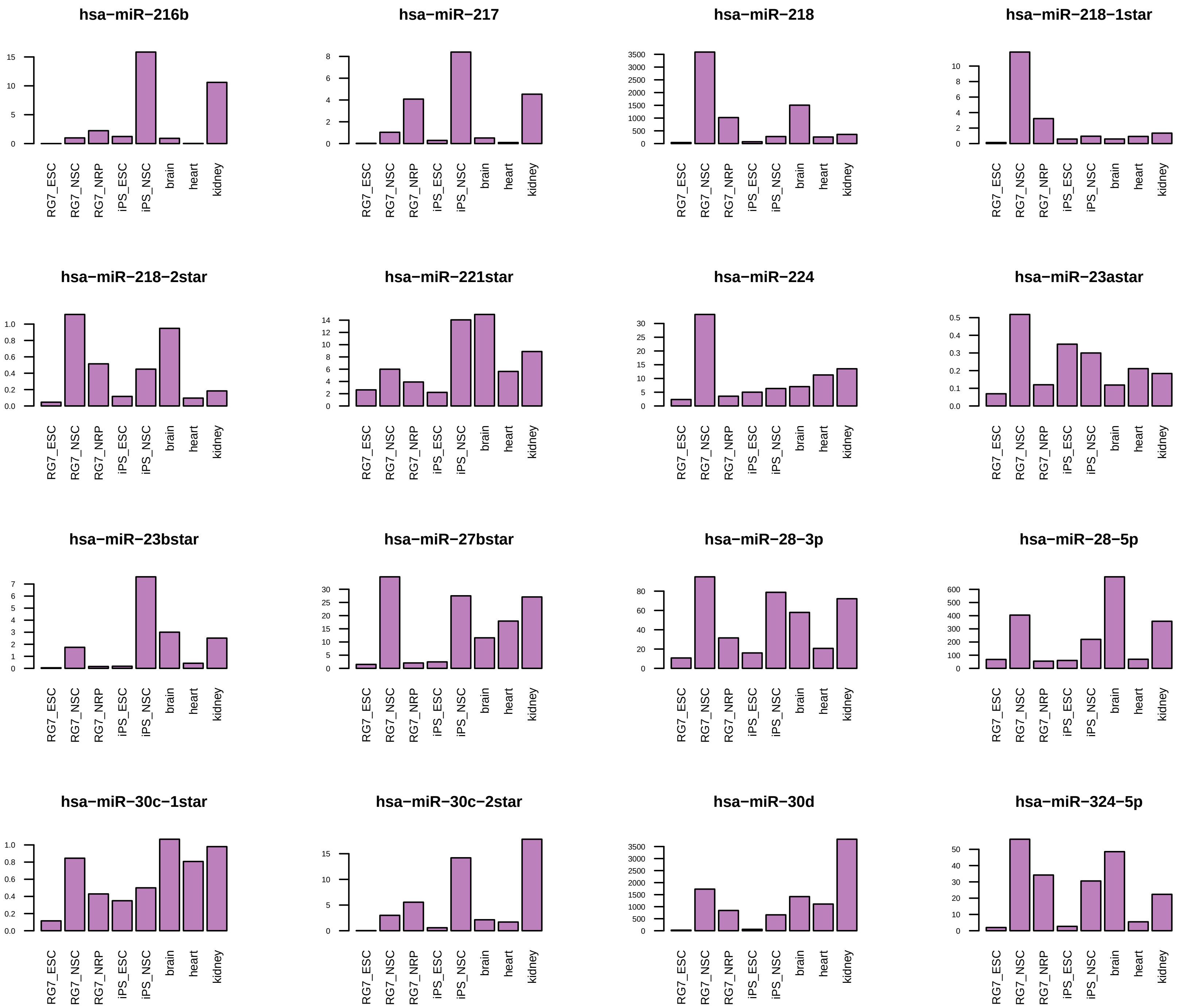
hsa−miR−216b
hsa−miR−217
hsa−miR−218
hsa−miR−218−1star
hsa−miR−218−2star
hsa−miR−221star
hsa−miR−224
hsa−miR−23astar
hsa−miR−23bstar
hsa−miR−27bstar
hsa−miR−28−3p
hsa−miR−28−5p
hsa−miR−30c−1star
hsa−miR−30c−2star
hsa−miR−30d
hsa−miR−324−5p
RG7_ESC
RG7_NSC
RG7_NRP
iPS_ESC
iPS_NSC
brain
heart
kidney

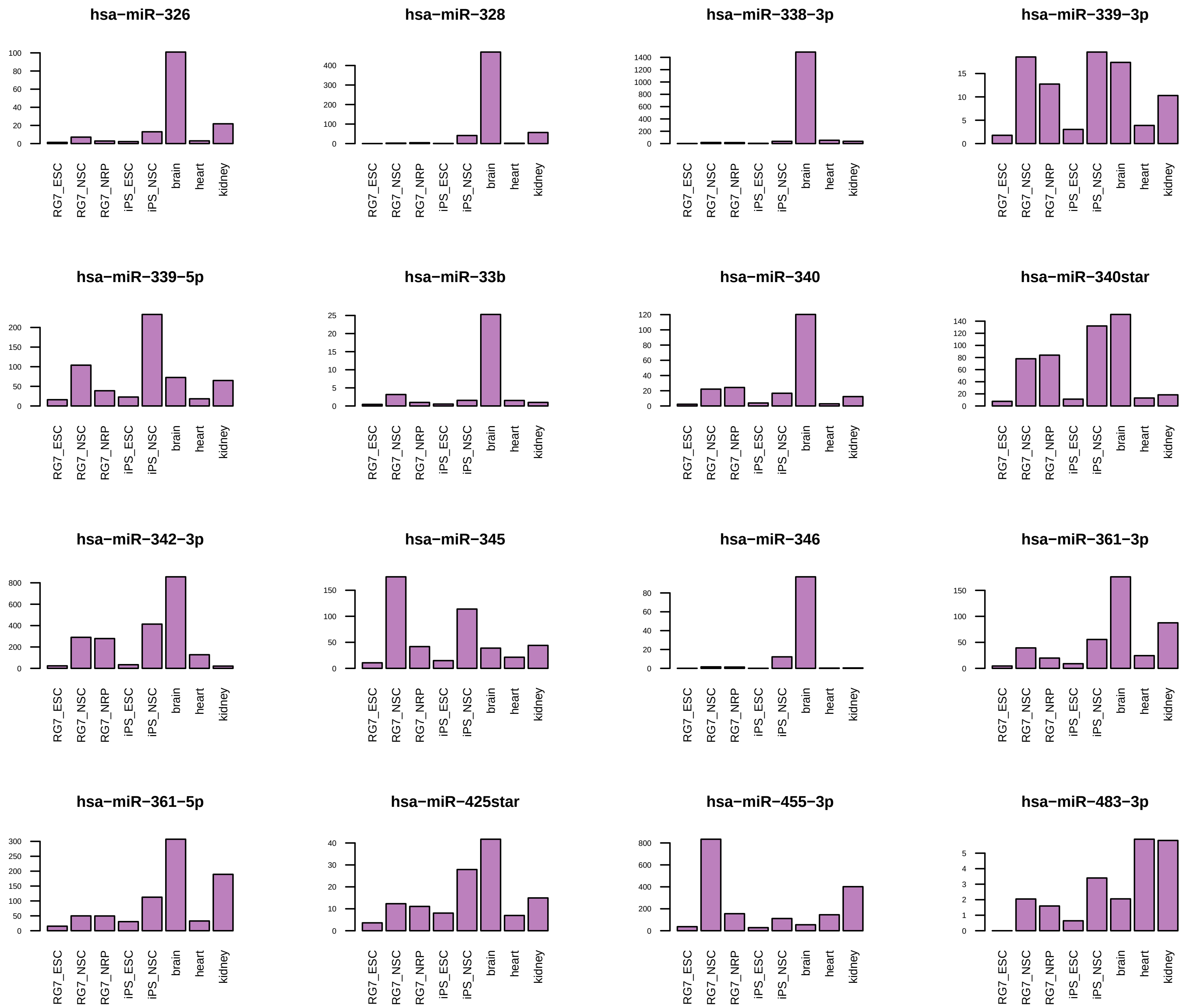
hsa−miR−326
0 20 40 60 80 100
hsa−miR−328
0 100 200 300 400
hsa−miR−338−3p
0 200 400 600 800 1000 1200 1400
hsa−miR−339−3p
0 5 10 15
hsa−miR−339−5p
0 50 100 150 200
hsa−miR−33b
0 5 10 15 20 25
hsa−miR−340
0 20 40 60 80 100 120
hsa−miR−340star
0 20 40 60 80 100 120 140
hsa−miR−342−3p
0 200 400 600 800
hsa−miR−345
0 50 100 150
hsa−miR−346
0 20 40 60 80
hsa−miR−361−3p
0 50 100 150
hsa−miR−361−5p
0 50 100 150 200 250 300
hsa−miR−425star
0 10 20 30 40
hsa−miR−455−3p
0 200 400 600 800
hsa−miR−483−3p
0 1 2 3 4 5
RG7_ESC
RG7_NSC
RG7_NRP
iPS_ESC
iPS_NSC
brain
heart
kidney

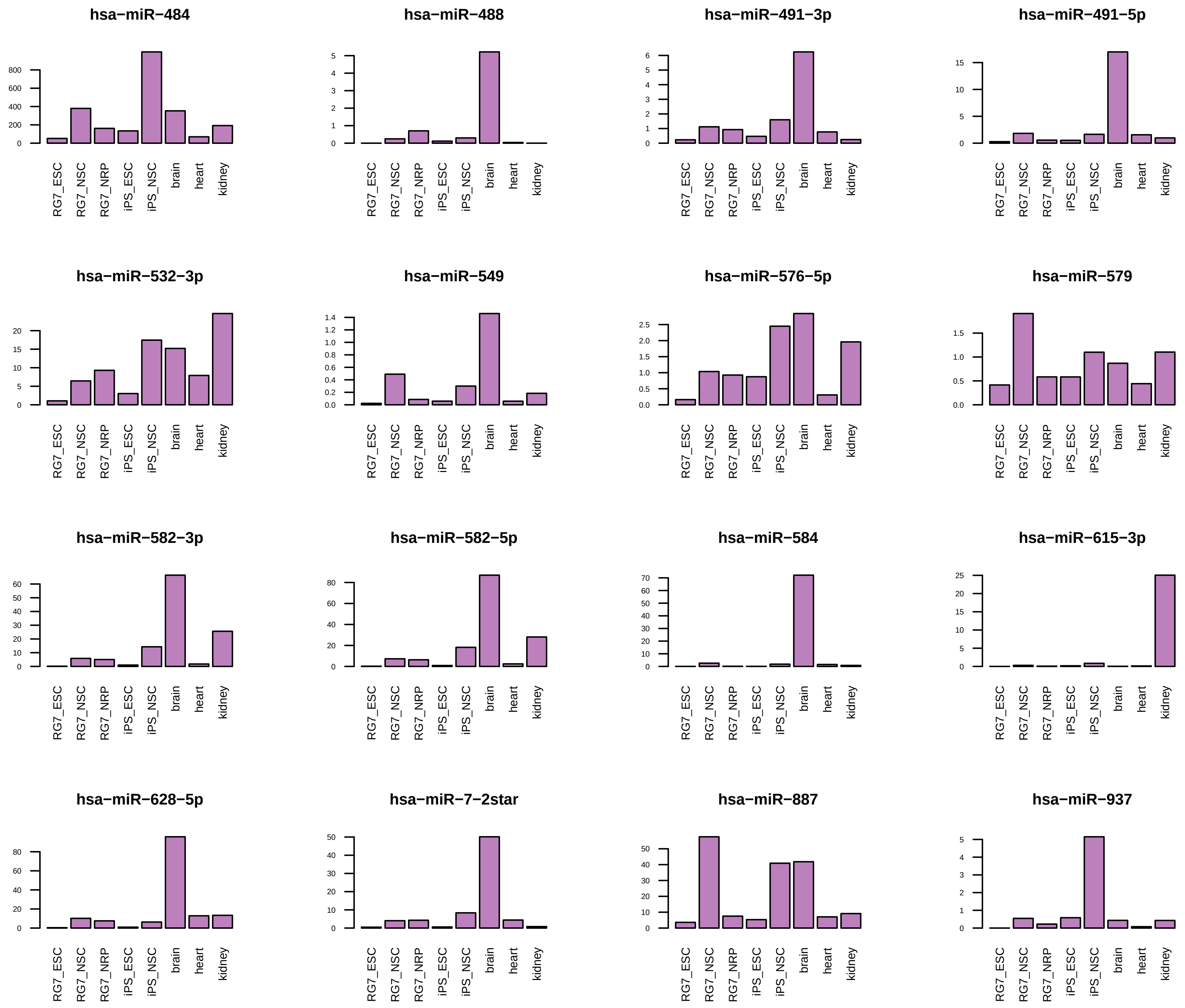
hsa−miR−484
0 200 400 600 800
hsa−miR−488
0 1 2 3 4 5
hsa−miR−491−3p
0 1 2 3 4 5 6
hsa−miR−491−5p
0 5 10 15
hsa−miR−532−3p
0 5 10 15 20
hsa−miR−549
0.0 0.2 0.4 0.6 0.8 1.0 1.2 1.4
hsa−miR−576−5p
0.0 0.5 1.0 1.5 2.0 2.5
hsa−miR−579
0.0 0.5 1.0 1.5
hsa−miR−582−3p
0 10 20 30 40 50 60
hsa−miR−582−5p
0 20 40 60 80
hsa−miR−584
0 10 20 30 40 50 60 70
hsa−miR−615−3p
0 5 10 15 20 25
hsa−miR−628−5p
0 20 40 60 80
hsa−miR−7−2star
0 10 20 30 40 50
hsa−miR−887
0 10 20 30 40 50
hsa−miR−937
0 1 2 3 4 5
RG7_ESC
RG7_NSC
RG7_NRP
iPS_ESC
iPS_NSC
brain
heart
kidney

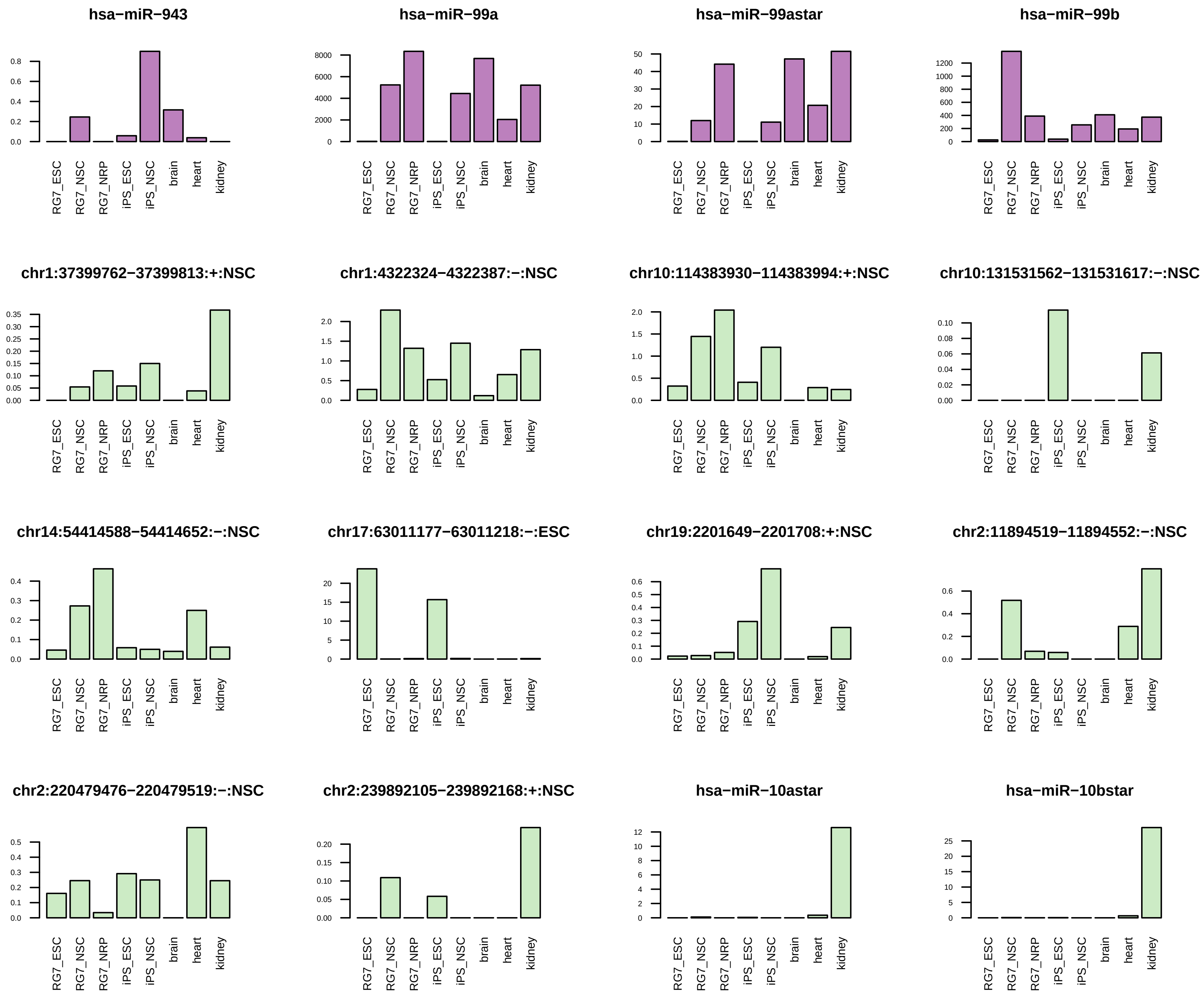
hsa−miR−943
0.0
0.2
0.4
0.6
0.8
hsa−miR−99a
0
2000
4000
6000
8000
hsa−miR−99astar
0
10
20
30
40
50
hsa−miR−99b
0
200
400
600
800
1000
1200
chr1:37399762−37399813:+:NSC
0.00
0.05
0.10
0.15
0.20
0.25
0.30
0.35
chr1:4322324−4322387:−:NSC
0.0
0.5
1.0
1.5
2.0
chr10:114383930−114383994:+:NSC
0.0
0.5
1.0
1.5
2.0
chr10:131531562−131531617:−:NSC
0.00
0.02
0.04
0.06
0.08
0.10
chr14:54414588−54414652:−:NSC
0.0
0.1
0.2
0.3
0.4
chr17:63011177−63011218:−:ESC
0
5
10
15
20
chr19:2201649−2201708:+:NSC
0.0
0.1
0.2
0.3
0.4
0.5
0.6
chr2:11894519−11894552:−:NSC
0.0
0.2
0.4
0.6
chr2:220479476−220479519:−:NSC
0.0
0.1
0.2
0.3
0.4
0.5
chr2:239892105−239892168:+:NSC
0.00
0.05
0.10
0.15
0.20
hsa−miR−10astar
0
2
4
6
8
10
12
hsa−miR−10bstar
0
5
10
15
20
25
RG7_ESC
RG7_NSC
RG7_NRP
iPS_ESC
iPS_NSC
brain
heart
kidney

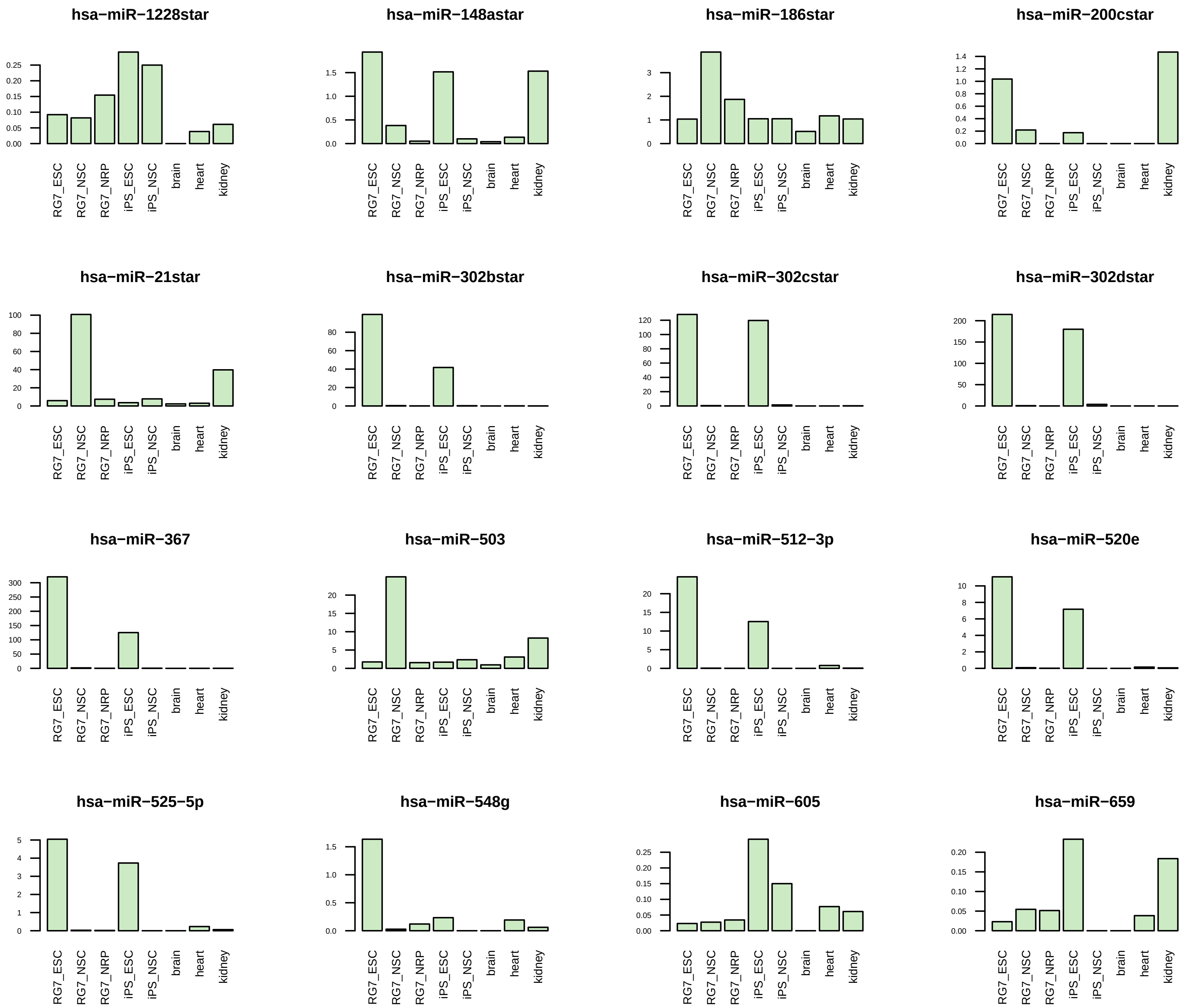
hsa−miR−1228star
0.00 0.05 0.10 0.15 0.20 0.25
RG7_ESC RG7_NSC RG7_NRP iPS_ESC iPS_NSC brain heart kidney
hsa−miR−148astar
0.0 0.5 1.0 1.5
RG7_ESC RG7_NSC RG7_NRP iPS_ESC iPS_NSC brain heart kidney
hsa−miR−186star
0 1 2 3
RG7_ESC RG7_NSC RG7_NRP iPS_ESC iPS_NSC brain heart kidney
hsa−miR−200cstar
0.0 0.2 0.4 0.6 0.8 1.0 1.2 1.4
RG7_ESC RG7_NSC RG7_NRP iPS_ESC iPS_NSC brain heart kidney
hsa−miR−21star
0 20 40 60 80 100
RG7_ESC RG7_NSC RG7_NRP iPS_ESC iPS_NSC brain heart kidney
hsa−miR−302bstar
0 20 40 60 80
RG7_ESC RG7_NSC RG7_NRP iPS_ESC iPS_NSC brain heart kidney
hsa−miR−302cstar
0 20 40 60 80 100 120
RG7_ESC RG7_NSC RG7_NRP iPS_ESC iPS_NSC brain heart kidney
hsa−miR−302dstar
0 50 100 150 200
RG7_ESC RG7_NSC RG7_NRP iPS_ESC iPS_NSC brain heart kidney
hsa−miR−367
0 50 100 150 200 250 300
RG7_ESC RG7_NSC RG7_NRP iPS_ESC iPS_NSC brain heart kidney
hsa−miR−503
0 5 10 15 20
RG7_ESC RG7_NSC RG7_NRP iPS_ESC iPS_NSC brain heart kidney
hsa−miR−512−3p
0 5 10 15 20
RG7_ESC RG7_NSC RG7_NRP iPS_ESC iPS_NSC brain heart kidney
hsa−miR−520e
0 2 4 6 8 10
RG7_ESC RG7_NSC RG7_NRP iPS_ESC iPS_NSC brain heart kidney
hsa−miR−525−5p
0 1 2 3 4 5
RG7_ESC RG7_NSC RG7_NRP iPS_ESC iPS_NSC brain heart kidney
hsa−miR−548g
0.0 0.5 1.0 1.5
RG7_ESC RG7_NSC RG7_NRP iPS_ESC iPS_NSC brain heart kidney
hsa−miR−605
0.00 0.05 0.10 0.15 0.20 0.25
RG7_ESC RG7_NSC RG7_NRP iPS_ESC iPS_NSC brain heart kidney
hsa−miR−659
0.00 0.05 0.10 0.15 0.20
RG7_ESC RG7_NSC RG7_NRP iPS_ESC iPS_NSC brain heart kidney

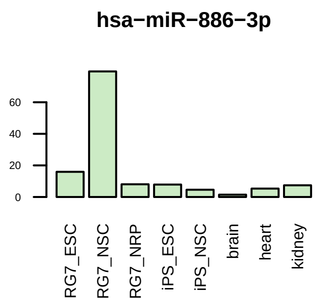
hsa−miR−886−3p
60
40
20
0
RG7_ESC
RG7_NSC
RG7_NRP
iPS_ESC
iPS_NSC
brain
heart
kidney

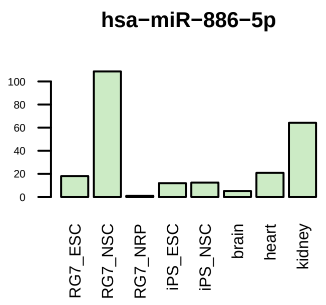
hsa−miR−886−5p
100
80
60
40
20
0
RG7_ESC
RG7_NSC
RG7_NRP
iPS_ESC
iPS_NSC
brain
heart
kidney

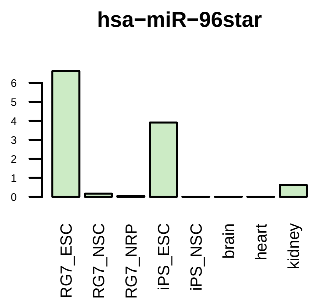
hsa−miR−96star
6
5
4
3
2
1
0
RG7_ESC
RG7_NSC
RG7_NRP
iPS_ESC
iPS_NSC
brain
heart
kidney
